# The DBD-α4 helix of EWSR1::FLI1 is required for GGAA microsatellite binding that underlies genome regulation in Ewing sarcoma

**DOI:** 10.7554/eLife.95626

**Published:** 2026-06-15

**Authors:** Ariunaa Bayanjargal, Cenny Taslim, Iftekhar A Showpnil, Julia Selich-Anderson, Jesse C Crow, Runwei Zhou, Stephen L Lessnick, Emily Rose Theisen

**Affiliations:** 1 Center for Childhood Cancer, Abigail Wexner Research Institute at Nationwide Children’s Hospital Columbus United States; 2 Medical Scientist Training Program, The Ohio State University Columbus United States; 3 Biomedical Sciences Graduate Program, The Ohio State University Columbus United States; 4 Ohio State Biochemistry Program Columbus United States; 5 Department of Pediatrics, The Ohio State Univeristy Columbus United States; 6 Division of Pediatric Heme/Onc/BMT, The Ohio State University College of Medicine Columbus United States; Institut de génétique et de biologie moléculaire et cellulaire France; University of Colorado Anschutz Medical Campus United States

**Keywords:** Ewing sarcoma, genome regulation, transcription, fusion oncogene, Human

## Abstract

Ewing sarcoma is the second most common bone cancer in children and young adults. In 85% of patients, a translocation between chromosomes 11 and 22 results in a potent fusion oncoprotein, EWSR1::FLI1. EWSR1::FLI1 is the only genetic alteration in an otherwise unaltered genome of Ewing sarcoma tumors. The EWSR1 portion of the protein is an intrinsically disordered domain involved in transcriptional regulation by EWSR1::FLI1. The FLI portion of the fusion contains a DNA binding domain shown to bind core GGAA motifs and GGAA repeats. A small alpha-helix in the DNA binding domain of FLI1, DBD-α4 helix, is critical for the transcription function of EWSR1::FLI1. In this study, we aimed to understand the mechanism by which the DBD-α4 helix promotes transcription and therefore oncogenic transformation. We utilized a multi-omics approach to assess chromatin organization, active chromatin marks, genome binding, and gene expression in cells expressing EWSR1::FLI1 constructs with and without the DBD-α4 helix. Our studies revealed DBD-α4 helix is crucial for cooperative binding of EWSR1::FLI1 at GGAA microsatellites. This binding underlies many aspects of genome regulation by EWSR1::FLI1, such as formation of topologically associated domains (TADs), chromatin loops, enhancers, and productive transcription hubs.

## Introduction

Ewing sarcoma is an aggressive bone-associated tumor currently treated with dose-intense chemotherapy, radiation, and surgery (Pappo and Dirksen, 2018). It affects adolescents and young adults with an incidence rate of 3 per million (Ozaki, 2015). Of these patients, 25–35% have overt metastatic disease with a recurrence rate of 50–80% (Gaspar et al., 2015). Roughly a quarter of patients with localized disease also relapse (Van Mater and Wagner, 2019). The 5-year survival rate of metastatic and relapsed patients is only 10–30% (Stahl et al., 2011). Treatment options for relapsed/metastatic Ewing sarcoma patients have not improved for the last four decades. The lack of efficient and targeted treatment for Ewing sarcoma can be attributed to our poor understanding of how precisely Ewing sarcoma is driven by a fusion oncoprotein called EWSR1::FLI1.

Expression of EWSR1::FLI1 results from a translocation between chromosomes 11 and 22 that fuses two genes, *EWSR1* and *FLI1* (Delattre et al., 1992). As a result, the transcription activation domain of EWSR1 and the DNA binding domain of FLI1 fuse together to create a potent transcription factor (May et al., 1993). The EWSR1 domain is highly disordered and is important for multimerization and transcriptional hub formation (Chong et al., 2018). The FLI portion contains a DNA binding domain that binds at two distinct sites: canonical FLI1 binding sites containing a GGAA core and microsatellites containing GGAA repeats (Gangwal et al., 2008). At these GGAA microsatellites, EWSR1::FLI1 binding creates de novo enhancers driving genome-wide reprogramming of enhancers to Ewing-specific enhancers (Riggi et al., 2014). Regulation of Ewing-specific enhancers underlies the mechanisms by which EWSR1::FLI1 regulates expression of thousands of target genes (Grünewald et al., 2015). More specifically, activation of genes involved in proliferation, migration, and invasion pathways leads to oncogenic transformation of precursor cells to Ewing sarcoma cells (Kauer et al., 2009).

In our recent studies of EWSR1::FLI1, we found a small alpha-helix in the DNA binding domain, DBD-α4, to be required for transcription and regulation by the fusion protein (Boone et al., 2021). Interestingly, this study did not find any change in chromatin accessibility (ATAC-Seq) and genome localization of EWSR1::FLI1 constructs (CUT&RUN) when the DBD-α4 helix was deleted, leaving the mechanistic basis for the requirement of the DBD-α4 in transcription regulation unclear. In parallel studies, we also observed that EWSR1::FLI1 expression results in widespread changes to 3D chromatin organization (Showpnil et al., 2022). These results together prompted us to consider whether the DBD-α4 helix is contributing toward EWSR1::FLI1’s abilities to make changes in the epigenome and the local chromatin structure. To assess whether the DBD-α4 helix is important in chromatin organization, we used our ‘knockdown/rescue’ system in A-673 and TTC-466 cells (Theisen et al., 2019; Boone et al., 2021; Showpnil et al., 2022) in conjunction with genomics techniques, including RNA-Seq, CUT&Tag, and Micro-C. These sets of experiments allowed us to characterize the involvement of the DBD-α4 helix in modulating the chromatin organization, epigenetic reprogramming of enhancers, binding at GGAA microsatellites, and promoting transcription leading to transformation.

## Results

### The DBD-α4 helix of FLI1 domain is implicated in restructuring of 3D chromatin in A-673 cells

**Figure 1.**
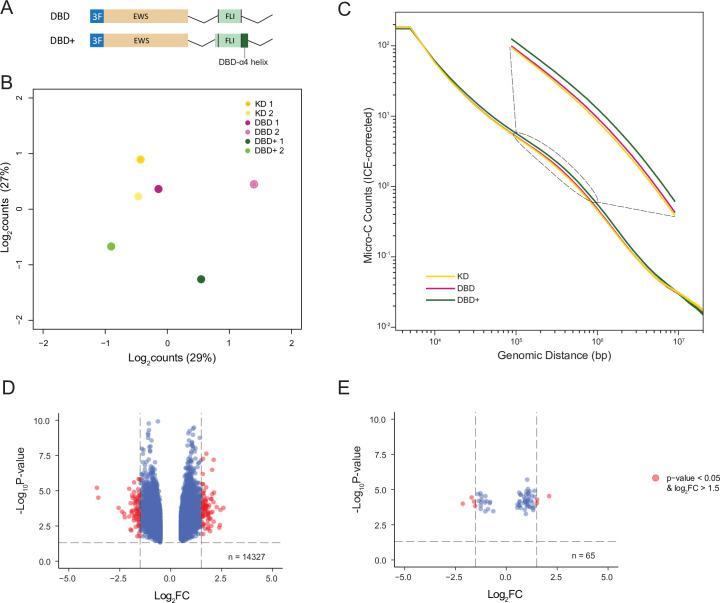
The DBD-α4 helix of FLI1 domain is required to restructure chromatin in A-673 cell. (**A**) A schematic of DBD and DBD+ constructs used in shRNA knockdown and rescue experiments. (**B**) Multidimensional scaling (MDS) plot of top 1000 interactions (500 kb resolution) in each biological replicate. (**C**) Genome-wide interaction frequency (ICE-corrected Micro-C counts) over genomic distance (bp) at 5 kb resolution. (**D**) Volcano plot showing differentially interacting regions (DIRs) detected at 500 kb resolution for DBD+ replicates versus KD replicates. (**E**) Volcano plot showing DIRs detected at same resolution for DBD replicates versus KD replicates. Boxplots depict the minimum, first quartile, median, third quartile, and maximum. *p-Value<0.05, p-value<0.001.

To assess the mechanism by which the DBD-α4 promotes transcription, we depleted endogenous EWSR1::FLI1 expression with shRNA (KD) and rescued with previously published DBD and DBD+ EWSR1::FLI1 constructs with 3X-FLAG tags in A-673 cells (Figure 1A, Boone et al., 2021). The main difference between DBD and DBD+ constructs is the presence of DBD-α4 helix in DBD+ (Figure 1—figure supplement 1A). We recapitulated our previous findings that DBD is unable to rescue the same number of Ewing sarcoma-specific genes as DBD+, therefore incapable of driving oncogenic transformation (Figure 1—figure supplement 1B–D, Boone et al., 2021).

**Figure 1—figure supplement 1.**
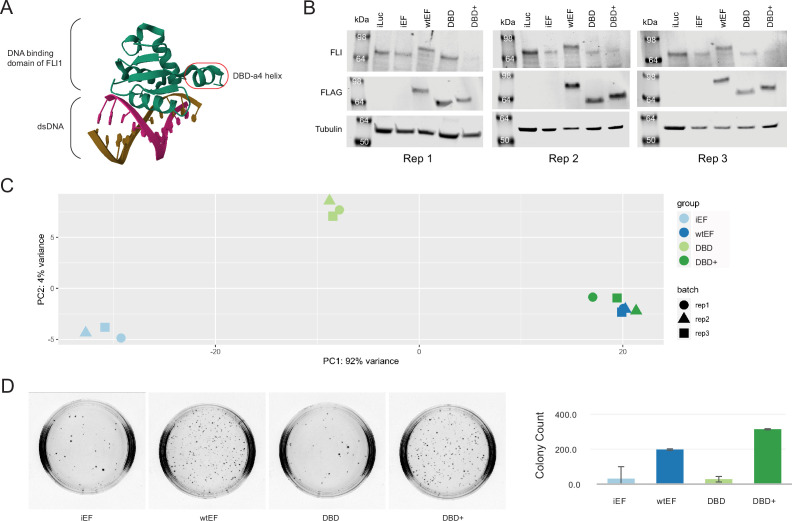
DBD-a4 helix of FLI1 domain is required for EWSR1::FLI1-driven transcriptional regulation and oncogenic transformation in A-673 cells. (**A**) The DBD-α4 helix of FLI1 depicted on dsDNA (PDB). (**B**) Knockdown of endogenous EWSR1::FLI1 detected with FLI1 ab and rescue of wtEF, DBD, and DBD+ detected with FLAG ab in A-673 cells. (**C**) A principal component analysis (PCA) plot of RNA-seq experiments in A-673 cells. (**D**) Representative image of soft agar colony plates and quantification of three biological replicates. Bar charts display mean and standard deviation. Figure 1—figure supplement 1—source data 1.PDF file containing original western blots for Figure 1—figure supplement 1B, indicating the relevant bands and treatments. Figure 1—figure supplement 1—source data 2.Original files for western blot analysis displayed in Figure 1—figure supplement 1B. Figure 1—figure supplement 1—source data 3.PDF files containing original agar gels for Figure 1—figure supplement 1D, indicating the technical replicates and treatments. Figure 1—figure supplement 1—source data 4.Original files for agar gels displayed in Figure 1—figure supplement 1D.

**Figure 1—figure supplement 2.**
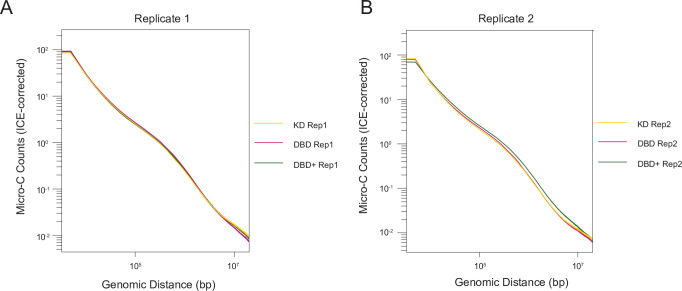
Genome-wide Micro-C interaction frequency profiles across biological replicates in A-673 cells. (**A**) Genome-wide interaction frequency (ICE-corrected Micro-C counts) over genomic distance (bp) at 5 kb resolution for replicate 1 of A-673. (**B**) Genome-wide interaction frequency (ICE-corrected Micro-C counts) over genomic distance (bp) at 5 kb resolution for replicate 2 of A-673.

**Figure 1—figure supplement 3.**
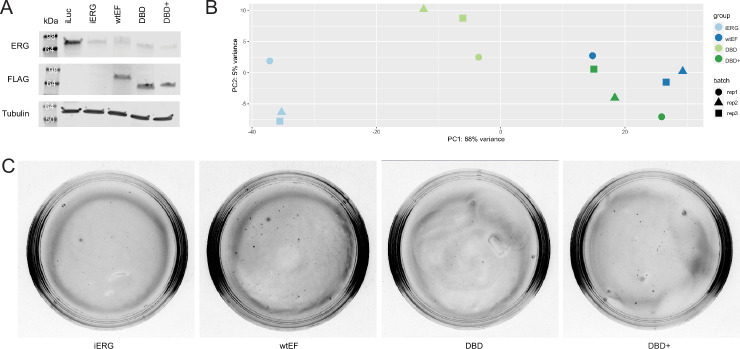
DBD-a4 helix of FLI1 domain is required for EWSR1::FLI1-driven transcriptional regulation and oncogenic transformation in TTC-466 cells. (**A**) Knockdown of endogenous EWSR1::ERG detected with ERG ab and rescue of wtEF, DBD, and DBD+ detected with FLAG ab in TTC-466 cells. (**B**) A principal component analysis (PCA) plot of RNA-seq experiments in TTC-466 cells. (C) Representative images of soft agar colony plates. Figure 1—figure supplement 3—source data 1.PDF file containing original western blots for Figure 1—figure supplement 3A, indicating the relevant bands and treatments. Figure 1—figure supplement 3—source data 2.Original files for western blot analysis displayed in Figure 1—figure supplement 3A. Figure 1—figure supplement 3—source data 3.PDF files containing original agar gels for Figure 1—figure supplement 3C, indicating the technical replicates and treatments. Figure 1—figure supplement 3—source data 4.Original files for agar gels displayed in Figure 1—figure supplement 3C.

**Figure 1—figure supplement 4.**
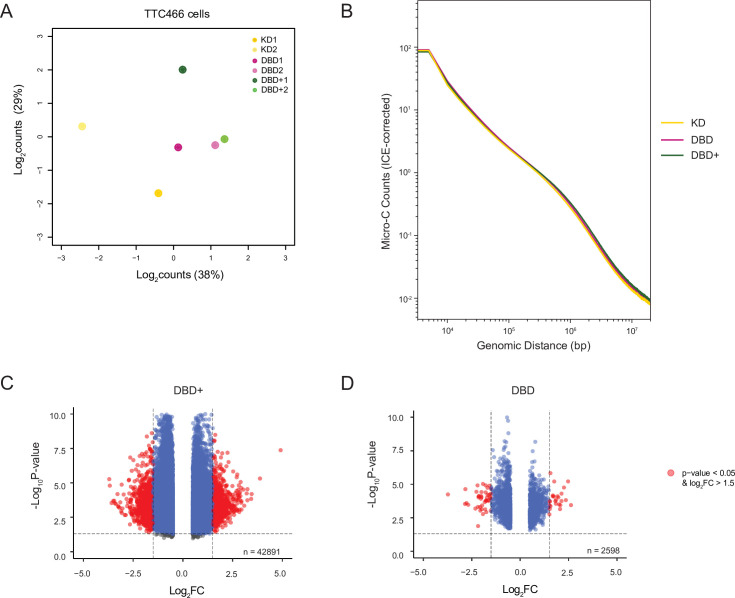
DBD-a4 helix of FLI1 domain is required to restructure chromatin in TTC-466 cells. (**A**) Multidimensional scaling (MDS) plot of top 1000 interactions (500 kb resolution) in each biological replicate. (**B**) Genome-wide interaction frequency of combined replicates (ICE-corrected Micro-C counts) over genomic distance (bp) at 5 kb resolution. (**C**) Volcano plot showing differentially interacting regions (DIRs) detected at 500 kb resolution for DBD+ replicates versus KD replicates. (**D**) Volcano plot showing DIRs detected at same resolution for DBD replicates versus KD replicates.

Next, we sought to understand the role of DBD-α4 helix in 3D chromatin organization. We carried out Micro-C experiments in KD, DBD, and DBD+ cells. First, we performed multidimensional scaling (MDS) analysis of the top 1000 interactions of Micro-C matrices at 500 kb resolution to assess the global regulation of 3D chromatin organization (Figure 1B). KD replicates clustered together with DBD replicate 1 on both axes and with DBD replicate 2 on y-axis. DBD+ replicates, on the other hand, clustered away from both KD and DBD replicates. These observations suggest that the global chromatin structure of DBD replicates is more similar to KD than DBD+ replicates. We also assessed the contact frequency across the genome in KD, DBD, and DBD+ cells at 5 kb resolution (Figure 1C). We found that at the mid-region of the plot (10^5^−10^6^ bp), DBD+ cells make more contacts in comparison to KD and DBD cells. This pattern of contact frequency was also detected in replicate 2, but not in replicate 1 when plotted separately (Figure 1—figure supplement 2). At this genome-wide level, we observe that DBD cells display similar contact frequency as KD cells, suggesting that the DBD-α4 helix participates in EWSR1::FLI1-mediated 3D chromatin restructuring.

To determine if these changes were significant, we conducted differential interaction analysis using 500 kb matrices to compare DBD and DBD+ biological replicates to KD replicates. First, we detected 14,326 and 65 interactions in DBD+ and DBD cells, respectively, using cutoff values of adjusted p-value<0.2 and log_2_FC>0.5. Then, we used cutoff values of adjusted p-value<0.05 and log_2_FC>1.5 to identify differentially interacting regions (DIRs). We found that DBD+ cells have 151 DIRs compared to KD cells (Figure 1D), whereas DBD cells only have 7 DIRs (Figure 1E). Taken together, these data demonstrate that the DBD-α4 helix of FLI1 domain is potentially required for EWSR1::FLI1 to restructure 3D chromatin in A-673 cells.

### Altered TAD structure in A-673 DBD cells is linked to GGAA microsatellite binding

**Figure 2.**
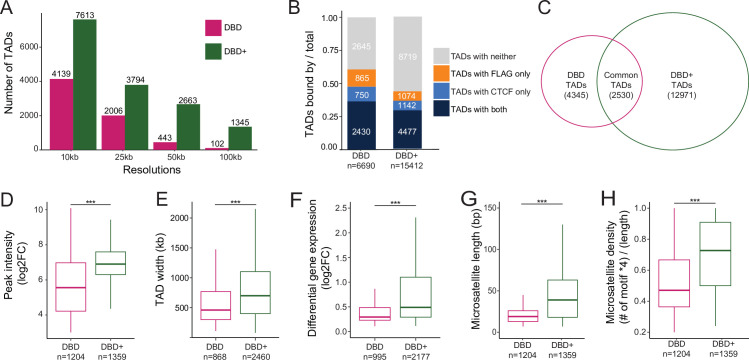
Altered topologically associated domain (TAD) structure in A-673 DBD cells is linked to GGAA microsatellite binding. (**A**) Number of TADs detected in DBD and DBD+ compared to KD at resolutions of 10 kb, 25 kb, 50 kb, and 100 kb. (**B**) Proportion of TADs (compared to KD) bound by FLAG, CTCF, both, or neither. (**C**) Venn diagram of overlap between DBD and DBD+ TADs (compared to KD). (**D–H**) Comparison of DBD and DBD+ unique TADs. (**D**) Binding intensity of unique FLAG peaks (FDR<0.05, FC>8, counts>80, IDR<0.01) across the width of DBD and DBD+ unique TADs. (**E**) Width of DBD and DBD+ unique TADs in bp. (**F**) Expression level of significantly upregulated genes within unique TADs in DBD and DBD+ bound by FLAG. (**G**) Length of microsatellites bound by unique FLAG peaks in DBD and DBD+ conditions in bp. (**H**) Density of GGAA motif in the microsatellites calculated as (# of motif × 4)/(length of microsatellites) in DBD and DBD+ unique TADs bound by unique FLAG peaks. Boxplots depict the minimum, first quartile, median, third quartile, and maximum. p-Value<0.001.

To investigate the necessity of the DBD-α4 helix for higher-order chromatin organization, we carried out topologically associated domain (TAD) analysis in A-673 cells using HiCExplorer suite of tools (Wolff et al., 2020). TADs are functional subunits of chromatin that define regulatory landscape (Szabo et al., 2019). Within these domains, chromatin interactions, including those involving regulatory elements, are spatially confined, often by insulators (Dixon et al., 2012; Hou et al., 2012; Nora et al., 2012). Moreover, genes located in the same TAD are typically co-regulated together (Dixon et al., 2016; Ramírez et al., 2018), and this co-regulation is facilitated by interactions between enhancers and promoters, which also tend to occur within the confines of a same TAD (Bonev et al., 2017). Our initial step involved identifying TADs in both DBD and DBD+ matrices with hicFindTADs at 10 kb, 25 kb, 50 kb, and 100 kb resolutions. Subsequently, we computed differential TADs with hicDifferentialTAD. This analysis entailed a comparison of precomputed TAD regions of DBD and DBD+ matrices with the same regions of KD matrix. The purpose was to detect TADs that appeared specifically in response to the expression of DBD and DBD+ constructs in comparison to KD sample (Figure 2A). At each resolution, the number of TADs detected when DBD+ was expressed was far greater than when DBD was expressed.

**Figure 2—figure supplement 1.**
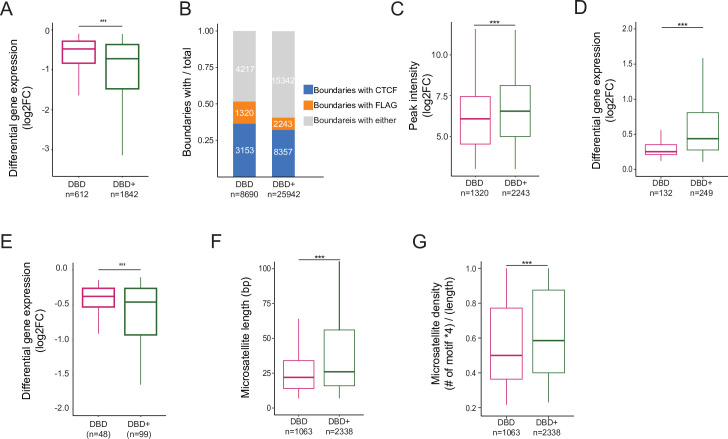
DBD-a4 helix of FLI1 domain influences topologically associated domain (TAD) boundary formation, GGAA microsatellite occupancy, and associated transcriptional regulation in A-673 cells. (**A**) Expression level of significant genes overlapped with unique topologically associated domains (TADs) in DBD (mean = −0.64) and DBD+ (mean = −1.16) bound by FLAG at GGAA microsatellites. (**B**) Proportion of TAD boundaries bound by FLAG, CTCF, or neither. (**C–G**) Comparison of DBD and DBD+ unique TAD boundaries. (**C**) Binding intensity of unique FLAG peaks (FDR<0.05, FC>8, counts>80, IDR<0.01) at boundaries of DBD and DBD+ unique TADs. (**D**) Expression level of significantly upregulated genes overlapped with boundaries of unique TADs in DBD and DBD+ bound by FLAG at GGAA microsatellites. (**E**) Expression level of significantly downregulated genes overlapped with boundaries of unique TADs in DBD and DBD+ bound by FLAG at GGAA microsatellites. (**F**) Length of microsatellites bound by unique FLAG peaks at the boundaries of DBD and DBD+ conditions in bp. (**G**) Percent of GGAA motif in the microsatellites calculated as (# of motif × 4)/(length of microsatellites) at the boundaries of DBD and DBD+ unique TADs bound by unique FLAG peaks. All data are from A-673 cells. Boxplots depict the minimum, first quartile, median, third quartile, and maximum. p-Value<0.001.

**Figure 2—figure supplement 2.**
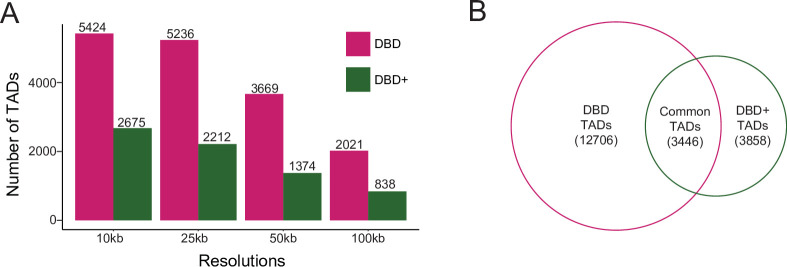
Topologically associated domain (TAD) analysis in TTC-466 cells. (**A**) Number of TADs detected in DBD and DBD+ compared to KD at resolutions of 10 kb, 25 kb, 50 kb, and 100 kb. (**B**) Venn diagram of overlap between DBD and DBD+ TADs (compared to KD).

To exclude duplicated TADs when combining TADs from different resolutions, we used the default threshold value of 5000 bp as the minimum boundary difference required to consider two TADs as distinct. With this approach, we found 6690 total TADs for DBD and 15,412 total TADs for DBD+ (Figure 2B). To understand the local biological context of these total TADs in DBD and DBD+ cells, we annotated the whole width of the TADs with CTCF and FLAG CUT&Tag peaks (FDR<0.05, FC>8, counts>80, IDR<0.01) (Figure 2B). In DBD cells, we detected 11.2% of the TADs bound by CTCF only, 12.9% bound by DBD FLAG only, 36.3% bound by both CTCF and DBD FLAG, and 39.6% bound by neither. In contrast, for DBD+, we detected 7.4% and 7.0% of the TADs bound by only CTCF and only DBD+ FLAG, respectively, 29.0% bound by both, and 56.6% bound by neither. These observations highlight both the increased number of total TADs detected in DBD+ cells and a notable increase in the percentage of TADs detected in DBD+ cells with no CTCF or FLAG association. This suggests a broader role for the DBD-α4 helix in genome-wide reorganization of TADs by EWSR1::FLI1, consistent with the results discussed above relating to Figure 1.

Next, we overlapped total TADs from DBD and DBD+ conditions and identified 2530 TADs common to both. There were 4345 unique TADs in DBD cells and 12,971 unique TADs in DBD+ cells (Figure 2C). To understand the transcriptional activity driven by the DBD-α4 helix at GGAA repeats, we focused on the whole width of unique TADs containing DBD and DBD+ FLAG peaks at GGAA microsatellites (Figure 2D–H). This analysis narrowed the TADs down to 868 unique ones for DBD cells and 2460 unique TADs for DBD+ cells (Figure 2E). We observed that within these TAD subsets containing FLAG peaks at GGAA microsatellites, the intensity of the DBD+ FLAG peaks was higher compared to DBD FLAG peaks (Figure 2D). Moreover, TADs uniquely bound by DBD+ at GGAA microsatellites were not only wider (Figure 2E) but also contained a higher number of differentially expressed genes compared to those bound only by DBD (Figure 2F, Figure 2—figure supplement 1A). Further analysis of GGAA microsatellites bound by DBD and DBD+ peaks within unique TADs revealed that the DBD+ FLAG peaks were associated with longer microsatellites (Figure 2G) and denser GGAA motifs (Figure 2H, density calculated by multiplying the number of GGAA motifs by 4 and dividing by the total length of the microsatellites) compared with those bound by DBD alone. These findings suggest that DBD+ accesses longer and denser microsatellites within larger TADs and binds to these sites with higher binding intensity than DBD. Furthermore, these TADs with DBD+ bound microsatellites show greater changes in gene expression, suggesting a functional role for the DBD-α4 helix in gene regulation within TADs.

TAD boundaries are regions between TADs that act as insulators (Dixon et al., 2012) and are often marked by active chromatin (Ay et al., 2014). We conducted a similar analysis as above in a 20 kb region around unique TAD boundaries (Figure 2—figure supplement 1B–G). We observed a comparable percentage of the unique TAD boundaries were bound by only CTCF peaks in DBD and DBD+ cells (36.3% and 32.2%, respectively). The percentages of boundaries with FLAG peaks in DBD and DBD+ cells were 15.2% and 8.6%, respectively (Figure 2—figure supplement 1B). When we considered boundaries that overlap with FLAG peaks at microsatellites (Figure 2—figure supplement 1C–G), we observed similar findings as those spanning the whole width of TADs (Figure 2D and F–H). The FLAG peaks at DBD+ specific TAD boundaries were of higher intensity than FLAG peaks at DBD specific TAD boundaries (Figure 2—figure supplement 1C). Next, we assessed gene expression at TAD boundaries and found DBD+ boundaries contained more significantly regulated genes (Figure 2—figure supplement 1D and E). The GGAA microsatellites bound by DBD+ FLAG peaks at boundaries were also longer and denser than DBD bound microsatellites (Figure 2—figure supplement 1F and G). These data demonstrate that the DBD-α4 helix affects overall DNA binding at GGAA microsatellites and gene expression in the context of TAD organization.

### DBD and DBD+ form loops at GGAA microsatellites, but DBD+ rescues more and shorter loops in A-673 cells

**Figure 3.**
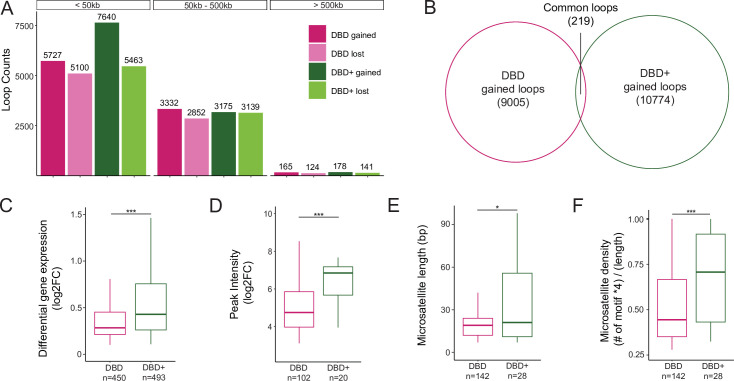
DBD and DBD+ form loops at GGAA microsatellites, but DBD+ rescues more and shorter loops in A-673 cells. (**A**) Number of loops detected in DBD and DBD+ compared to KD at resolutions of 1 kb at short-range (<50 kb), mid-range (50–500 kb), and long-range (>500 kb). (**B**) Venn diagram of overlap between DBD and DBD+ uniquely gained loops (compared to KD). (**C**) Expression level of significant genes overlapped with uniquely gained loop anchors of DBD and DBD+. Means = 0.45 (DBD) and 0.77 (DBD+). (**D**) Peak intensity of unique FLAG peaks (FDR<0.05, FC>8, counts>80, IDR<0.01) at anchors of uniquely gained loops in DBD and DBD+ cells. Means = 5.01 and 6.30 per DBD and DBD+. (E) Length of microsatellites bound by unique FLAG peaks at the anchors of DBD and DBD+ uniquely gained loops in bp. Means = 22.5 bp (DBD) and 38 bp (DBD+). (**F**) Density of GGAA motif in the microsatellites calculated as (# of motif × 4)/(length of microsatellites) at the anchors of DBD and DBD+ uniquely gained loops bound by unique FLAG peaks. Means = 0.51 (DBD) and 0.70 (DBD+). Boxplots depict the minimum, first quartile, median, third quartile, and maximum. *p-Value<0.05, p-value<0.001.

Previously, we observed that EWSR1::FLI1 promotes chromatin loop formation at GGAA microsatellites leading to enhancer activation and upregulation of gene expression (Showpnil et al., 2022). We next asked whether the DBD-α4 helix participates in functions of EWSR1::FLI1 pertaining to the formation of loops by calling chromatin loops using the Mustache algorithm (Roayaei Ardakany et al., 2020) in matrices of A-673 DBD and DBD+ cells compared to KD cells at 1 kb resolution. We detected loops in three ranges defined as short-range (<50 kb), mid-range (50–500 kb), and long-range (>500 kb) loops based on the linear genomic distance between loop anchors (Figure 3A). The only range with a difference between DBD and DBD+ cells was short-range loops. Specifically, we found that DBD+ expressed cells gained 1913 more short-range loops than DBD expressed cells compared to KD. Both conditions had a similar number of short-range lost loops (5100 and 5463 for DBD and DBD+, respectively, Figure 3A). For an overlap analysis between DBD and DBD+ conditions, we included all lost loops and found 5610 loops that were commonly lost in both conditions (Figure 3—figure supplement 1A). When the same overlap analysis was carried out for DBD and DBD+ gained loops of all ranges, we found only 219 common loops (Figure 3B). This small overlap of gained loops suggested that although they have a similar number of total gained loops, DBD and DBD+ cells contain loops with very different anchors. These sets of observations suggest that formation of distinct set of short-range loops is a potential mechanism by which the DBD-α4 helix restructures chromatin organization in A-673 cells.

**Figure 3—figure supplement 1.**
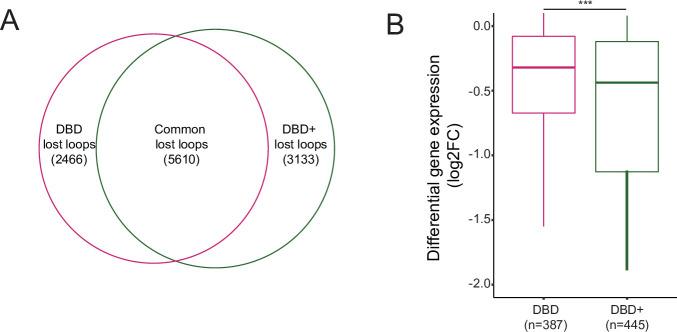
DBD+ loop anchors associated with more gene down regulation compared to DBD specific anchors. (**A**) Venn diagram of overlap between DBD and DBD+ uniquely lost loops (compared to KD). (**B**) Expression level of downregulated genes overlapped with uniquely gained loop anchors of DBD and DBD+. All data are from A-673 cells. Means = –0.68, –1.25, 0.35, –1.08. p-Value<0.05, p-value<0.001. Boxplots depict the minimum, first quartile, median, third quartile, and maximum.

**Figure 3—figure supplement 2.**
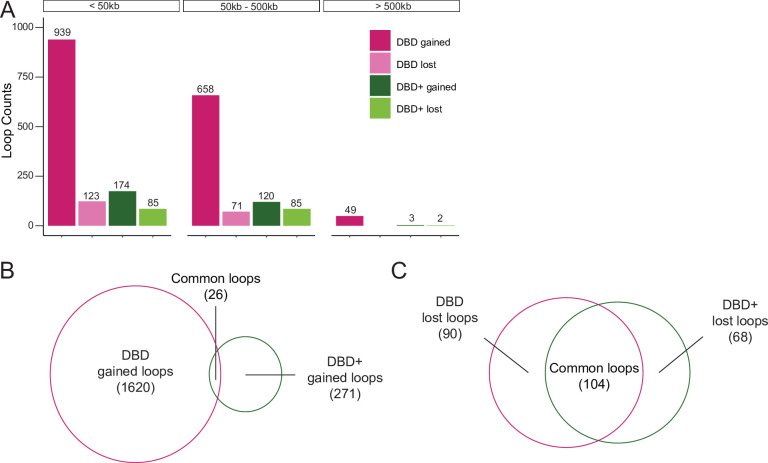
Loop analysis in TTC-466 cells. (**A**) Number of loops detected in DBD and DBD+ compared to KD at resolutions of 1 kb at short-range (<50 kb), mid-range (50–500 kb), and long-range (>500 kb). (**B**) Venn diagram of overlap between DBD and DBD+ uniquely gained loops (compared to KD). (**C**) Venn diagram of overlap between DBD and DBD+ uniquely lost loops (compared to KD).

Prompted by the above difference in short-range loop formation, we further investigated uniquely gained loops of all ranges in DBD (9005) and DBD+ (10,774) conditions (Figure 3B). When individual anchors of loops were overlapped with GGAA microsatellites, we found that 92.4% and 92.2% of DBD and DBD+ loop anchors were at GGAA microsatellites. We then assessed overall gene expression at all uniquely gained loop anchors and found DBD+ loop anchors associated with more gene activation (Figure 3C) and downregulation compared to DBD specific anchors (Figure 3—figure supplement 1B). Next, we focused our analysis on gained loop anchors with FLAG peaks at GGAA microsatellites in DBD and DBD+ cells. We found 102 and 20 FLAG peaks overlapping with DBD and DBD+ loop anchors, respectively (Figure 3D). At this subset, FLAG peaks had a higher intensity in DBD+ compared to DBD-expressed cells. The GGAA microsatellites overlapping with DBD and DBD+ FLAG peaks at gained loop anchors were further characterized in length and density (Figure 3E and F). We found that DBD+ FLAG peaks were bound at GGAA microsatellites that are long and dense in comparison to DBD-bound microsatellites. Taken together, these data indicate that the DBD-α4 helix facilitates the formation of short-range loops by promoting binding at long and dense GGAA microsatellites, thus leading to more gene regulation.

### DBD+ rescues de novo enhancer formation at GGAA microsatellites in A-673 cells

**Figure 4.**
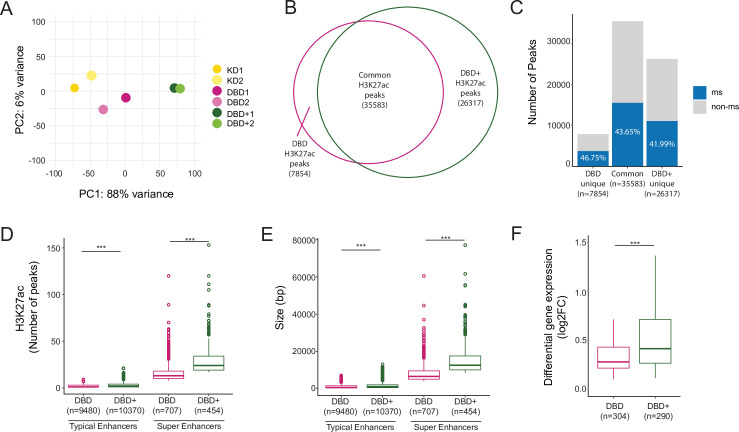
DBD+ rescues de novo enhancer formation at microsatellites A-673 cells. (**A**) Principal component analysis (PCA) plot of H3K27ac peaks in biological replicates of KD, DBD, and DBD+. (**B**) Venn diagram of overlap of H3K27ac peaks (FDR<0.05, FC>8, counts>80, IDR<0.01) between DBD and DBD+. (**C**) Percentage of H3K27ac peaks at microsatellites in common, DBD unique, and DBD+ unique peaks. (**D**) Number of H3K27ac peaks constituting typical and super-enhancers called in DBD and DBD+ conditions. (E) Constituent size (in bp) of typical and super-enhancers in DBD and DBD+ conditions. (**F**) Expression level of significantly upregulated genes at DBD and DBD+ super-enhancers. Boxplots depict the minimum, first quartile, median, third quartile, and maximum. Circles depict outliers. p-Value<0.001.

Multiple studies have shown that binding of EWSR1::FLI1 at GGAA microsatellites results in genome-wide de novo enhancer formation (Riggi et al., 2014; Tomazou et al., 2015; Sheffield et al., 2017). Ewing-specific enhancer establishment at GGAA microsatellites is functionally linked to the oncogenic transformation (Johnson et al., 2017a). We therefore sought to understand whether the DBD-α4 helix of EWSR1::FLI1 has any role in modulating the Ewing-specific enhancer landscape. To assay the enhancer landscape, we collected H3K27ac CUT&Tag data from A-673 KD, DBD, and DBD+ cells. Principal component analysis (PCA) of H3K27ac localization shows that the DBD replicates were clustered closer to the KD replicates while being in between the KD and the DBD+ replicates (Figure 4A), suggesting that the DBD-α4 helix is required to reshape the enhancer landscape. Next, we did overlap analysis of H3K27ac peaks of DBD and DBD+ cells. There were 7854 H3K27ac peaks unique to DBD cells, 35,583 peaks common between DBD and DBD+ cells, and 26,317 peaks unique to DBD+ cells (Figure 4B), highlighting the vastly different enhancer landscapes of DBD and DBD+ conditions. Next, we sought to understand the extent of GGAA microsatellite-driven H3K27 acetylation by conducting overlap analysis of H3K27ac peaks and GGAA microsatellites that were previously characterized (Figure 4C, Johnson et al., 2017a). Interestingly, for each group of H3K27ac peaks we considered (i.e. DBD, common, and DBD+), we found around 40% of the H3K27ac peaks overlapped with GGAA microsatellites. Overall, a significant portion of H3K27ac peaks correlated with GGAA microsatellites in both DBD and DBD+ conditions underscoring the importance of GGAA repeats in the formation of novel enhancers by fusions involving the transcriptional activation domain of EWSR1 with the FLI1 DNA binding domain. However, these results suggest that the DBD-α4 helix is required for modulating the enhancer landscape in Ewing cells to specifically promote oncogenic transformation.

**Figure 4—figure supplement 1.**
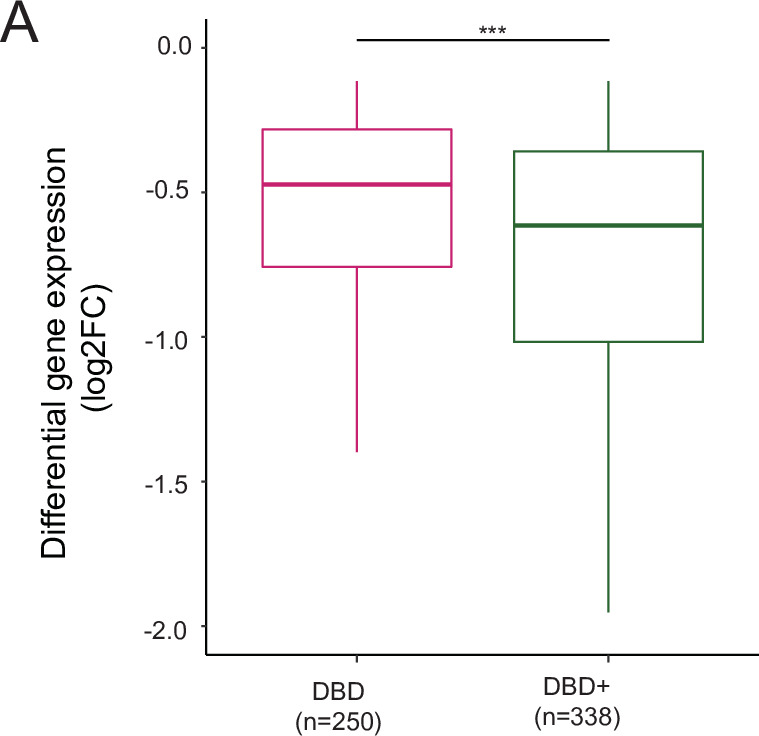
DBD-α4 helix of FLI1 induces stronger gene deactivation at DBD+ super-enhancer compared to DBD super -enhancers in A-673 cells. (**A**) Expression level of downregulated genes at DBD and DBD+ super-enhancers in A-673 cells. p-Value<0.001. Boxplots depict the minimum, first quartile, median, third quartile, and maximum.

**Figure 4—figure supplement 2.**
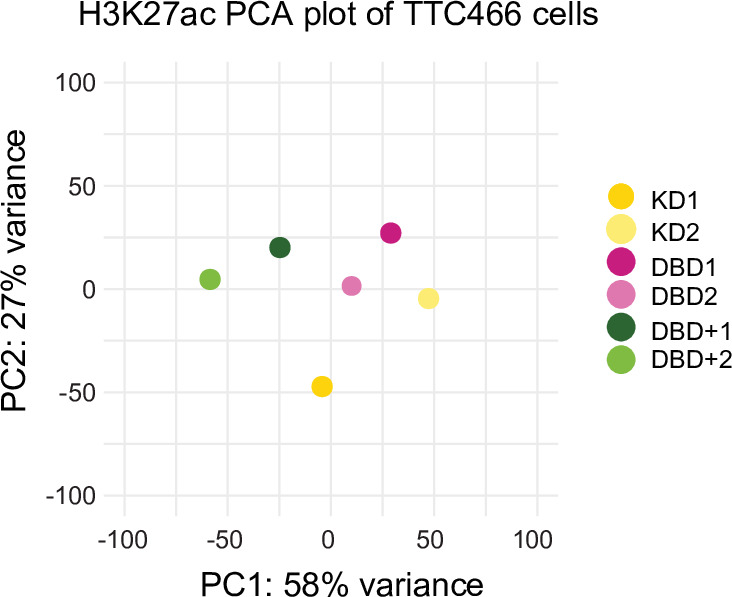
H3K27ac CUT&Tag analysis in TTC-466 cells. Principal component analysis (PCA) plot of H3K27ac peaks in biological replicates of KD, DBD, and DBD+.

Transcription factors have been shown to establish super-enhancers at key genes that drive cellular identity (Whyte et al., 2013). We therefore sought to understand whether the DBD-α4 helix participates in the formation of super-enhancers. We utilized the ROSE algorithm to define enhancers and super-enhancers in DBD and DBD+ cells (Lovén et al., 2013; Whyte et al., 2013). We found 9480 and 10,370 typical enhancers in DBD and DBD+, respectively (Figure 4D). We also identified 707 and 454 super-enhancers in DBD and DBD+, respectively. Although the super-enhancers associated with DBD+ were fewer in number compared to DBD, these super-enhancers were larger in size (Figure 4E) and contained more H3K27ac peaks (Figure 4D). We also assessed the expression level of genes associated with super-enhancers and found DBD+ super-enhancer genes had more gene activation (Figure 4F) and deactivation (Figure 4—figure supplement 1A). These data suggest the DBD-α4 helix is required for the formation of enhancers and super-enhancers and is therefore important for the regulation of genes in these highly clustered regions of the genome.

### The DBD-α4 helix promotes binding at longer and denser GGAA microsatellites in A-673 cells

**Figure 5.**
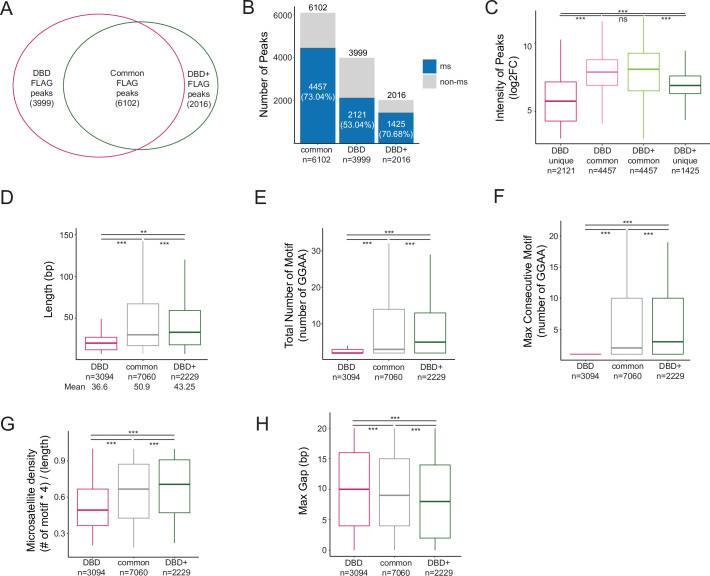
DBD-α4 helix of FLI1 promotes binding at longer and denser GGAA microsatellites in A-673 cells. (**A**) Venn diagram of overlap between FLAG peaks (FDR<0.05, FC>8, counts>80, IDR<0.01) of DBD and DBD+ cells. (**B**) Percentage of FLAG peaks bound at microsatellites in common, DBD unique, and DBD+ unique peaks. (**C**) Intensity of peaks in DBD unique (mean = 5.75), DBD common (mean = 7.76), DBD+ common (mean = 7.75), and DBD+ unique (mean = 6.83) FLAG peaks. (**D**) Length (in bp) of GGAA microsatellites bound by DBD unique (mean = 36.61), common in both (mean = 50.94), and DBD+ unique (mean = 43.25) FLAG peaks. (**E**) Total number of GGAA motifs in microsatellites bound by DBD unique (mean = 4.66), common in both (mean = 8.70), and DBD+ unique (mean = 7.78) FLAG peaks. (**F**) Maximum consecutive number of GGAA motifs in microsatellites bound by DBD unique (mean = 1.48), common in both (mean = 5.21), and DBD+ unique (mean = 5.32) FLAG peaks. (**G**) Percentage of GGAA motif in the microsatellites calculated as (# of motif × 4)/(length of microsatellites) bound by DBD unique (mean = 0.54), common in both (mean = 0.65), and DBD+ unique (mean = 0.69) FLAG peaks. (**H**) Maximum number of insertion (gaps in bp) in microsatellites bounds by DBD unique (mean = 10.1), common (mean = 9.1), and DBD+ unique (mean = 8.3) FLAG peaks. Boxplots depict the minimum, first quartile, median, third quartile, and maximum. p-Value<0.05, p-value<0.01, and p-value<0.001.

The results of TAD, loop, and enhancer analysis hinted toward a mechanism of the DBD-α4 helix in binding GGAA microsatellites that differed from our prior CUT&RUN analysis, which showed no difference in binding (Boone et al., 2021). We decided to further investigate binding patterns of DBD and DBD+ FLAG peaks genome-wide from our A-673 CUT&Tag dataset generated for this study. We found 8118 DBD+ and 10,101 DBD FLAG peaks genome-wide that passed the threshold of FDR<0.05, FC>8, counts>80, IDR<0.01. When we overlapped the two sets of FLAG peaks, we found 6102 common, 3999 DBD unique, and 2016 DBD+ unique FLAG peaks (Figure 5A). When each group of FLAG peaks was overlapped with genomic GGAA microsatellites, we found that 70.68% of DBD+ unique and 73.04% of common peaks bind at GGAA microsatellites (Figure 5B). When we considered DBD unique peaks, we found GGAA microsatellites binding shifted down to 53.04% (Figure 5B, DBD bar). Common FLAG peaks were found to have the highest peak intensity, followed by DBD+ peaks, then DBD peaks (Figure 5C). Taken together, these data demonstrate a role of the DBD-α4 helix in binding to a subset of GGAA microsatellites.

**Figure 5—figure supplement 1.**
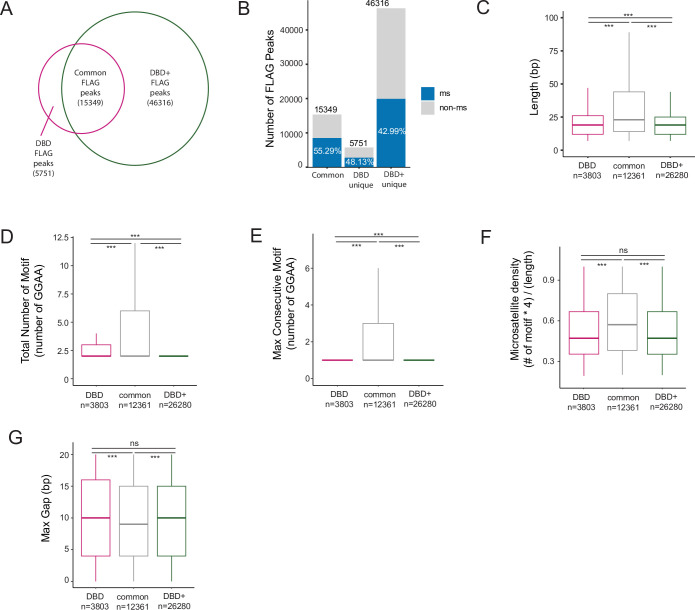
FLAG CUT&Tag analysis in TTC-466 cells. (**A**) Venn diagram of overlap between FLAG peaks (FDR<0.05, FC>8, counts>80, IDR<0.01) of DBD and DBD+ cells. (**B**) Percentage of FLAG peaks bound at microsatellites in common, DBD unique, and DBD+ unique peaks. (**C**) Length (in bp) of GGAA microsatellites bound by DBD unique (mean = 30.28), common in both (mean = 37.79), and DBD+ unique (mean = 22.74) FLAG peaks. (**D**) Total number of GGAA motifs in microsatellites bound by DBD unique (mean = 3.70), common in both (mean = 6.05), and DBD+ unique (mean = 3.00) FLAG peaks. (**E**) Maximum consecutive number of GGAA motifs in microsatellites bound by DBD unique (mean = 1.35), common in both (mean = 3.44), and DBD+ unique (mean = 1.57v) FLAG peaks. (**F**) Percentage of GGAA motif in the microsatellites calculated as (# of motif × 4)/(length of microsatellites) bound by DBD unique (mean = 0.53), common in both (mean = 0.60), and DBD+ unique (mean = 0.54) FLAG peaks. (**G**) Maximum number of insertion (gaps in bp) in microsatellites bound by DBD unique (mean = 10.0), common (mean = 9.36), and DBD+ unique (mean = 9.77) FLAG peaks. Boxplots depict the minimum, first quartile, median, third quartile, and maximum. p-Value<0.05, p-value<0.01, and p-value<0.001.

We therefore further characterized the GGAA microsatellites bound by these three sets of peaks: DBD unique, common, and DBD+ unique peaks (Figure 5D–H). We assessed the length of microsatellites and found uniquely DBD bound GGAA microsatellites to be shorter (mean = 36.61 bp) than uniquely DBD+ bound GGAA microsatellites (43.25 bp) (Figure 5D). GGAA microsatellites bound by both DBD and DBD+ were the longest (50.94 bp) (Figure 5D). When we quantified the total number of GGAA repeats, we found that the mean number of GGAA motifs in uniquely DBD bound GGAA microsatellites was 4.66 compared to 7.78 in uniquely DBD+ bound GGAA microsatellites and 8.70 in GGAA microsatellites commonly bound in both conditions that were significantly different from each other (Figure 5E). Next, we quantified the maximum consecutive GGAA repeats within a microsatellite and found that the DBD unique peaks contained GGAA repeats no longer than two repeats (Figure 5F), whereas common and DBD+ unique peaks were localized at approximately five consecutive GGAA repeats (mean = 5.21 and 5.32 for common and DBD+, respectively, Figure 5F). Next, we plotted GGAA motif density and observed that DBD unique microsatellites are on average (mean = 0.54) less dense compared to common (0.65) and DBD+ (0.69) bound GGAA microsatellites (Figure 5G). Inverse of GGAA density is the max gap or number of non-GGAA bp in the microsatellites. We then assessed the gap size in each of the groups and found DBD+ binds microsatellites that contain the least amount of gaps (mean = 8.3), followed by common (9.1), then DBD unique peak microsatellites (10.1) (Figure 5H). These analyses demonstrate that the DBD-α4 helix is required for effective binding at GGAA microsatellites that are medium in length and denser in its GGAA motifs.

### The DBD-α4 helix is necessary for productive transcription hub formation at GGAA microsatellites in A-673 cells

Transcription is a process that is regulated across large genomic distances, time, and space (Cramer, 2019; Lee and Young, 2013). One of the emerging concepts that unifies observations of such a regulation is the concept of transcriptional hubs. Transcriptional hubs are actively transcribed regions containing clusters of transcription factors and RNA Pol II, and they are highly characterized by enhancers (Lim and Levine, 2021). EWSR1::FLI1 has been shown to form such hubs via the intrinsically disordered region of EWSR1 at GGAA microsatellites (Chong et al., 2018). Since the DBD-α4 helix is required in effective binding at GGAA microsatellites and the downstream regulation of 3D chromatin, we sought to understand if the DBD-α4 helix participates in the function of EWSR1::FLI1 in the formation of transcriptional hubs.

**Figure 6.**
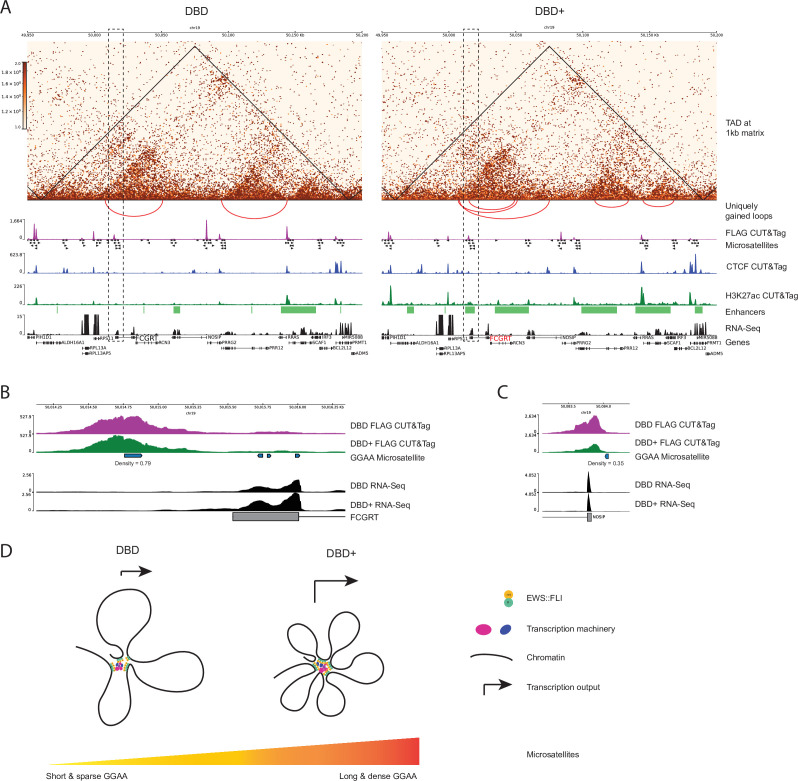
The DBD-α4 helix promotes formation of transcription hubs by effective binding at microsatellites. (**A**) 250 kb region on chr 19 containing FCGRT and other genes. Topologically associated domains (TADs) are depicted on 1 kb matrices (DBD/KD and DBD+/KD). Uniquely gained loops are shown as red inverted arcs. FLAG CUT&Tag bigwig tracks depicted in magenta. GGAA microsatellites in hg19. CTCF CUT&Tag track is in blue middle row. H3K27ac tracks are in green. Enhancers and super-enhancers are shown as green bars. Gene expression is in black tracks. (**B**) FCGRT promoter region containing GGAA microsatellites. (**C**) NOSIP promoter region containing GGAA repeats. (D) Model of EWSR1::FLI1-driven transcription hub.

To characterize EWSR1::FLI1-driven transcription hubs, we turned to a region containing the *FCGRT* gene in A-673 cells since EWSR1::FLI1 at this locus is shown to form a hub at GGAA repeats (Figure 6A, Gangwal et al., 2008; Chong et al., 2018). The *FCGRT* gene resides in a 250 kb TAD (top panel) in both DBD and DBD+ cells. Considering that TADs are highly self-interacting regions and that interactions across different TADs are limited by insulator elements (Dixon et al., 2012; Hou et al., 2012; Nora et al., 2012), we used TADs as proxy for hubs as we did not expect hubs to extend beyond TAD boundaries. Within the hub, we observed instances where DBD and DBD+ clusters appear in similar locations. In DBD cells, however, we noticed increases in the intensity of all peaks except the *FCGRT* promoter GGAA microsatellite peak (Figure 6A, magenta tracks). When we zoomed in on this microsatellite, DBD and DBD+ peaks had a similar binding pattern (Figure 6B, magenta and green tracks). Despite this similarity, DBD binding did not facilitate the formation of chromatin loops at the *FCGRT* promoter as efficiently as DBD+ (inverted red arcs). Furthermore, this lack of loop formation correlated with decreased enhancer marks (Figure 6A, green tracks and bar) and decreased expression of the *FCGRT* gene (black tracks, padj<0.05, FC>2). Even though binding at the *FCGRT* microsatellite is comparable between DBD and DBD+, DBD is less able to modulate transcription and chromatin at this microsatellite with high GGAA motif density (0.79, Figure 6B).

**Figure 6—figure supplement 1.**
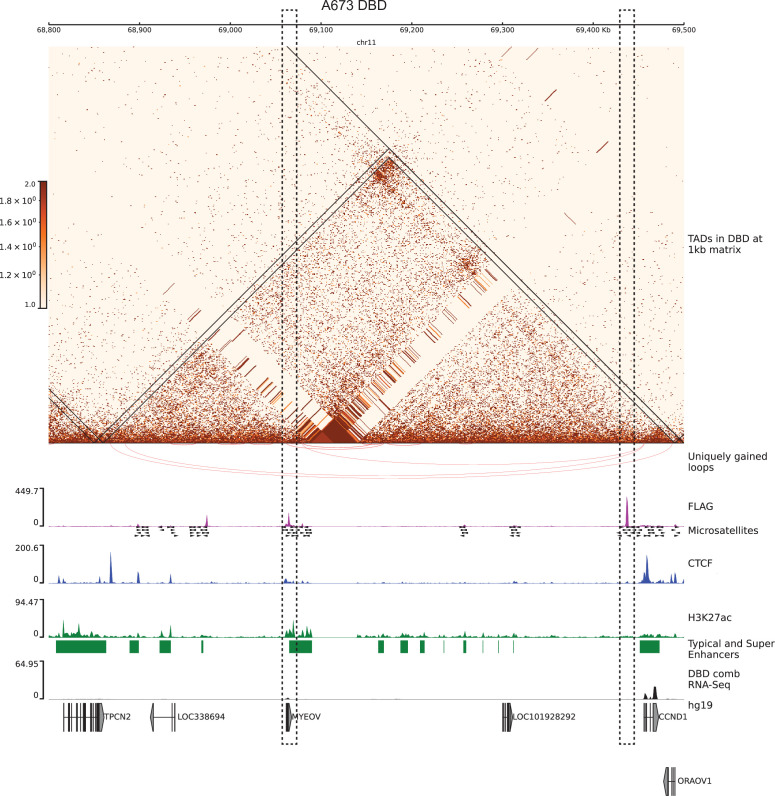
*CCND1* hub in 700 kb region on chr 11 in A-673 DBD cells. Topologically associated domains (TADs) are depicted on 1 kb matrices (DBD/KD). Uniquely gained loops are shown as red inverted arcs. FLAG CUT&Tag bigwig tracks depicted in magenta. GGAA microsatellites in hg19. CTCF CUT&Tag track is in blue middle row. H3K27ac tracks are in green. Enhancers and super-enhancers are shown as green bars. Gene expression is in black tracks.

**Figure 6—figure supplement 2.**
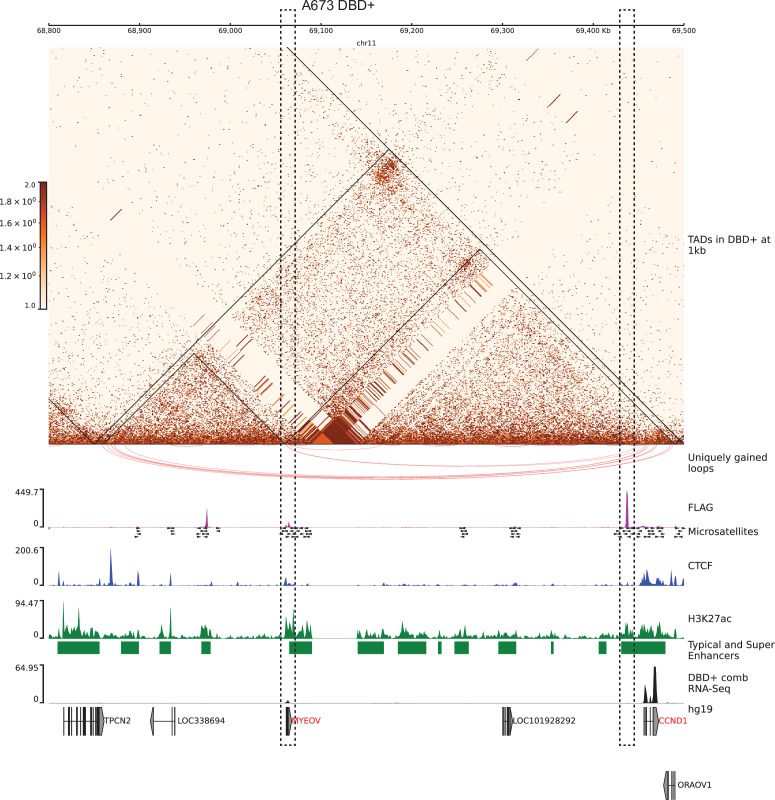
*CCND1* hub in 700 kb region on chr 11 in A-673 DBD+ cells. Topologically associated domains (TADs) are depicted on 1 kb matrices (DBD+/KD). Uniquely gained loops are shown as red inverted arcs. FLAG CUT&Tag bigwig tracks depicted in magenta. GGAA microsatellites in hg19. CTCF CUT&Tag track is in blue middle row. H3K27ac tracks are in green. Enhancers and super-enhancers are shown as green bars. Gene expression is in black tracks.

**Figure 6—figure supplement 3.**
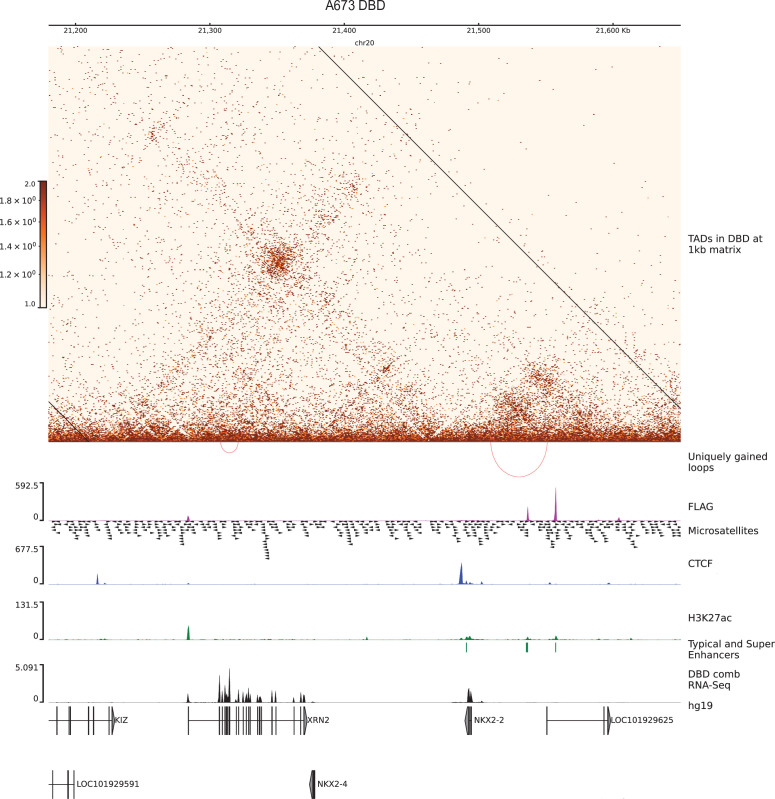
*NKX2-2* hub on chr 20 in A-673 DBD cells. Topologically associated domains (TADs) are depicted on 1 kb matrices (DBD/KD). Uniquely gained loops are shown as red inverted arcs. FLAG CUT&Tag bigwig tracks depicted in magenta. GGAA microsatellites in hg19. CTCF CUT&Tag track is in blue middle row. H3K27ac tracks are in green. Enhancers and super-enhancers are shown as green bars. Gene expression is in black tracks.

**Figure 6—figure supplement 4.**
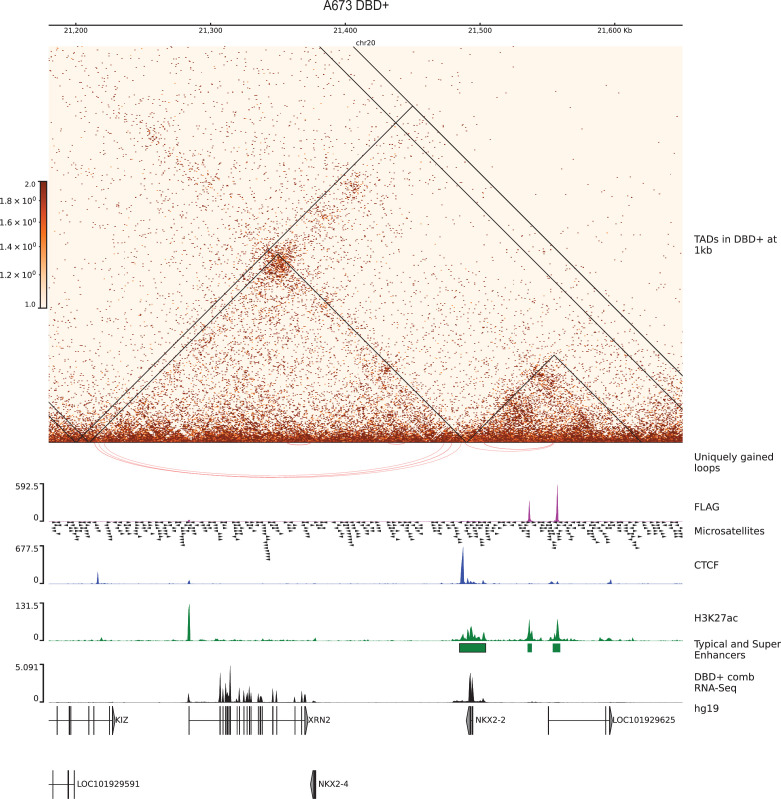
*NKX2-2* hub on chr 20 in A-673 DBD+ cells. Topologically associated domains (TADs) are depicted on 1 kb matrices (DBD+/KD). Uniquely gained loops are shown as red inverted arcs. FLAG CUT&Tag bigwig tracks depicted in magenta. GGAA microsatellites in hg19. CTCF CUT&Tag track is in blue middle row. H3K27ac tracks are in green. Enhancers and super-enhancers are shown as green bars. Gene expression is in black tracks.

**Figure 6—figure supplement 5.**
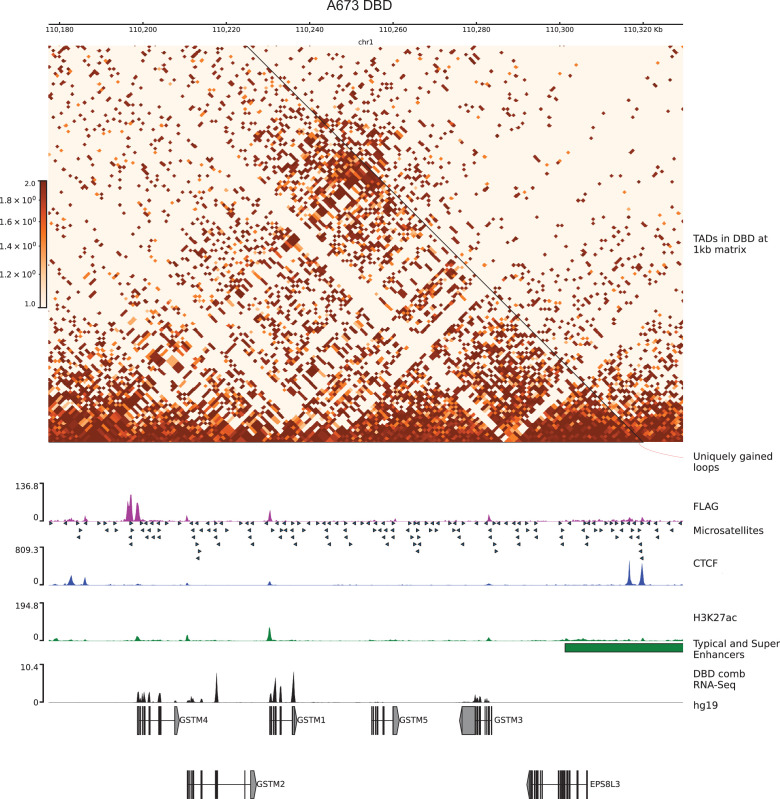
*GSTM4* hub chr 1 in A-673 DBD cells. Topologically associated domains (TADs) are depicted on 1 kb matrices (DBD/KD). Uniquely gained loops are shown as red inverted arcs. FLAG CUT&Tag bigwig tracks depicted in magenta. GGAA microsatellites in hg19. CTCF CUT&Tag track is in blue middle row. H3K27ac tracks are in green. Enhancers and super-enhancers are shown as green bars. Gene expression is in black tracks.

**Figure 6—figure supplement 6.**
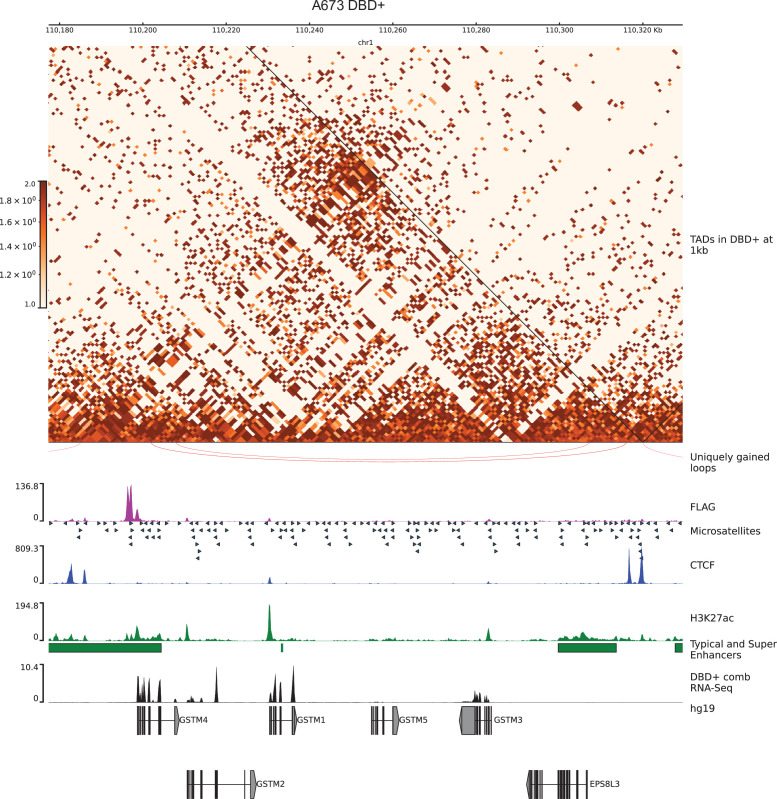
*GSTM4* hub chr 1 in A-673 DBD+ cells. Topologically associated domains (TADs) are depicted on 1 kb matrices (DBD+/KD). Uniquely gained loops are shown as red inverted arcs. FLAG CUT&Tag bigwig tracks depicted in magenta. GGAA microsatellites in hg19. CTCF CUT&Tag track is in blue middle row. H3K27ac tracks are in green. Enhancers and super-enhancers are shown as green bars. Gene expression is in black tracks.

**Figure 6—figure supplement 7.**
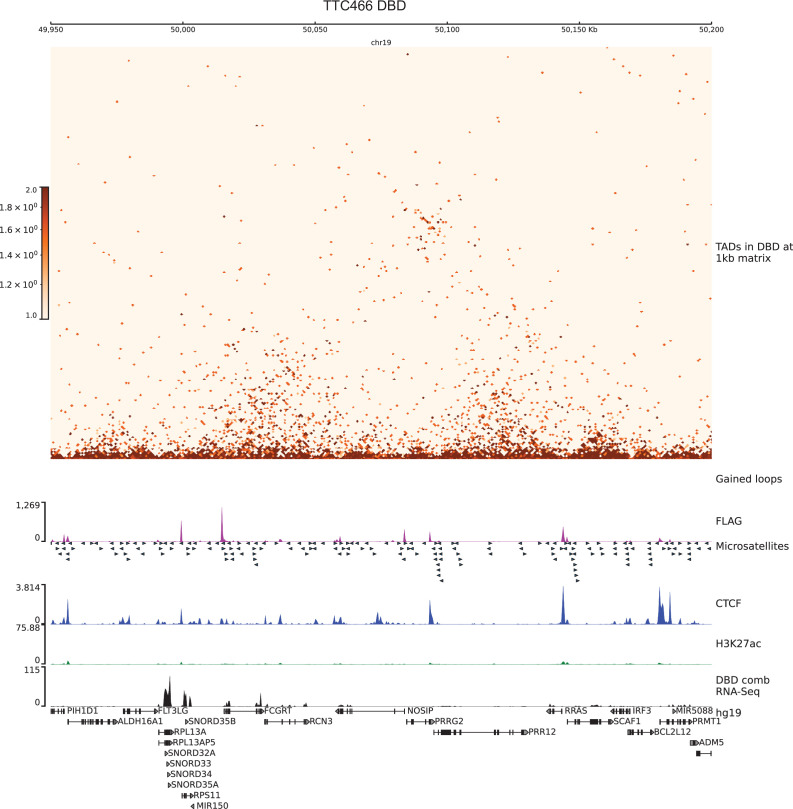
FCGRT hub in 250 kb region on chr 19 in TTC-466 DBD cells. Topologically associated domains (TADs) are depicted on 1 kb matrices (DBD/KD). Gained loops are shown as red inverted arcs. FLAG CUT&Tag bigwig tracks depicted in magenta. GGAA microsatellites in hg19. CTCF CUT&Tag track is in blue middle row. H3K27ac tracks are in green. Gene expression is in black tracks.

**Figure 6—figure supplement 8.**
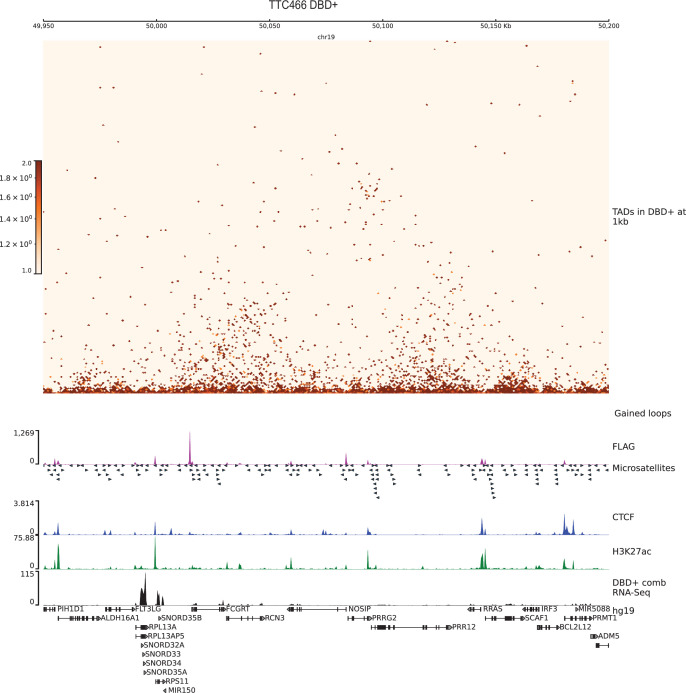
*FCGRT* hub in 250 kb region on chr 19 in TTC-466 DBD+ cells. Topologically associated domains (TADs) are depicted on 1 kb matrices (DBD+/KD). Gained loops are shown as red inverted arcs. FLAG CUT&Tag bigwig tracks depicted in magenta. GGAA microsatellites in hg19. CTCF CUT&Tag track is in blue middle row. H3K27ac tracks are in green. Gene expression is in black tracks.

**Figure 6—figure supplement 9.**
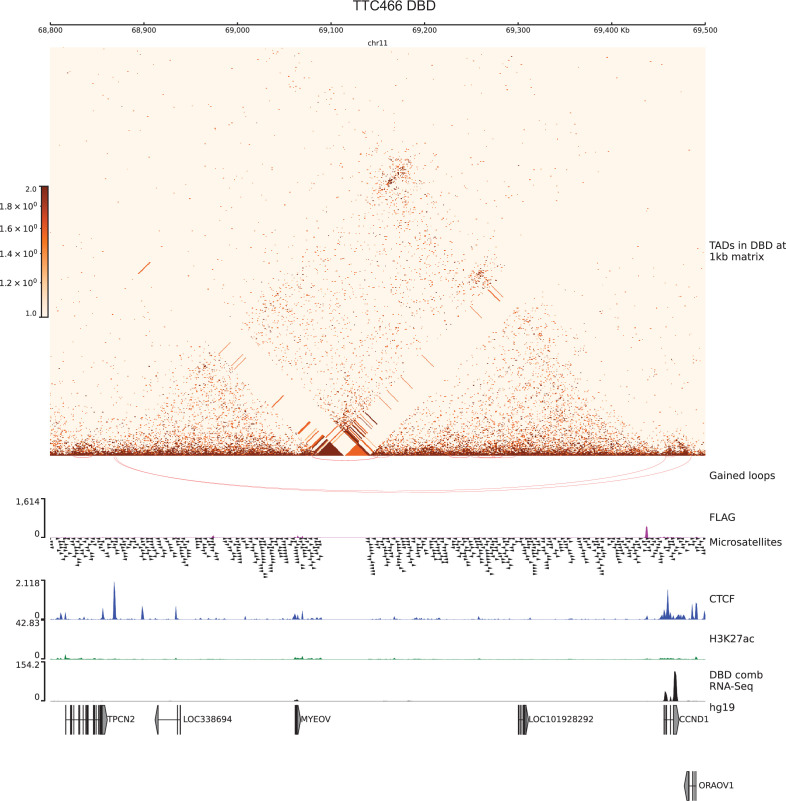
*CCND1* hub in 700 kb region on chr 11 in TTC-466 DBD cells. Topologically associated domains (TADs) are depicted on 1 kb matrices (DBD/KD). Gained loops are shown as red inverted arcs. FLAG CUT&Tag bigwig tracks depicted in magenta. GGAA microsatellites in hg19. CTCF CUT&Tag track is in blue middle row. H3K27ac tracks are in green. Gene expression is in black tracks.

**Figure 6—figure supplement 10.**
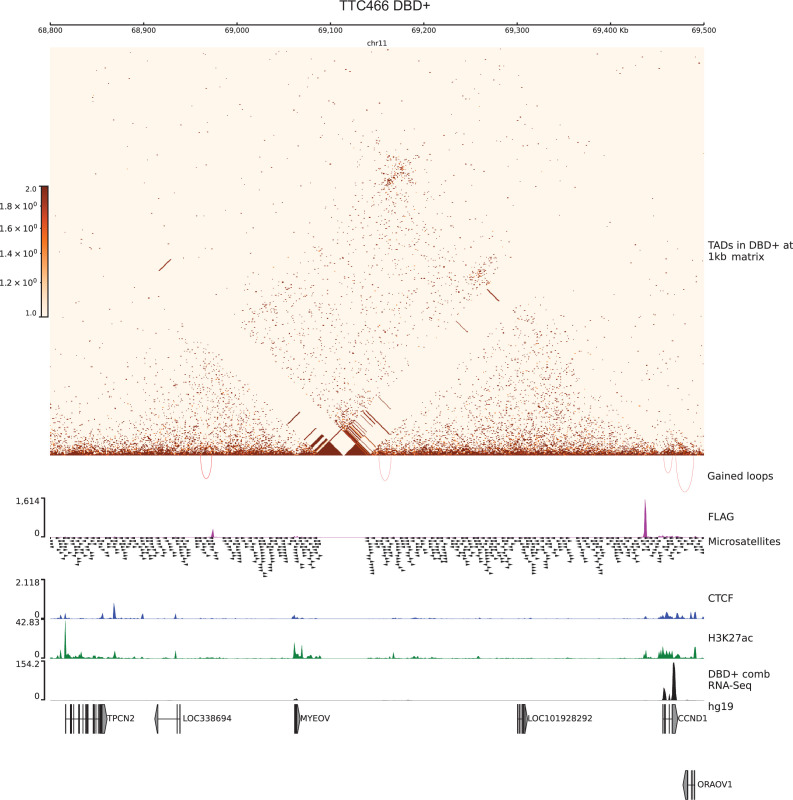
*CCND1* hub in 700 kb region on chr 11 in TTC-466 DBD+ cells. Topologically associated domains (TADs) are depicted on 1 kb matrices (DBD+/KD). Gained loops are shown as red inverted arcs. FLAG CUT&Tag bigwig tracks depicted in magenta. GGAA microsatellites in hg19. CTCF CUT&Tag track is in blue middle row. H3K27ac tracks are in green. Gene expression is in black tracks.

**Figure 6—figure supplement 11.**
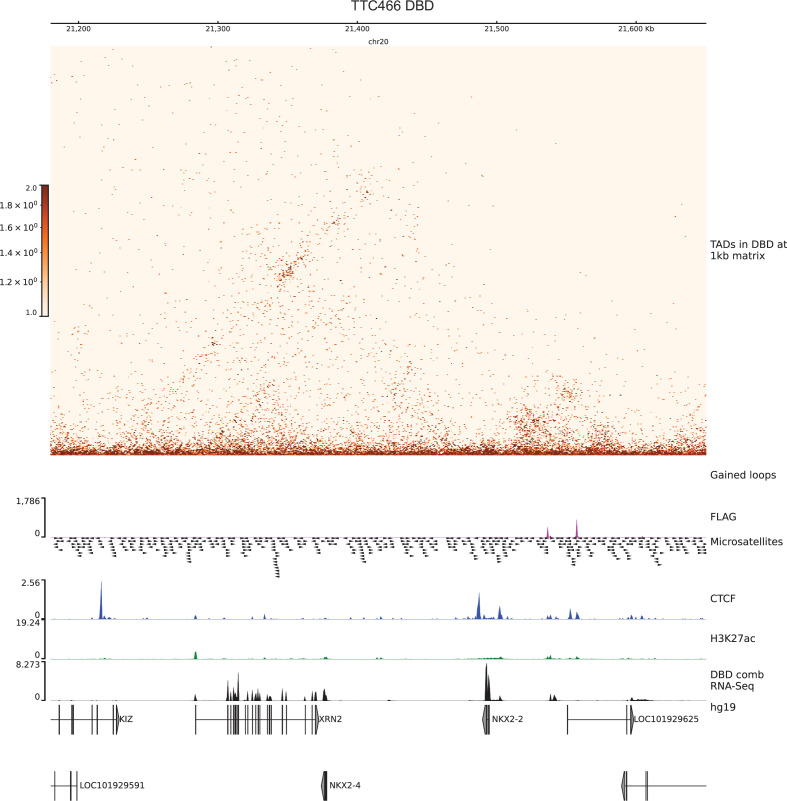
*NKX2-2* hub chr 20 in TTC-466 DBD cells. Topologically associated domains (TADs) are depicted on 1 kb matrices (DBD/KD). Gained loops are shown as red inverted arcs. FLAG CUT&Tag bigwig tracks depicted in magenta. GGAA microsatellites in hg19. CTCF CUT&Tag track is in blue middle row. H3K27ac tracks are in green. Gene expression is in black tracks.

**Figure 6—figure supplement 12.**
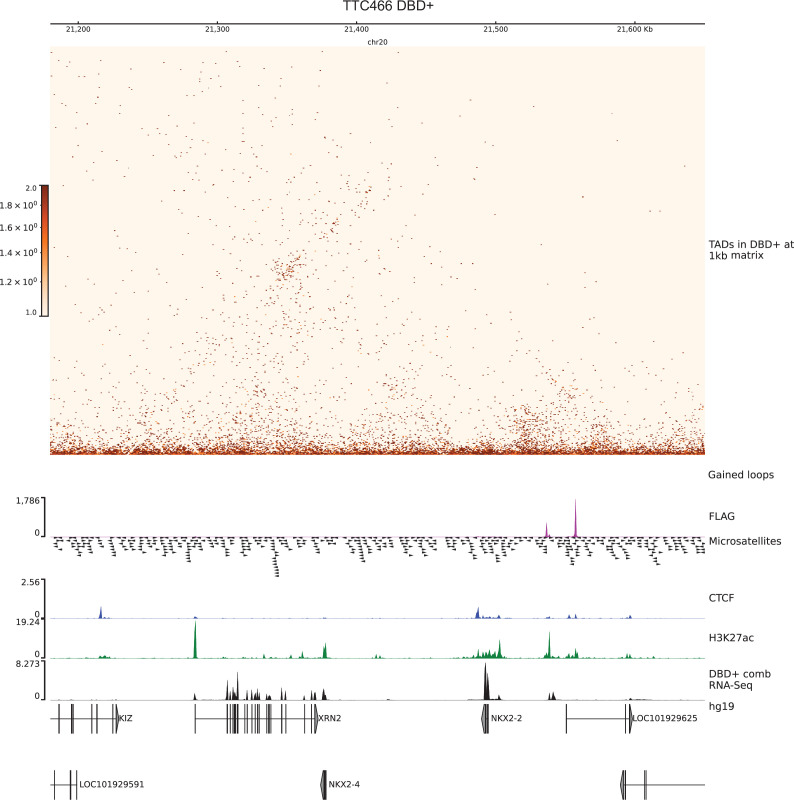
*NKX2-2* hub on chr 20 in TTC-466 DBD+ cells. Topologically associated domains (TADs) are depicted on 1 kb matrices (DBD+/KD). Gained loops are shown as red inverted arcs. FLAG CUT&Tag bigwig tracks depicted in magenta. GGAA microsatellites in hg19. CTCF CUT&Tag track is in blue middle row. H3K27ac tracks are in green. Gene expression is in black tracks.

**Figure 6—figure supplement 13.**
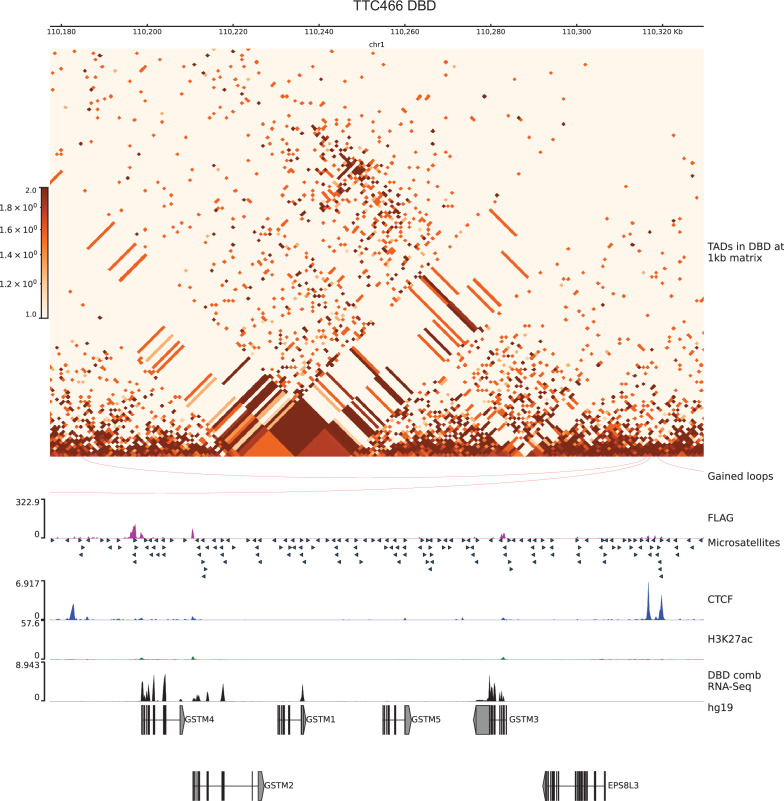
*GSTM4* hub chr 1 in TTC-466 DBD cells. Topologically associated domains (TADs) are depicted on 1 kb matrices (DBD/KD). Gained loops are shown as red inverted arcs. FLAG CUT&Tag bigwig tracks depicted in magenta. GGAA microsatellites in hg19. CTCF CUT&Tag track is in blue middle row. H3K27ac tracks are in green. Gene expression is in black tracks.

**Figure 6—figure supplement 14.**
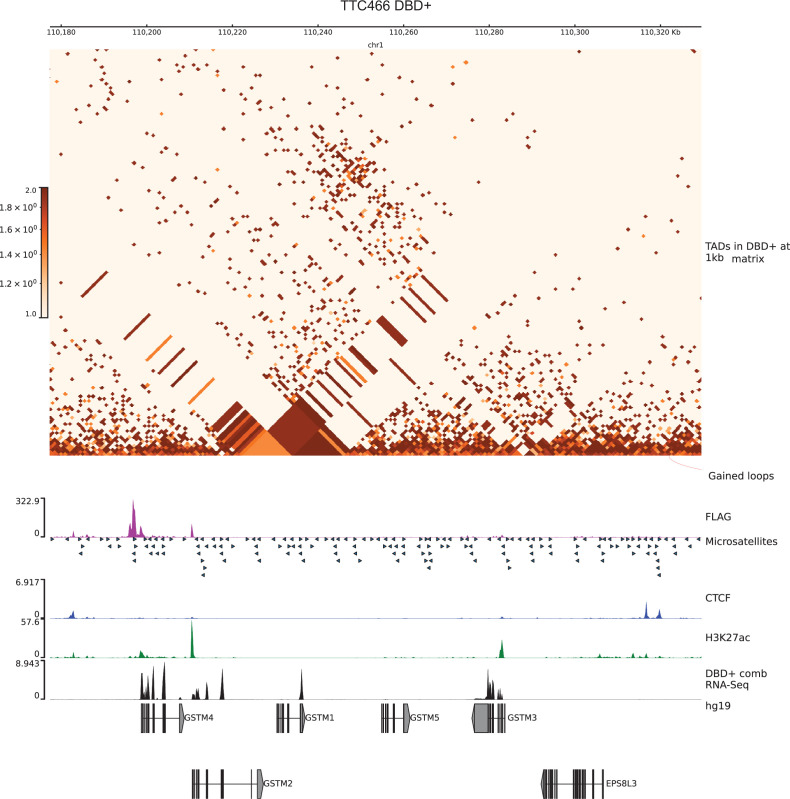
*GSTM4* hub on chr 1 in TTC-466 DBD+ cells. Topologically associated domains (TADs) are depicted on 1 kb matrices (DBD+/KD). Gained loops are shown as red inverted arcs. FLAG CUT&Tag bigwig tracks depicted in magenta. GGAA microsatellites in hg19. CTCF CUT&Tag track is in blue middle row. H3K27ac tracks are in green. Gene expression is in black tracks.

**Figure 6—figure supplement 15.**
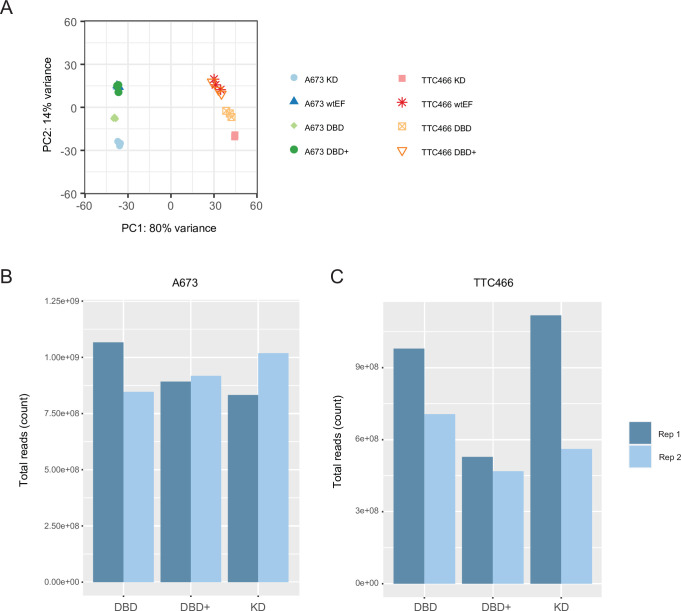
A-673 and TTC-466 cell line comparison. (**A**) Principal component analysis (PCA) plot of RNA-Seq replicates of A-673 and TTC-466 cells. (**B**) Sequencing depth of Micro-C replicates of A-673 cells. (**C**) Sequencing depth of Micro-C replicates of TTC-466 cells.

We also studied the DBD peaks that had higher intensity compared to DBD+ peaks. The majority of these peaks were at microsatellites of varying density (0.35–0.75), and two peaks at the promoters of *PRR12* and *RRAS* were not at GGAA repeats (Figure 6A). These peaks were still near GGAA microsatellites with densities of 0.53 and 0.28 for *PRR12* and *RRAS* peaks, respectively. Upon analysis of the RNA-sequencing data, we found that the expression of genes associated with this subset of peaks was not significantly differentially expressed in both DBD and DBD+ cells compared to KD (Table 1). The only gene with significantly changed expression was *FCGRT,* and this change was observed only in DBD+ cells compared to KD. Taken together, these data demonstrate that the DBD-α4 helix is required for regulating expression of the *FCGRT* gene.

**Table 1.** Differential expression of *FCGRT* hub genes in DBD and DBD+ compared to KD.

Gene symbol	DBD FC	DBD padj	DBD+ FC	DBD+ padj
*ALDH16A1*	1.207	0.046	1.107	0.329
*RPL13A*	–1.132	0.0214	–1.209	7.46E-05
*RPL13AP5*	–1.109	0.491	–1.149	0.291
*RPS11*	–1.068	0.337	–1.177	0.002
*FCGRT*	1.639	2.14E-4	2.44	2.54E-13
*RCN3*	–1.588	6.85E-06	–1.406	9.58E-4
*NOSIP*	1.065	0.447	–1.041	0.637
*PRRG2*	1.117	0.760	–1.042	0.917
*PRR12*	1.177	0.007	1.088	0.204
*RRAS*	–1.655	1.63E-17	–1.383	5.10E-08
*SCAF1*	1.065	0.357	–1.07	0.283
*IRF3*	1.081	0.402	1.196	0.0115
*BCL2L12*	1.255	0.007	1.359	6.60E-05
*PRMT1*	1.151	0.015	1.145	0.015
*ADM5*	1.122	0.740	1.064	0.864

Another locus we investigated in A-673 cells is *CCND1*, where hub formation at GGAA repeats is also reported (Riggi et al., 2014; Chong et al., 2018). The *CCND1* locus is located at the border of a 700 kb TAD in both DBD and DBD+ cells (Figure 6—figure supplement 1A and B). However, in DBD+ cells, formation of two smaller TADs nested within the larger TAD is detected with an increased number of loops (Figure 6—figure supplement 1B). DBD and DBD+ peaks cluster in similar locations across the hub. However, DBD+ binding at *MYEOV* and *CCND1* promoters were the only ones that effectively modulated chromatin at these microsatellites to promote gene expression (black tracks, padj<0.05, FC>2). The *CCND1* promoter microsatellite has a density of 1, meaning that it only consists of GGAA repeats without any gaps, whereas *MYEOV* microsatellite densities were 0.44 and 0.28. We also found similar results at two more loci with EWSR1::FLI1-bound GGAA microsatellites: *NKX2-2* and *GSTM4* (Figure 6—figure supplements 2 and 3; Luo et al., 2009; Chong et al., 2018; Showpnil et al., 2022). These findings further demonstrate the preference of DBD-α4 helix for binding at dense GGAA repeats and the increased binding intensity seen for DBD without the DBD-α4 helix at shorter and less dense GGAA microsatellites. Taken together, these data provide examples of transcriptional hubs spanning a large genomic distance containing numerous genes and show how transcriptional output at these hubs can be regulated by EWSR1::FLI1 binding at GGAA microsatellites.

### The DBD-α4 helix is required for transcription and chromatin regulation in TTC-466 cells

In order to understand if these observations from A-673 cells are applicable in other models of Ewing sarcoma, we used our conventional KD/rescue system in TTC-466 cells, which express the EWSR1::ERG fusion. We depleted endogenous EWSR1::ERG with shRNA and rescued with DBD and DBD+ constructs and tested expression of rescue constructs, transcriptional changes, and transformation in agar before performing Micro-C analysis (Figure 1—figure supplement 3A–C). Importantly, the winged helix-turn-helix domain of wild-type FLI1 is highly homologous to ERG. From the first α1 helix through the flanking α4 helix of the ETS DBD, ERG varies from FLI1 in only two residues of α1 (alanines in FLI1 that are serines in ERG, Poon and Kim, 2017). Our DBD and DBD+ constructs are thus >97% homologous with analogous constructs for ERG. These experiments largely recapitulated our results in A-673 cells (Figure 1—figure supplement 1). Notably, PCA of RNA-sequencing data showed that, as for A-673 cells, wtEF clustered away from the KD condition, with DBD+ clustering with wtEF and DBD clustering between KD and wtEF (Figure 1—figure supplements 1C and 3B). Taken together, these data suggest that the DBD-α4 helix is also required in transcription and transformation processes of Ewing sarcoma in TTC-466 cells.

Next, we sought to understand the 3D chromatin organization of TTC-466 cells expressing DBD and DBD+ proteins. First, we performed MDS analysis of the top 1000 interactions of Micro-C matrices at 500 kb resolution (Figure 1—figure supplement 4A). KD replicates clustered furthest away from DBD+ replicates. Additionally, DBD replicates fell between the KD and DBD+ replicates. We also assessed the contact frequency across the genome in combined replicates of KD, DBD, and DBD+ cells at 5 kb resolution (Figure 1—figure supplement 4B). At short distance (up to around 10^5^ bp), cells expressing DBD have more contacts in comparison to KD and DBD+ cells, while DBD+ expressing cells make more frequent contacts than KD and DBD cells at longer distances (Figure 1—figure supplement 4B). We further conducted differential interaction analysis using 500 kb matrices to compare cells expressing DBD and DBD+ replicates to KD cells using the same cutoff values as above for A-673 cells (Figure 1—figure supplement 4C and D). First, we detected 42,891 and 2598 interactions in DBD+ and DBD cells, respectively, using cutoffs of p-value<0.2 and log_2_FC>0.5. We then used more stringent criteria (adjusted p-value<0.05, log_2_FC>1.5) to identify DIRs. We found that DBD+ cells have 1742 DIRs compared to KD cells (Figure 1—figure supplement 4C), whereas DBD cells have 72 DIRs (Figure 1—figure supplement 4D). Taken together, these results suggest that the DBD-α4 helix of FLI1 participates in restructuring 3D chromatin organization in TTC-466 cells.

We then repeated the TAD and loop level analysis in DBD and DBD+ expressing TTC-466 cells with respect to the KD condition. Surprisingly, we found more TADs and loops in DBD expressing cells (16,350 TADs; 1646 loops) compared to DBD+ cells (7099 TADs; 297 loops) with comparable patterns of overlap between DBD and DBD+ in both TAD and loop analysis (Figure 2—figure supplement 2, Figure 3—figure supplement 2). Notably, despite a finding significantly more loops in the DBD condition, overlap analysis showed that the loops gained in DBD and DBD+ expression conditions are largely different while the loops lost in both conditions are similar (Figure 3—figure supplement 2B and C). This pattern was also detected in A-673 cells (Figure 3B and Figure 3—figure supplement 1), suggesting that common mechanisms drive altered chromatin looping in both cell lines.

We next used CUT&Tag for H3K27ac and FLAG to assess the enhancer landscape and construct binding in DBD and DBD+ expressing TTC-466 cells (Figure 4—figure supplement 2, Figure 5—figure supplement 1). Similar to A-673 cells (Figure 4A) and reflecting the transcriptional changes seen in RNA-sequencing (Figure 1—figure supplement 3B), we found the H3K27ac signal for DBD samples clustered between DBD+ and KD conditions in TTC-466 cells (Figure 4—figure supplement 2). In contrast to A-673 cells, we found many more FLAG peaks in DBD+ expressing TTC-466 cells (61,665) compared to DBD expressing cells (21,000), with most DBD-bound loci commonly also bound by DBD+ (Figure 5—figure supplement 1A). We further found that 55.29% of common peaks overlapped GGAA microsatellites, while 48.13% and 42.99% unique DBD and DBD+ peaks, respectively, overlapped GGAA microsatellites (Figure 5—figure supplement 1B). We then characterized the GGAA microsatellites uniquely bound by either DBD, DBD+, or both (Figure 5—figure supplement 1C–G). We found the common FLAG peaks were overlapped with the longest set of GGAA repeats (mean = 37.79 bp) compared to unique DBD peaks (mean = 30.28 bp) and DBD+ unique peaks (mean = 22.74 bp), and this led to common peaks having the longest and densest GGAA microsatellites in TTC-466 cells (Figure 5—figure supplement 1C–G).

We then sampled *FCGRT*, *CCND1*, *NKX2-2*, and *GSTM4* in TTC-466 cells (Figure 6—figure supplements 4–7, Gangwal et al., 2008; Luo et al., 2009; Showpnil et al., 2022; Riggi et al., 2014; Chong et al., 2018). At these loci in TTC-466 cells, we found only few loops in *CCND1* and *GSTM4* regions (Figure 6—figure supplements 4–7), and no TADs were detected, reflecting differences in our TAD and loop analysis between cell lines as outlined above and discussed below. However, the overall pattern of DBD and DBD+ binding was similar to that seen in A-673 cells, with DBD showing greater peak intensity at non-GGAA microsatellites and equal or lesser intensity at EWSR1::FLI1-bound GGAA microsatellites. Additionally, DBD+ expressing cells had greater H3K27ac peak intensity at these loci, also similar to that seen in A-673 cells and consistent with the rescue of transcriptional activity observed for DBD+ in both A-673 and TTC-466 cell lines. Thus, the genome-wide analyses and the Ewing-specific loci multi-omic analysis using RNA-Seq, Micro-C, H3K27ac CUT&Tag, and FLAG CUT&Tag indicate that the DBD-αhelix is required for fusion-driven oncogenic gene regulation in Ewing sarcoma.

## Discussion

We found generally concordant results between cell lines, further supporting the critical role for the DBD-α4 helix in EWSR1::FLI1-mediated changes to transcription, the enhancer landscape, and 3D chromatin structure. There were a few differences between cell lines, and this has many possible explanations, none of which are mutually exclusive. One possibility is the high level of heterogeneity found in genome regulation between Ewing sarcoma cell lines. Though there are several critical genes found to be regulated by EWSR1::FLI1 in many samples, very few genes are regulated the same way across all tested cell lines. As an example, high expression of *NKX2-2* can be used as a diagnostic marker in Ewing sarcoma and is upregulated by EWSR1::FLI1 binding at a distal GGAA microsatellite in many cell lines, including A-673 (Riggi et al., 2014; Showpnil et al., 2022). However, the chromatin architecture in TC71 cells has a unique TAD boundary insulating the *NKX2-2* gene from the EWSR1::FLI1-bound GGAA microsatellite, and *NKX2-2* is instead highly expressed through other regulatory mechanisms (Showpnil et al., 2022). To address the cell line to cell line variability, we plotted RNA-sequencing of the replicates from both cell lines on a same PCA plot (Figure 6—figure supplement 8) and found that cell line-specific gene expression comprised the majority of the variability, with each line clustering together along the first principal component. Notably, within each cell line, similar clusters were found, placing DBD+ near wtEF away from the KD condition along the second principal component, with DBD between these two clusters (Figure 6—figure supplement 8A).

Another possible reason for differences, particularly in the TAD and loop analyses, is related to the sequencing depths in both cell lines. We plotted the sequencing depth of each replicate of Micro-C data in both cell lines (Figure 6—figure supplement 8B and C). In A-673 cells, the total reads were similar between biological replicates, as well as different conditions: 900,000,000 reads (Figure 6—figure supplement 15B). For TTC-466 cells, there is more variability both between the replicates of the same condition and between conditions (Figure 6—figure supplement 15C). Specifically, the DBD+ replicates had total read of 450,000,000 (Figure 6—figure supplement 8C, middle bars). This relatively low coverage of DBD+ replicates could contribute to lower numbers of TADs and loops detected in DBD+ TTC-466 cells compared to DBD TTC-466 cells. Lastly, another notable difference was found in the microsatellite binding preferences of different constructs in different cell lines. We found many more DBD+ FLAG peaks in TTC-466 cells. This may have captured many more binding events at smaller and less dense microsatellites. As it is difficult to analytically normalize for the possibility of cell-line specific factors that influence CUT&Tag, a more reductionist biochemical approach may be needed to fully resolve differences in affinities for GGAA microsatellites of varying characteristics between DBD and DBD+. Nonetheless, at specific loci regulated by EWSR1::FLI1 binding at GGAA repeats, we found similar patterns of FLAG binding in A-673 and TTC-466. Specifically, DBD+ showed relatively higher peak intensity at long and dense GGAA microsatellites as compared to DBD. The opposite was true at the other shorter or non-GGAA microsatellite bound sites, with DBD showing a relatively higher peak intensity.

Detailed differential analysis of genomic binding patterns of DBD and DBD+ was not previously performed. In our previous study, we used CUT&RUN to identify DBD and DBD+ peaks and found 90% overlap (Boone et al., 2021). In this study, CUT&Tag showed that this overlap is only 60%. This provided us with an opportunity to discern meaningful differences in genomic localization and sequence characteristics of bound loci across three groups: DBD unique, common, and DBD+ unique peaks. Importantly, the methods utilize different enzymes for the digestion step: Micrococcal nuclease (MNase) for CUT&RUN and Tn5 transposase for CUT&Tag. Thus, a few possible factors might explain the differences in peak detection between the two techniques. MNase enzyme has cleavage preferences at sites rich in adenylate, deoxyadenylate, and thymidylate (Cuatrecasas et al., 1967). It also showed a preference for open chromatin or nucleosome-free regions (Henikoff et al., 2011; Weiner et al., 2010). Because the specific effects of EWSR1::FLI1 binding on GGAA repeats in chromatin are unknown, these loci may be particularly susceptible to excess cleavage by MNase in a way that biases CUT&RUN results. In contrast, the Tn5 transposase is especially efficient at integrating adapters into open chromatin without chromatin degradation and may, therefore, be particularly efficient at capturing binding at GGAA repeat regions. Additionally, CUT&RUN generally has lower signals, higher background, and lower yields compared to CUT&Tag (Kaya-Okur et al., 2019). We thus attribute the difference in detection of FLAG peaks in our previous and current studies to the different enzymes and their ability to recognize and access repetitive elements such as GGAA repeats.

With the current results, we favor a model whereby the DBD-α4 helix stabilizes collective binding at high-density GGAA microsatellites. Our findings underscore the specific characteristics of GGAA repeats bound by EWSR1::FLI1 to drive pathogenesis of Ewing sarcoma. At Ewing-specific gene loci such as *FCGRT* and *CCND1* (Figure 6, Figure 4—figure supplement 1), we found dense microsatellites. This finding was also recapitulated at the genome-wide level as seen in the preference of DBD+ unique peaks for denser microsatellites compared to common peaks (Figure 5G). The DBD+ unique peaks also show a preference for binding at microsatellites that are longer than the DBD unique peaks and shorter than the common peaks (Figure 5D), aligning with our previous findings that an optimal number of GGAA repeats is required for binding by EWSR1::FLI1 in Ewing sarcoma transformation (Johnson et al., 2017b).

Although our data demonstrate that the DBD-α4 helix is required in collective binding at GGAA repeats, we are still unable to decipher the exact mechanism of stabilization at such repeats with our current set of data. There are a few possible mechanisms. The DBD-α4 helix could be directly interacting with the DNA or the DNA binding domain of the adjacently bound EWSR1::FLI1 molecule. Another possibility is that the DBD-α4 helix affects its function through interactions with other epigenetic regulators or transcription machinery proteins. Alternatively, intramolecular interaction with the EWSR1 domain may stabilize DNA binding and could further promote phase condensates. We favor the last mechanism since de novo ability of EWSR1::FLI1 binding GGAA repeats depends on the presence of the EWSR1 domain (Johnson et al., 2017a). Further directed studies are needed to address these possible mechanisms.

The multi-omics approach utilized in this study provided us with an opportunity to characterize transcription hubs driven by EWSR1::FLI1 genome-wide. We have shown the clustering of EWSR1::FLI1 at GGAA microsatellites underlies the formation of local 3D features such as TADs and chromatin loops. If we use TADs as proxies for hubs, we detected thousands more of these hubs in DBD+ cells compared with DBD cells, highlighting the importance of DBD-α4 helix in binding to dense GGAA repeats and in the formation of hubs across the genome. We also observed thousands of loops at unique microsatellites for both DBD and DBD+ cells adding detail to the architecture of hubs, often represented as flower-shaped structures of many loops (Zhu et al., 2021). We also probed another aspect of hubs with our H3K27ac CUT&Tag data: the presence of enhancers and super-enhancers. We showed that the DBD-α4 helix promotes more active marks genome-wide compared to DBD, leading to the formation of enhancers and super-enhancers. Finally, we presented select loci as specific examples of productive transcription hubs driven by EWSR1::FLI1 binding at dense GGAA microsatellites. Our study thus suggests a surprising role for FLI1 DBD in the process of hub formation, which is usually attributed to the EWSR1 low-complexity domain.

Because EWSR1::FLI1 is the sole driver mutation in Ewing sarcoma tumors, it is an attractive therapeutic target. However, many approaches to target EWSR1::FLI1 have been hampered by common issues that arise with targeting transcription factors, such as its location in the nucleus, its abundance in cells, and its lack of an enzymatic pocket to design a small molecule tailored to target it (Knott et al., 2019). We propose that the DBD-α4 helix is a promising therapeutic target as it doesn’t directly bind the major groove of the DNA. Additionally, because the DBD-α4 is a helix, it provides a structured region for designing small molecules to disrupt its interaction, unlike the EWSR1 low-complexity domain. Moreover, its importance in maintaining effective binding at GGAA microsatellites offers an opportunity to target EWSR1::FLI1 at the most mechanistically important sites in the pathogenesis of Ewing sarcoma. Further studies are needed to clarify the mechanism by which the DBD-α4 helix promotes effective binding at GGAA microsatellites and regulates transcription hub formation.

## Materials and methods

### Constructs and retroviruses

Mammalian expression constructs used include: Retroviral vectors encoding shRNA for luciferase-RNAi, EWSR1::FLI1-RNAi, and EWSR1::ERG-RNAi, as well as cDNA-containing vectors encoding 3xFLAG-EF, 3xFLAG-EF DBD, 3xFLAG-EF DBD+ (Boone et al., 2021). The EWSR1::FLI1 DBD and EWSR1::FLI1 DBD+ were ordered as gene blocks (Integrated DNA Technologies) and cloned into the pMSCV-hygro plasmid between BamHI and AgeI restriction sites.

### Cell culture methods

HEK293-EBNA cells were grown at 37°C, 5% CO_2_ in Dulbecco’s modified Eagle’s medium (DMEM, Corning Cellgro 10-013-CV), with 10% heat-inactivated fetal bovine serum (Gibco 16000-044), 1% penicillin/streptomycin/glutamine (P/S/Q, Gibco 10378-016), and 0.3 mg/ml Geneticin (Gibco 10131-027). A-673 was obtained from American Type Culture Collection (ATCC, Manassas, VA, USA). These cells were grown at 37°C, 5% CO_2_ in DMEM with 10% fetal bovine serum, 1% P/S/Q, and 1% sodium pyruvate (Gibco 11360-070). TTC-466 cells were obtained from Timothy Triche, MD, PhD (CHLA) and were grown at 37°C, 5% CO_2_ in RPMI with 10% fetal bovine serum, and 1% P/S/Q. For knockdown of endogenous EWSR1::FLI1 in A-673 and EWSR1::ERG in TTC-466, cells were infected with RNAi virus and subsequently infected with the cDNA-containing virus to rescue the cells. After 48 hr, cells were selected with puromycin (100 µg/µl) and hygromycin (150 µg/µl), and allowed to grow for 7–8 days prior to collection for downstream analysis. Cells were tested regularly for Mycoplasma using the PCR-based Universal Mycoplasma Detection Kit (ATCC, 30-1012K). Cell line identities were confirmed by short tandem repeat profiling (Genetica LabCorp, USA), last performed in February 2022.

### Transfection, virus production, and transduction

For the generation of retroviruses, HEK293-EBNA cells were co-transfected with retroviral expression plasmids, vesicular stomatitis virus G glycoprotein (VSV-G), and gag/pol packaging plasmids. Briefly, 2.5×10^6^ HEK293-EBNA cells were seeded in a 10 cm tissue culture dish the day before transfection, resulting in 60–70% confluency the day of transfection. 10 µg of each plasmid (gag-pol, vsv-g, and transfer plasmid) were combined with 2 ml Opti-MEM I Reduced Serum Medium (Gibco 31985070) and 90 µl MirusBio TransIT-LT1 Transfection Reagent (Mirus MIR2306) and incubated at room temperature for 20 min. The transfection mix was then added dropwise to the cells in 3 ml culture medium. Virus-containing supernatant was collected every 4 hr on day 2 (48 hr) and 3 (72 hr) post-transfection, pooled, filtered, and stored at –80°C. Ewing sarcoma cells were transduced with viral supernatants using polybrene (8 µg/ml), followed by selection with appropriate antibiotics at 48 hr post-infection. In the case of knockdown/rescues, cells were selected with 0.5–2 µg/ml puromycin and 50–150 µg/ml hygromycin B (Thermo 10687010) for 7–10 days.

### Immunodetection

Whole-cell protein extraction was completed using Pierce RIPA buffer (Thermo Fisher 88901) supplemented with Protease Inhibitor Cocktail (Sigma P8340-5ML) on ice for 30 min. For nuclear extracts, cell pellets were resuspended in LB1 buffer (50 mM Tris-HCl pH 7.5, 20 mM NaCl, 1 mM EDTA, 0.5% NP-40, 0.25%, Triton-X 100, 10% glycerol, 1 mM DTT, and Protease Inhibitor Cocktail) and incubated on a nutator for 10 min at 4°C. After a 5 min spin at 400×*g* and 4°C, the pellets were washed with LB2 buffer (10 mM Tris-HCl pH 7.5, 20 mM NaCl, 1 mM EDTA, 0.5 mM EGTA, 1 mM DTT, and Protease Inhibitor Cocktail) and centrifuged again for 5 min at 400×*g* and 4°C. The nuclear pellets were resuspended in a small volume of RIPA buffer, incubated on ice for 30 min, followed by a 30 min centrifugation step at maximum speed and 4°C.

Protein concentration was determined using the Pierce BCA Protein Assay Kit (Thermo Scientific 23225). 15–35 µg of protein samples were run on precast 4–15% gradient gels (Bio-Rad) and transferred to nitrocellulose membranes (Invitrogen). 4–15% Mini-PROTEAN TGX precast protein gel (Bio-Rad 4561084) and resolved at 120 V. Proteins were transferred to a nitrocellulose membrane (Thermo IB23002) using the iBlot 2 Gel Transfer Device (Thermo IB21001). The membrane was blocked with Odyssey PBS Blocking Buffer (Li-Cor 927-40003) for 1 hr at room temperature. Immunoblotting was performed overnight at 4°C using the following primary antibodies: anti-FLI1 rabbit (Abcam ab15289, 1:1000); monoclonal anti-FLAG M2 mouse (Sigma F1804-200UG, 1:1000); anti-α-Tubulin [DM1A] mouse (Abcam ab7291, 1:2000); and anti-Lamin B1 [EPR8985(B)] rabbit (Abcam ab133741, 1:1000). The membrane was washed with TBS/0.1% Tween 20 (TBS-T) and incubated with IRDye secondary antibodies (IRDye 680LT donkey anti-rabbit IgG, IRDye 800CW goat anti-rabbit IgG (H+L), IRDye 800CW goat anti-mouse IgG1 specific secondary antibody, Li-Cor 926-68023, 926-32211, 926-32350, 1:2000) for 1 hr at room temperature. After a final wash step with TBS-T, the membrane was imaged using the Li-Cor Odyssey CLx Infrared Imaging System. ImageJ software was used to perform densitometry analysis.

### Cycloheximide chase assay

A6327 cells were treated with 50 µg/ml cycloheximide (Cayman 14126) to assess protein stability. A 50 mg/ml CHX stock solution was freshly prepared in dimethyl sulfoxide (Thermo Fisher D12345), and an equivalent volume of DMSO was added to generate time zero samples. After 0, 2, 4, 6, 18, and 24 hr, cells were harvested, then washed once in ice-cold Hank’s Balanced Salt Solution (Gibco 14025134) and pelleted at 400×*g* for 5 min at 4°C. The cell pellets were lysed on ice in NP-40 buffer (50 mM Tris-HCl, pH 8.0, 150 mM NaCl, 1% NP-40) supplemented with phenylmethylsulfonyl fluoride (Thermo Fisher 50-165-6975) for 30 min. The total lysates were clarified by centrifugation at 12,000×*g* for 15 min at 4°C. Protein concentration was determined using the Pierce BCA Protein Assay Kit (Thermo Scientific 23225). Samples were stored at −80°C until immunoblotting. Band intensities were quantified using ImageJ (NIH), and protein half-lives were calculated from signal decay curves fitted in GraphPad Prism (v10.0, GraphPad Software).

### Soft agar assays

The anchorage-independent growth capacity of Ewing sarcoma cells was assessed using soft agar assays. Cells were seeded at a density of 7500 cells in a 6 cm^3^ tissue culture dish in duplicate in 0.8% SeaPlaque GTG agarose (Lonza 50111) mixed with Iscove’s Modified Dulbecco’s medium (Gibco 12200-036) containing 20% FBS, penicillin/streptomycin/glutamine, and puromycin/hygromycin. Agars were imaged at least 14 days after seeding, and colony counts were quantified using ImageJ software (v1.51).

### RNA-sequencing experiments, data processing, and analysis

RNA-sequencing was performed on three biological replicates of KD, DBD, and DBD+. Total RNA was extracted using RNeasy Extraction Kit and submitted to the Nationwide Children’s Hospital Institute for Genomic Medicine for RNA quality measurement, library preparation, and sequencing. Briefly, cDNA libraries were prepared from total RNA with TruSeq Stranded mRNA Kit (Illumina 20020594) and sequenced on Illumina NovaSeq SP to generate 150 bp paired-end reads. We used in-house RNA-sequencing pipeline to process and analyze the data. Low-quality reads (q<10) and adapter sequences were trimmed before alignment to the hg19 genome using STAR (Dobin et al., 2013). After alignment, the reads were counted, and differential analysis performed using DESeq2 (Love et al., 2014).

### CUT&Tag experiments

CUT&Tag was performed as described in Kaya-Okur et al., 2019, with slight modifications. 250,000 cells per CUT&Tag condition were bound to BioMag Plus Concanavalin A-coated magnetic beads (Bangs Laboratories, BP531) and incubated with the primary antibody (anti-FLAG M2 mouse, Sigma F1804-200UG, 1:100) overnight at 4°C and secondary antibody (rabbit anti-mouse, Abcam ab46540, 1:100) for 1 hr at room temperature.

Adapter-loaded protein A-Tn5 fusion protein was added at a dilution of 1:250 and incubated for 1 hr at room temperature. To activate the Tn5, tagmentation buffer containing MgCl_2_ was added and samples were incubated for 1 hr at 37°C. Reactions were stopped by the addition of EDTA, and DNA was solubilized with SDS and Proteinase K for 1 hr at 50°C. Total DNA was purified using phenol/chloroform extraction followed by ethanol precipitation. CUT&Tag libraries were prepared with NEBNext HiFi 2× PCR Master Mix (NEB M0541S) and indexed primers (Buenrostro et al., 2015) using a combined Annealing/Extension step at 63°C for 10 s and 15 cycles followed by a 1.1× post-amplification AMPure XP (Beckman Coulter, A63880) bead cleanup. The fragment size distributions and concentrations of the final libraries were determined using the High Sensitivity D1000 Screen tape assay and reagents (Agilent, 5067–5584 and 5067–5585) on the Agilent 2200 TapeStation System. Libraries were pooled and sequenced (2×150 bp paired end) on the Illumina NovaSeq S1-Xp system (Nationwide Children’s Hospital Institute for Genomic Medicine).

### Micro-C experiments

Micro-C kits (Catalog #21006) purchased from Dovetail Genomics were used to prepare Micro-C libraries. For each condition, multiple aliquots of 1×10^6^ cells were harvested and frozen at –80°C for at least 30 min. Cells were thawed at room temperature and resuspended first in PBS containing 0.3 M DSG and then in 37% formaldehyde to cross-link DNA. Cells were then digested with various amounts of MNase to achieve a digestion profile with a 40–70% mononucleosome peak observed on TapeStation D5000 HS ScreenTape. Conditions to be analyzed comparatively were digested to a similar range of mononucleosome peaks (50–70%). Once desired digestion profiles achieved, the cells were lysed and the chromatin was captured with beads to perform proximity ligation. Libraries were prepared per the protocol of Micro-C kit, and each library was indexed with unique primer pairs from IDT (#10009816 and 10010147). Micro-C libraries were then shallow-sequenced at 7–8 million (2×150 bp) read pairs on Illumina NovaSeq6000, and the QC analysis pipeline provided from Dovetail Genomics was used to assess the quality of each library. Libraries that passed the QC step was then sequenced up to 300 million read pairs on NovaSeq6000.

### CUT&Tag data processing and analysis

CUT&Tag experiments were carried out for two biological replicates of CTCF and H3K27ac, and three biological replicates for FLAG tagged DBD and DBD+ constructs. An in-house pipeline was used to analyze CUT&Tag data (Boone et al., 2021; Showpnil et al., 2022). Quality control on raw sequencing reads was performed with FastQC (v0.11.4) (Andrews, 2010). Adapter sequences and/or low-quality reads were trimmed using trim_galore (0.4.4_dev) (Krueger and Galore, 2015). Reads were aligned to human (hg19) and spike-in *Escherichia coli* (Escherichia_coli_K_12_DH10B NCBI 2008-03-17) genomes using Bowtie2 (v2.3.4.3)11,12 with the following options ’–no-unal –no-mixed –no-discordant –dovetail –phred33 -q -I 10X 700’. ‘–very-sensitive’ option was added when aligning to spike-in genome (Langmead and Salzberg, 2012). SamTools (v1.9) was used to convert sam to bam with ‘-bq 10’ option (Li and Durbin, 2009). CUT&Tag reads were spike-in normalized using DESeq2’s median ratio method to eliminate bias across different samples, minimize the effect of few outliers, and appropriately account for global occupancy changes (Anders and Huber, 2010). Spike-in normalized tracks were generated and averaged across biological replicates using deepTools (Ramírez et al., 2016). Peaks were called with spike-in normalization and their corresponding IgG as controls accounting for variation between the biological replicates using MACS2 (v2.2.7.1) (Zhang et al., 2008), DiffBind (v2.14.0) (Ross-Innes et al., 2012; Stark and Brown, 2012), and DESeq2 (v1.26.0) (Love et al., 2014). All duplicate reads were kept in the analysis. Irreproducible Discovery Rate (IDR) (v2.0.3) (Li et al., 2011) was used to identify reproducible and consistent peaks across replicates. To ensure high-quality peaks that are most likely to represent biological signals, the final peak lists were generated with the following default thresholds: FDR<0.05, log_2_FC>8, mean normalized counts of signal>80, and IDR<0.01.

### Micro-C data processing and analysis

Micro-C libraries of two biological replicates of KD, DBD, and DBD+ were prepared. Sequenced libraries were processed per instructions of Dovetail Genomics. Briefly, fastq files were aligned to hg19 reference genome using BWA-MEM algorithm with options –5SP to map mates independently (Li and Durbin, 2010). Next, parse module from pairtools (Open2C) was used to find ligation junctions in Micro-C libraries with options min-mapq 40 (alignment with mapq <40 will be marked as multi) and max-inter-align-gap 30 (if the gap is 30 or smaller, ignore the map if the gap is >30, mark as ‘null’ alignment). The parsed pair is then sorted using pairtools sort, and PCR duplicates were removed with pairtools dedup. The pairtools split command was used to split the final .pairsam into .bam (then sorted with samtools sort) and .pairs files. Using Juicer Tools, .pairs files were converted into HiC contact matrices (Durand et al., 2016b). HiC matrices were then converted to mcool matrices using hic2cool (Durand et al., 2016a).

For MDS plot of individual replicates (Figure 1B), 500 kb resolution cool matrices were converted to GI interaction objects using hicConvertFormat from HiCExplorer (v3.7.2) (Wolff et al., 2020). Then, using diffHiC package (Lun and Smyth, 2015), bins with low average abundance and low absolute counts were filtered out. Filtered reads were then scaled using library size, and bin pairs that were on the diagonal line were also removed from analysis. Joint normalization of all replicates was carried out with diffHiC. Specifically, normOffsets function was used to remove trended biases with loess normalization, and then a new set of log_2_-transformed counts adjusted by the negative binomial offset were computed. Batch effects were removed using removeBatchEffect from limma package (Ritchie et al., 2015) before plotting the top 1000 interactions. For distance-decay plot (Figure 1C), individual replicates were combined. First, the combined matrices were normalized using hicNormalize function from HiCExplorer to scale the libraries to the smallest library (Wolff et al., 2020). Scaled libraries were then plotted for diagnostic plots to determine the thresholds to use in hicCorrectMatrix function for ICE normalization. ICE-corrected 5 kb matrices were then plotted with hicPlotDistVsCounts. For DIR analysis (Figure 1D–F), multiHiCcompare package was used (Stansfield et al., 2019). Briefly, individual replicates of KD, DBD, and DBD+ were loess normalized, and pairwise comparison of DBD+ to KD and DBD to KD was done using QLF (quasi-likelihood) method with batch effect correction. Volcano plots of DIRs with padj<0.05 and FC>1.5 plotted for each comparison (DBD+ and DBD versus KD).

For TAD analysis, individual replicates were combined and then scaled using the smallest library size with hicNormalize from HiCExplorer (v3.7.2) (Wolff et al., 2020). Then, matrices at 10 kb, 25 kb, 50 kb, and 100 kb resolutions were ICE-corrected with the thresholds determined from diagnostic plots. First, using hicFindTADs function, we called TADs at the previously mentioned four resolutions for DBD and DBD+ matrices. Then, hicDifferentialTAD was used to compute differential TADs by comparing the precomputed DBD and DBD+ TAD regions with the same regions of KD matrix. Differential TADs from each resolution were combined using hicMergeDomains with default -value of 5000 to account for duplicated TADs. For CUT&Tag peak annotation, peaks (FDR <0.05, log_2_FC>8, mean normalized counts of signal>80, and IDR<0.01) were overlapped with findOverlaps functions from GenomicRanges package (Lawrence et al., 2013).

For loop calling, combined replicates at 1 kb resolution matrices that were scaled and normalized in the same manner for TAD analysis were used with Mustache (v1.2.0) (Roayaei Ardakany et al., 2020). Mustache uses scale-space theory in computer vision to detect chromatin loops. For differential loops compared to KD matrix, diff_mustache.py was used to detect loops that were gained in DBD and DBD+ compared to KD and loops that were lost in DBD and DBD+ compared to KD matrix.

### Statistical analysis

When comparing means of two groups, two-sided Student’s t test was used. When comparing more than two groups, ANOVA test was performed followed by Tukey’s honest significant difference test. p-Value<0.05, p-value<0.01, and p-value<0.001.

### Code availability

All code used in the analysis of the sequencing data is compiled in Source code 1.

## Data Availability

The sequencing datasets generated and analyzed in this study are available in the Gene Expression Omnibus. For A-673 cells all datasets are accessible at GSE249578. The TTC-466 cell line datasets are available at GSE268935 (Micro-C), GSE268940 (FLAG), GSE268941 (H3K27ac), GSE268942 (CTCF), GSE268944 (RNA-Seq). The following datasets were generated: BayanjargalA TaslimC ShowpnilIA Selich-AndersonJ CrowJC LessnickSL TheisenER 2024EWS::FLI cooperatively binds at GGAA microsatellites via DBD-a4 helixNCBI Gene Expression OmnibusGSE249578 BayanjargalA TaslimC ShowpnilIA Selich-AndersonJ CrowJC LessnickSL TheisenER 2025EWS::FLI cooperatively binds at GGAA microsatellites via DBD-a4 helix in TTC-466 [Micro-C]NCBI Gene Expression OmnibusGSE268935 BayanjargalA TaslimC ShowpnilIA Selich-AndersonJ CrowJC LessnickSL TheisenER 2025EWS::FLI cooperatively binds at GGAA microsatellites via DBD-a4 helix in TTC-466 cells [FLAG_CnT]NCBI Gene Expression OmnibusGSE268940 BayanjargalA TaslimC ShowpnilIA Selich-AndersonJ CrowJC LessnickSL TheisenER 2025EWS::FLI cooperatively binds at GGAA microsatellites via DBD-a4 helix in TTC-466 [H3K27ac_CnT]NCBI Gene Expression OmnibusGSE268941 BayanjargalA TaslimC ShowpnilIA Selich-AndersonJ CrowJC LessnickSL TheisenER 2025EWS::FLI cooperatively binds at GGAA microsatellites via DBD-a4 helix in TTC-466 [CTCF_CnT]NCBI Gene Expression OmnibusGSE268942 BayanjargalA TaslimC ShowpnilIA Selich-AndersonJ CrowJC LessnickSL TheisenER 2025EWS::FLI cooperatively binds at GGAA microsatellites via DBD-a4 helix in TTC-466 [RNA-seq]NCBI Gene Expression OmnibusGSE268944

## Additional files

MDAR checklist

Source code 1. Source code for sequencing data analysis.
