# Supplementary figures and images for: The DBD-α4 helix of EWSR1::FLI1 is required for GGAA microsatellite binding that underlies genome regulation in Ewing sarcoma
Source: eLife. 2026 Jun 15;13:RP95626. doi: 10.7554/eLife.95626 (PMC13268648; doi:10.7554/eLife.95626)

A673 Rep 1

FLI

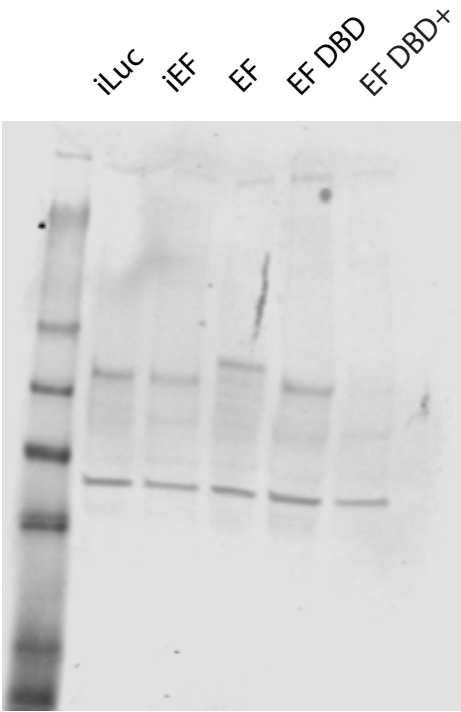

FLAG

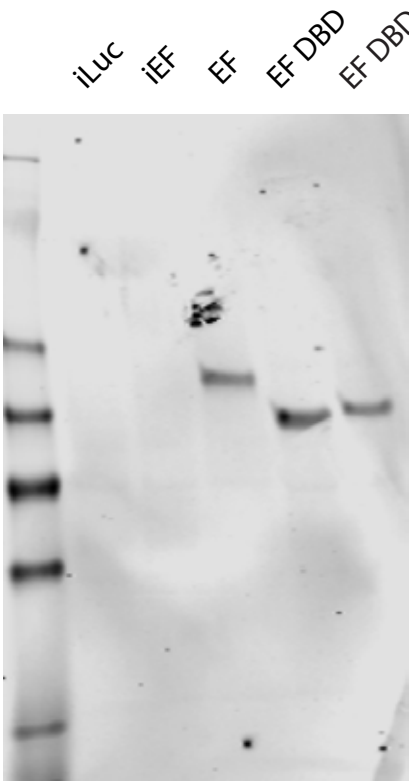

Tubulin

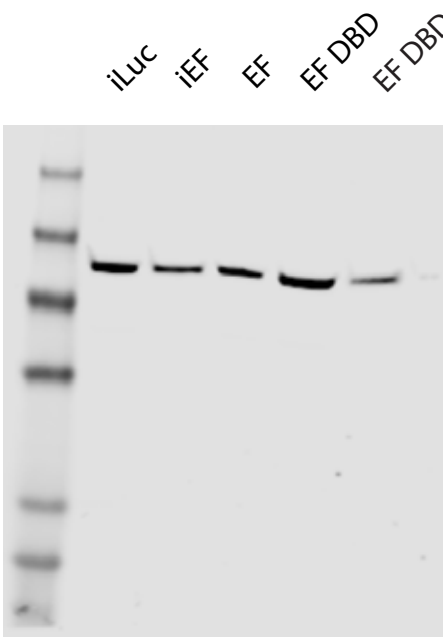

A673 Rep 2

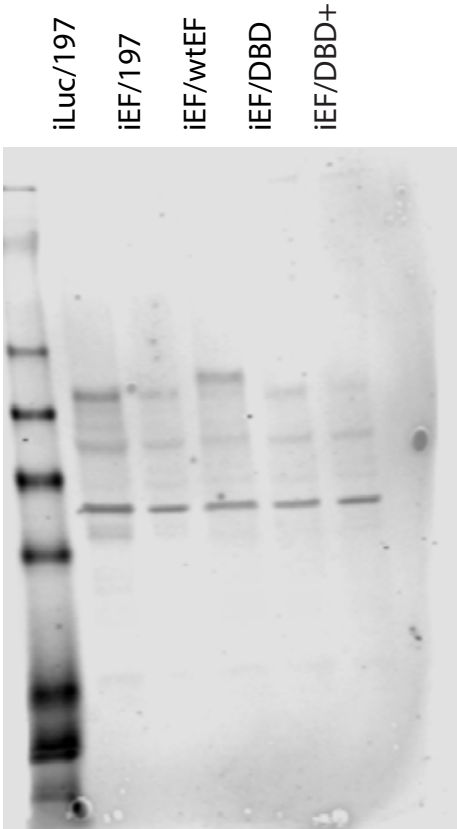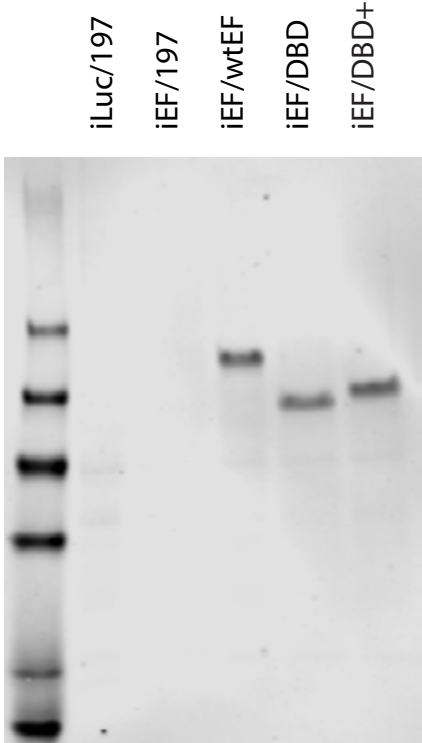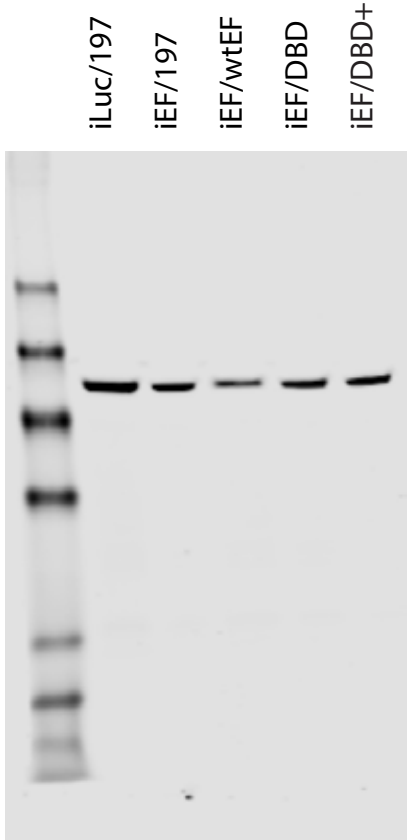

A673 Rep 3

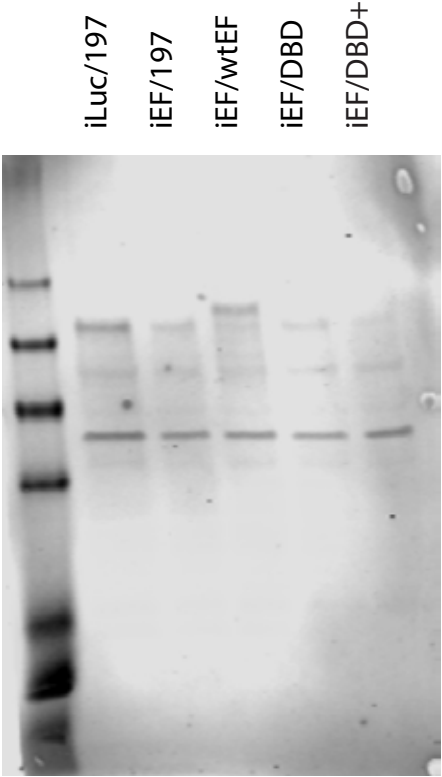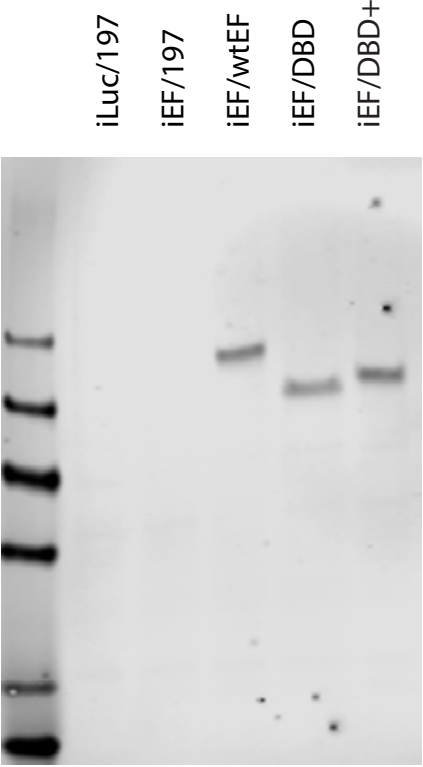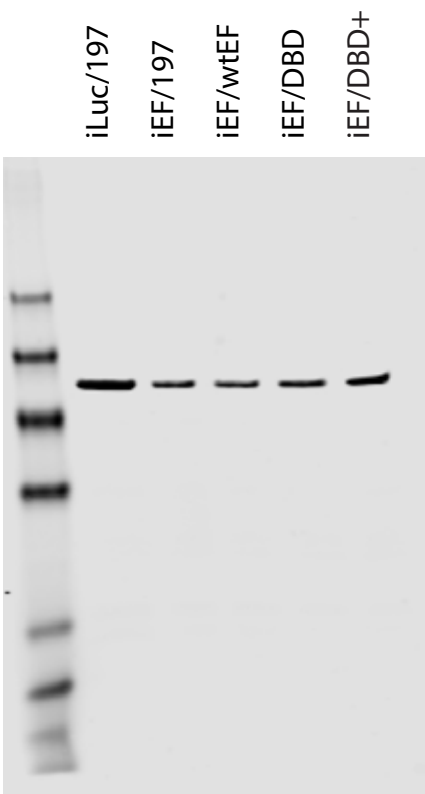

Supplement: Figure 1—figure supplement 1—source data 1. [file elife-95626-fig1-figsupp1-data1.zip › Labeled images/A673_FLI_FLAG_tubulin_rep_1_2_3_uncropped.pdf]

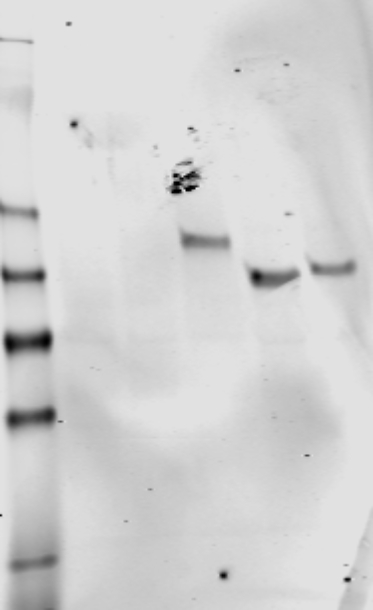

Supplement: Figure 1—figure supplement 1—source data 2. [file elife-95626-fig1-figsupp1-data2.zip › Raw images/Rep 1/FLAG_fullblotR1_169um.jpg]

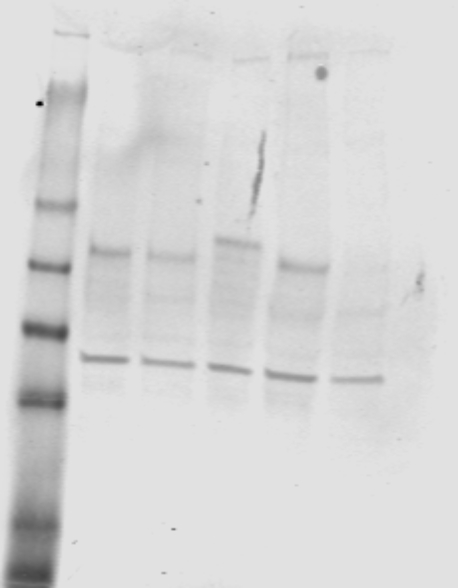

Supplement: Figure 1—figure supplement 1—source data 2. [file elife-95626-fig1-figsupp1-data2.zip › Raw images/Rep 1/Fli_fullblotR1_169um.jpg]

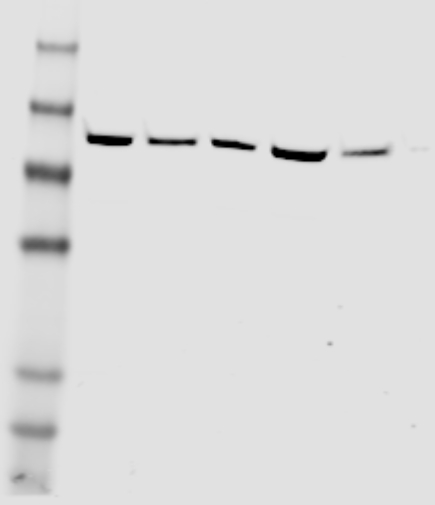

Supplement: Figure 1—figure supplement 1—source data 2. [file elife-95626-fig1-figsupp1-data2.zip › Raw images/Rep 1/tubulin_fullblotR1_169um.jpg]

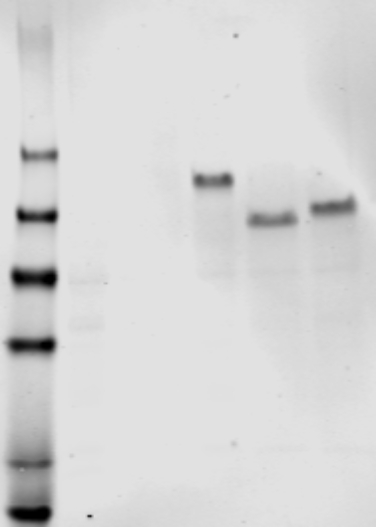

Supplement: Figure 1—figure supplement 1—source data 2. [file elife-95626-fig1-figsupp1-data2.zip › Raw images/Rep 2/FLAG_fullblotR2_169um.jpg]

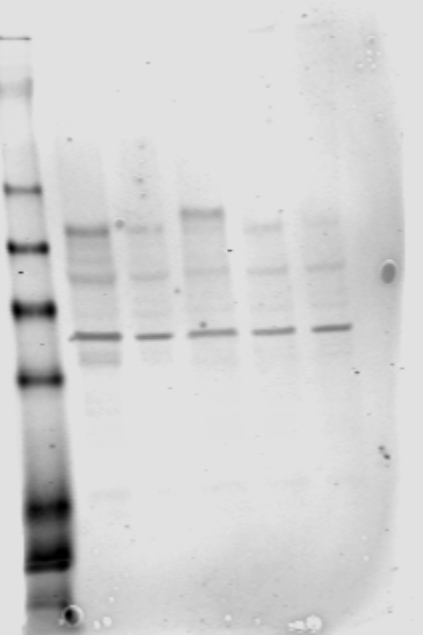

Supplement: Figure 1—figure supplement 1—source data 2. [file elife-95626-fig1-figsupp1-data2.zip › Raw images/Rep 2/Fli_fullblotR2_169um.jpg]

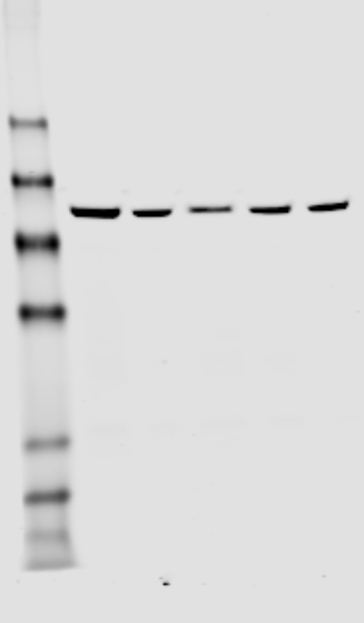

Supplement: Figure 1—figure supplement 1—source data 2. [file elife-95626-fig1-figsupp1-data2.zip › Raw images/Rep 2/tubulin_fullblotR2_169um.jpg]

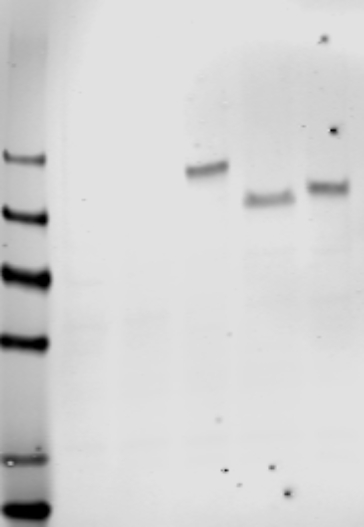

Supplement: Figure 1—figure supplement 1—source data 2. [file elife-95626-fig1-figsupp1-data2.zip › Raw images/Rep 3/FLAG_fullblotR3_169um.jpg]

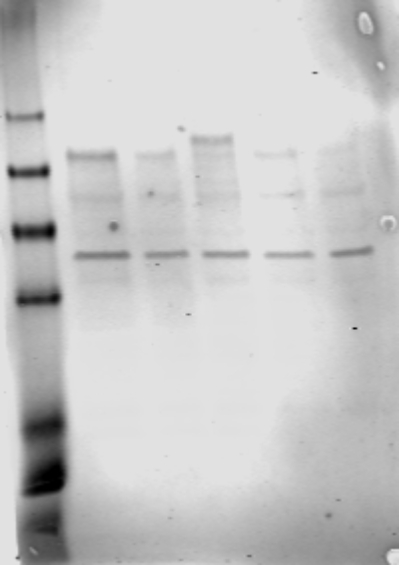

Supplement: Figure 1—figure supplement 1—source data 2. [file elife-95626-fig1-figsupp1-data2.zip › Raw images/Rep 3/Fli_fullblotR3_169um.jpg]

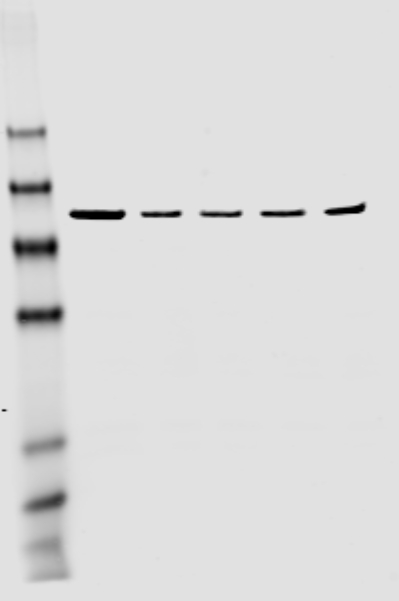

Supplement: Figure 1—figure supplement 1—source data 2. [file elife-95626-fig1-figsupp1-data2.zip › Raw images/Rep 3/tubulin_fullblotR3_169um.jpg]

iEF

EF

EF DBD

EF DBD+

Tech rep 1

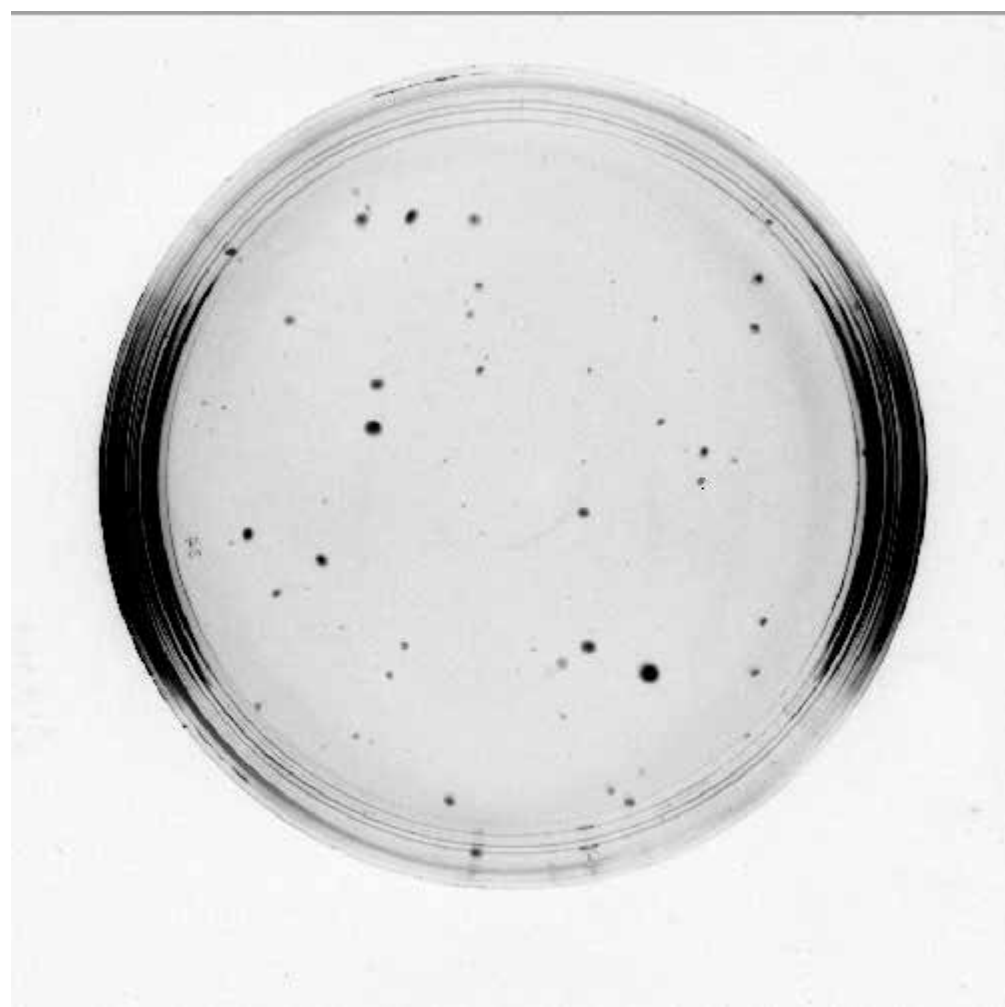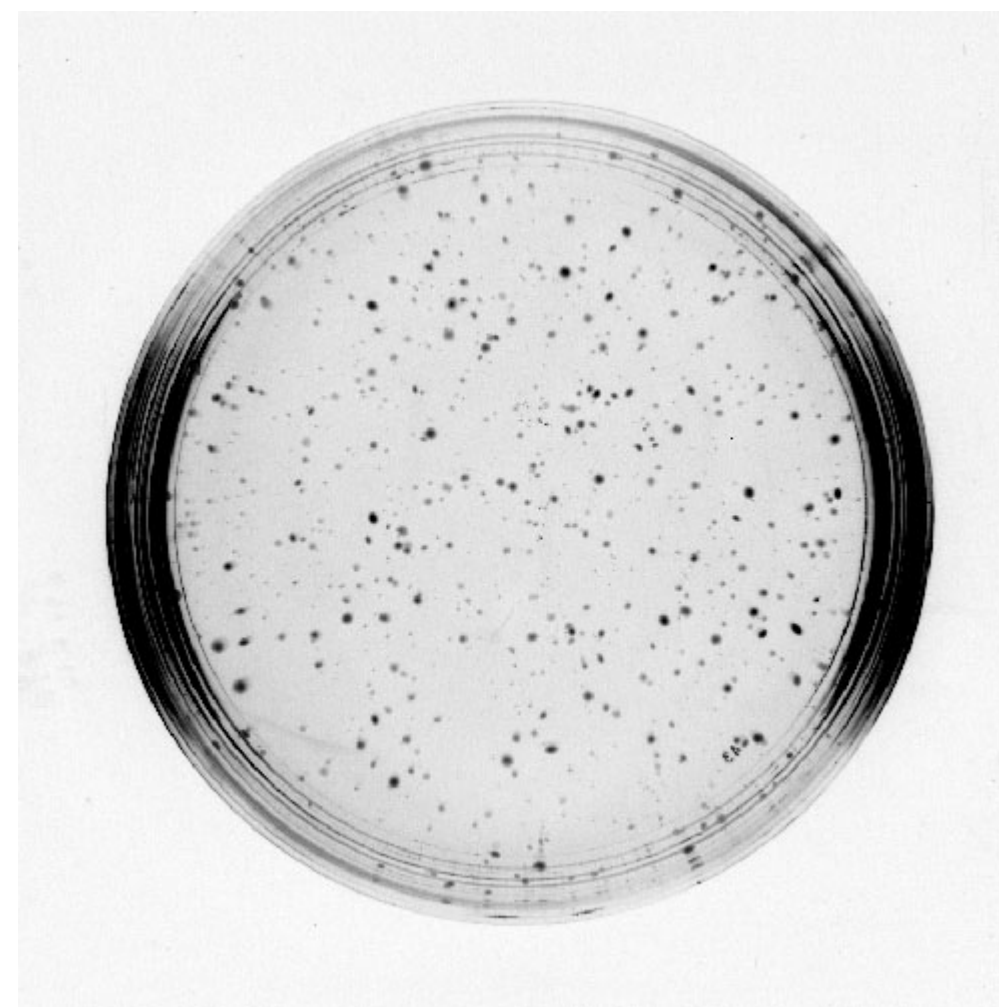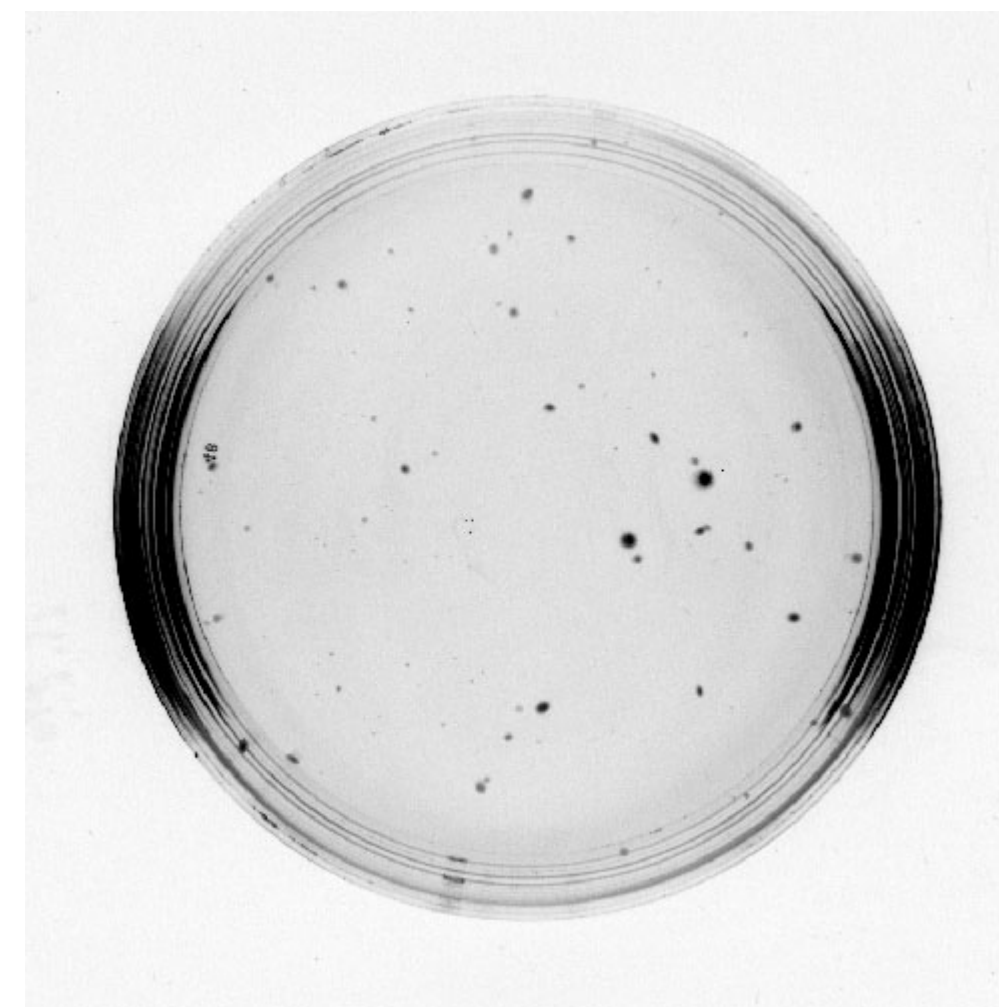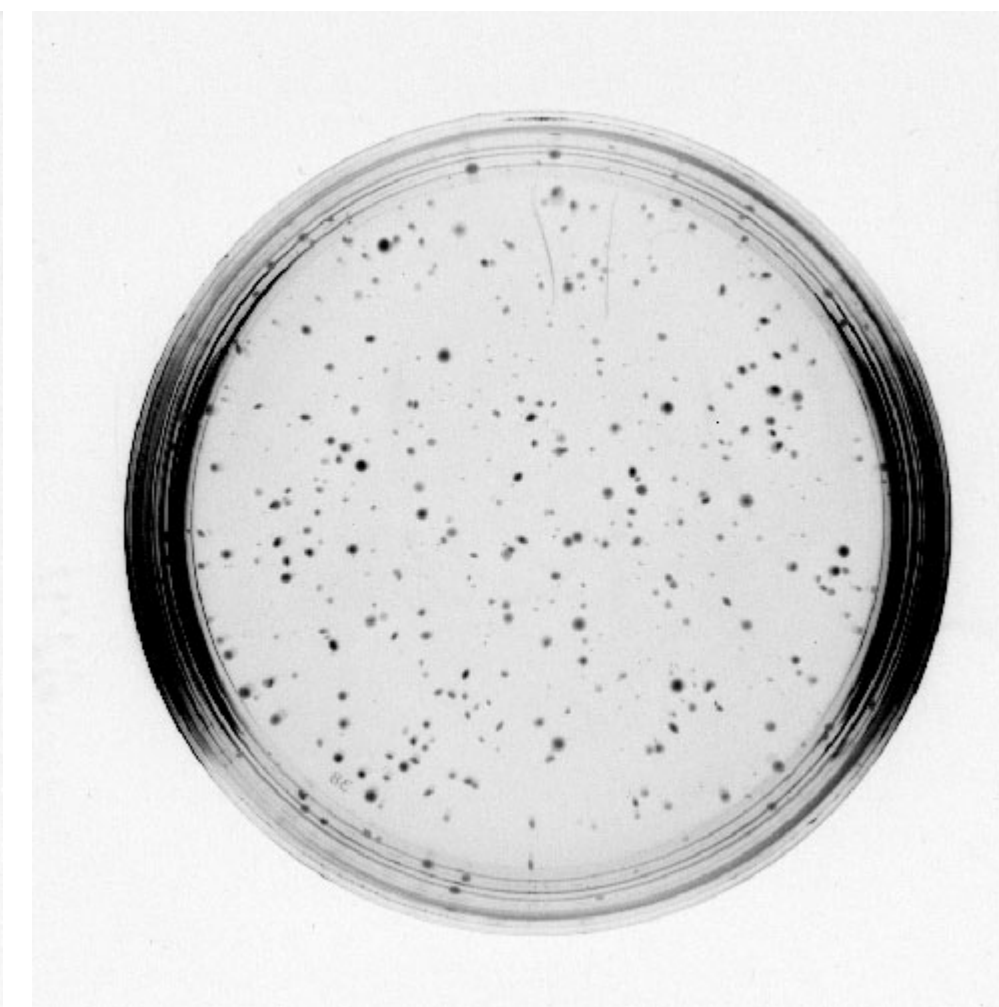

Tech rep 2

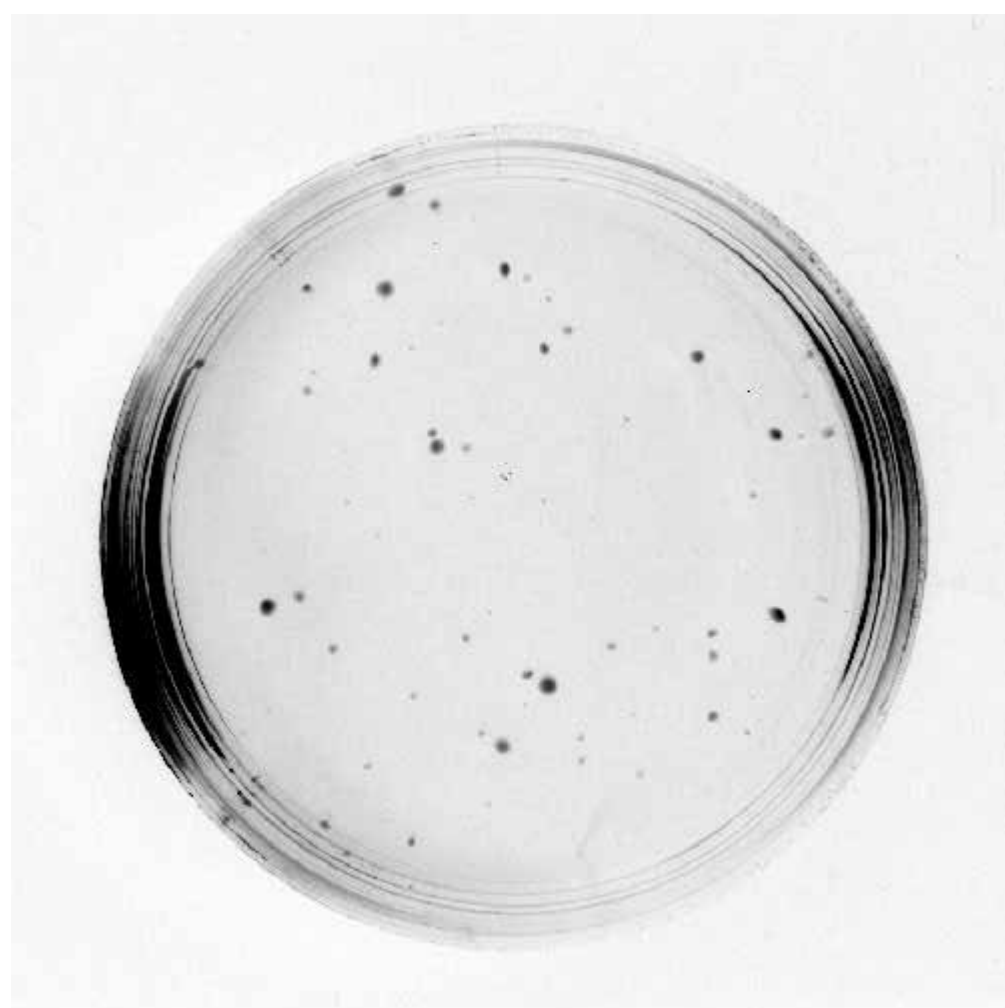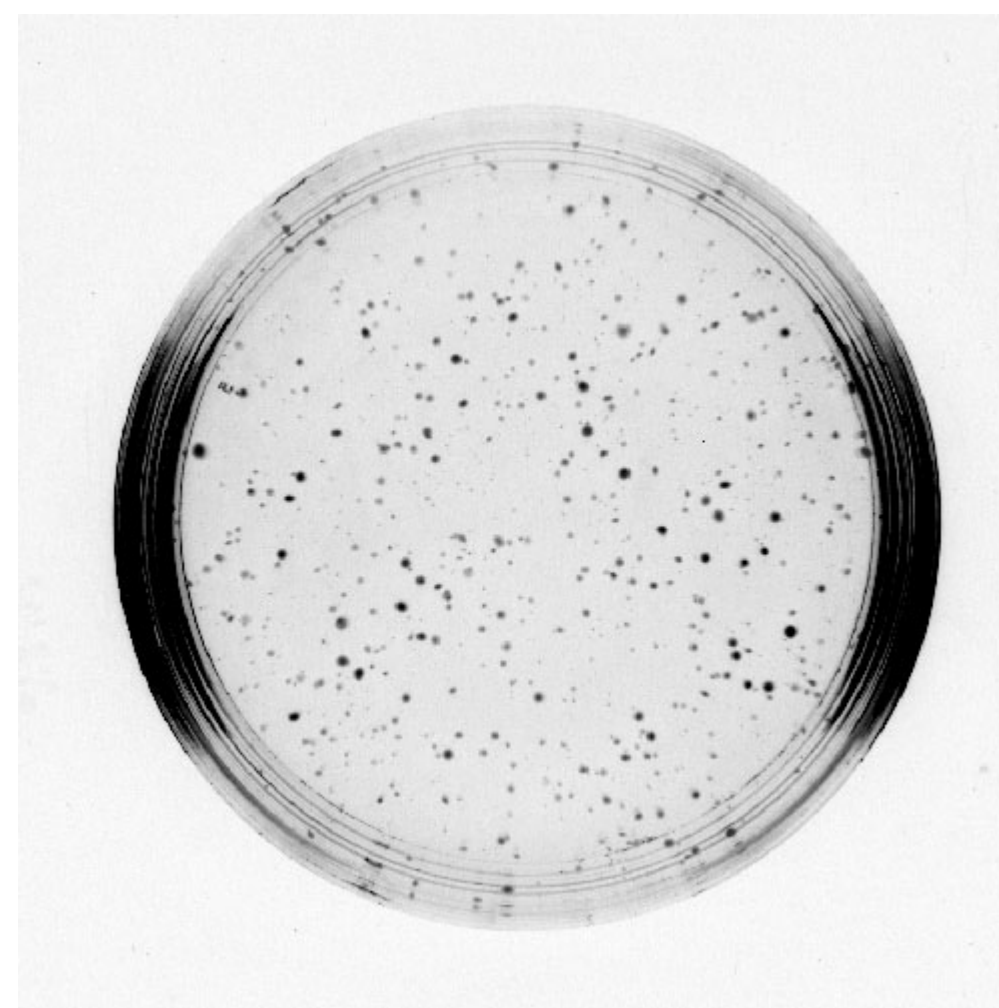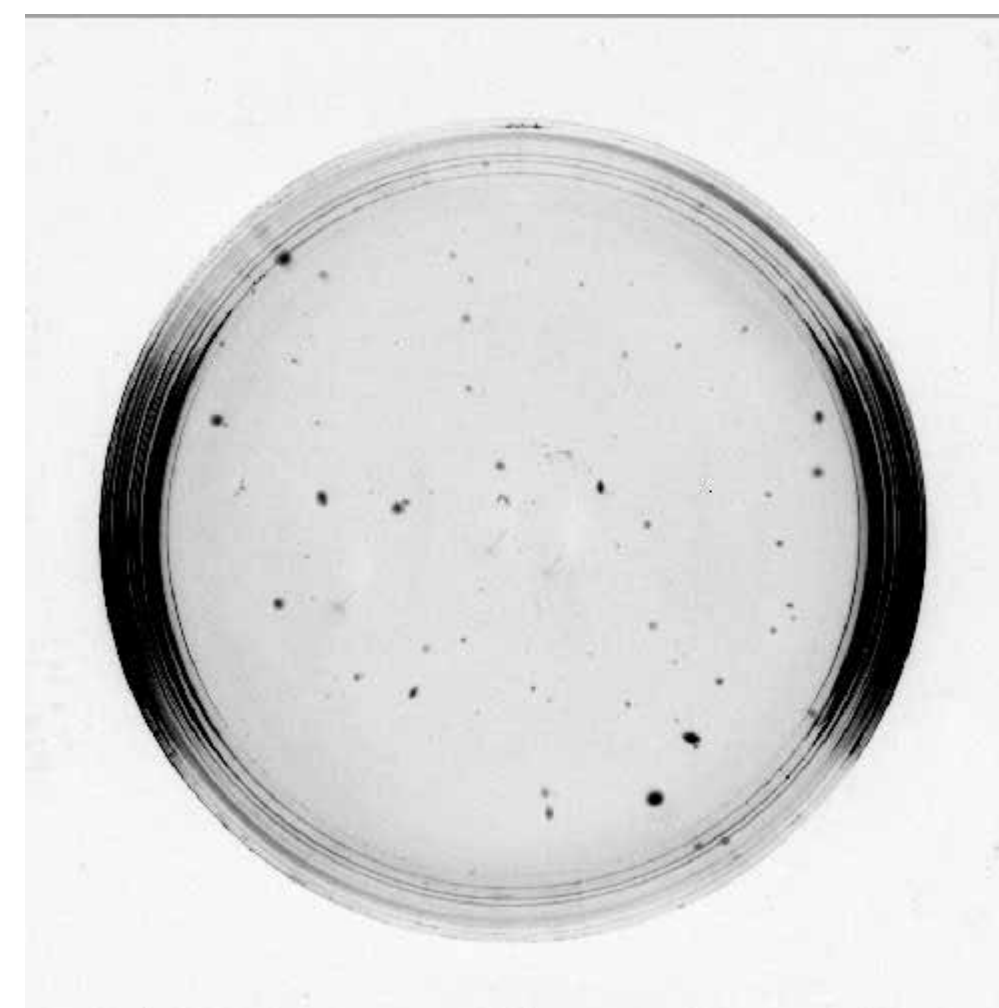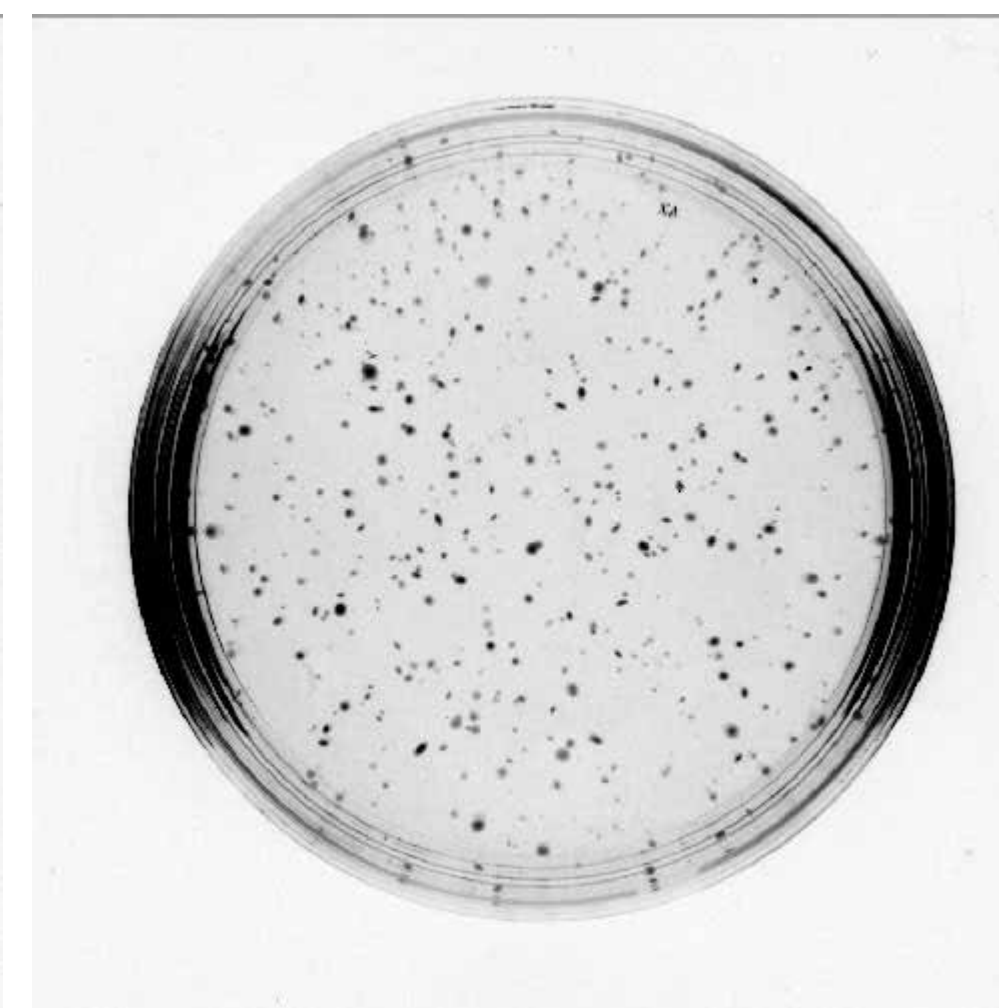

A673 Rep 1

Supplement: Figure 1—figure supplement 1—source data 3. [file elife-95626-fig1-figsupp1-data3.zip › Labelled images/R1_submission.pdf]

iEF/197

iEF/wtEF

iEF/DBD

iEF/DBD+

Tech rep 1

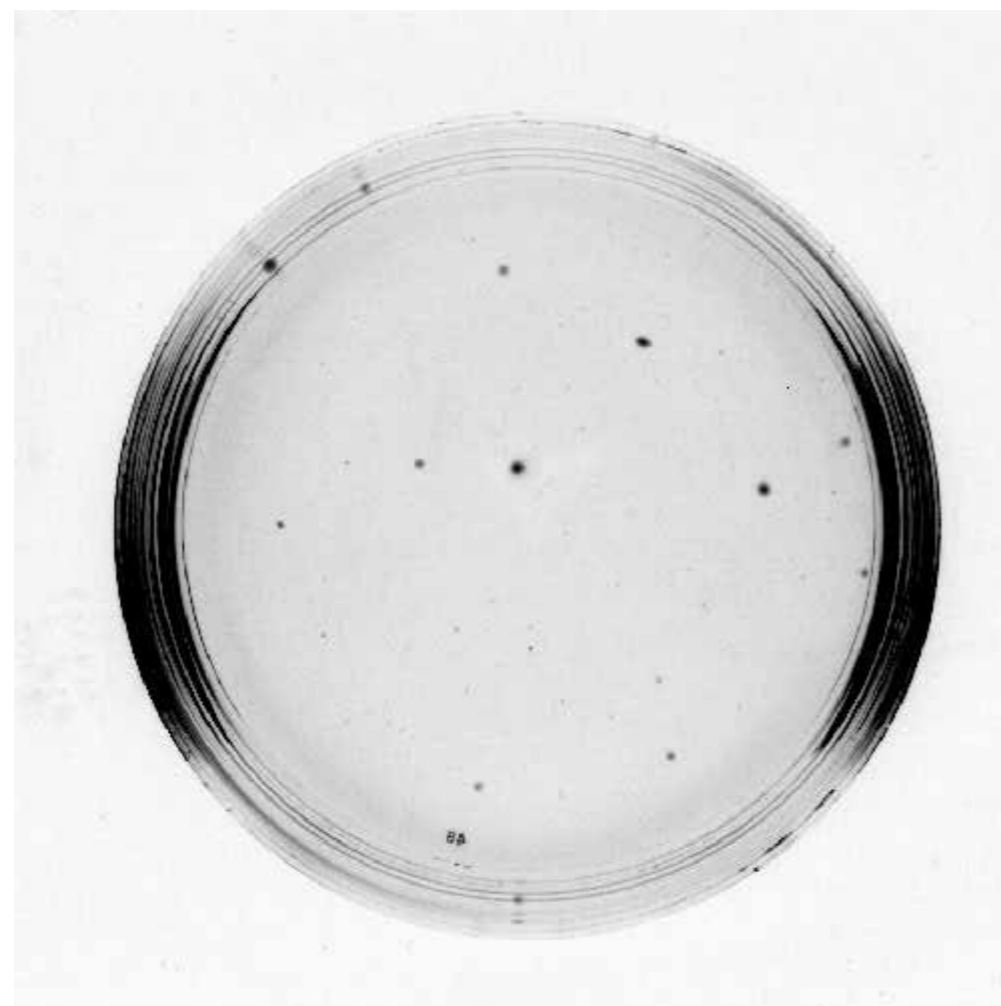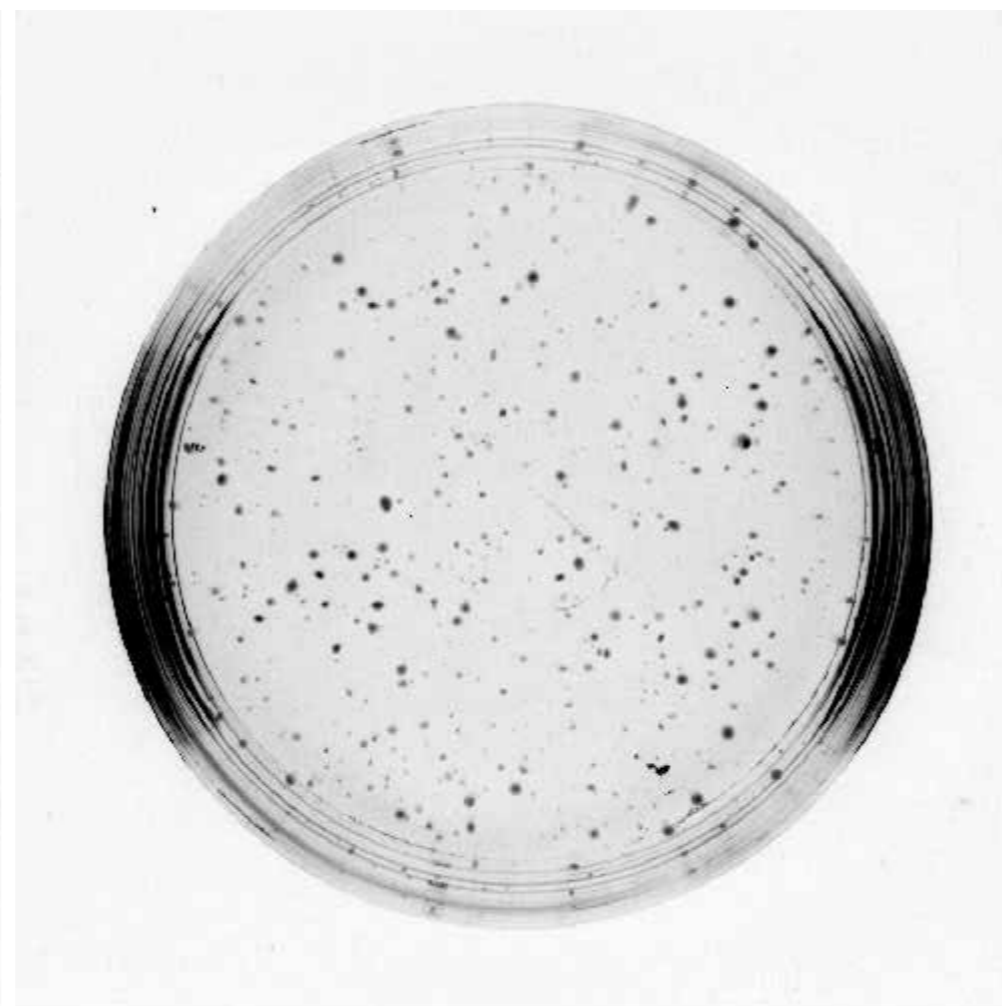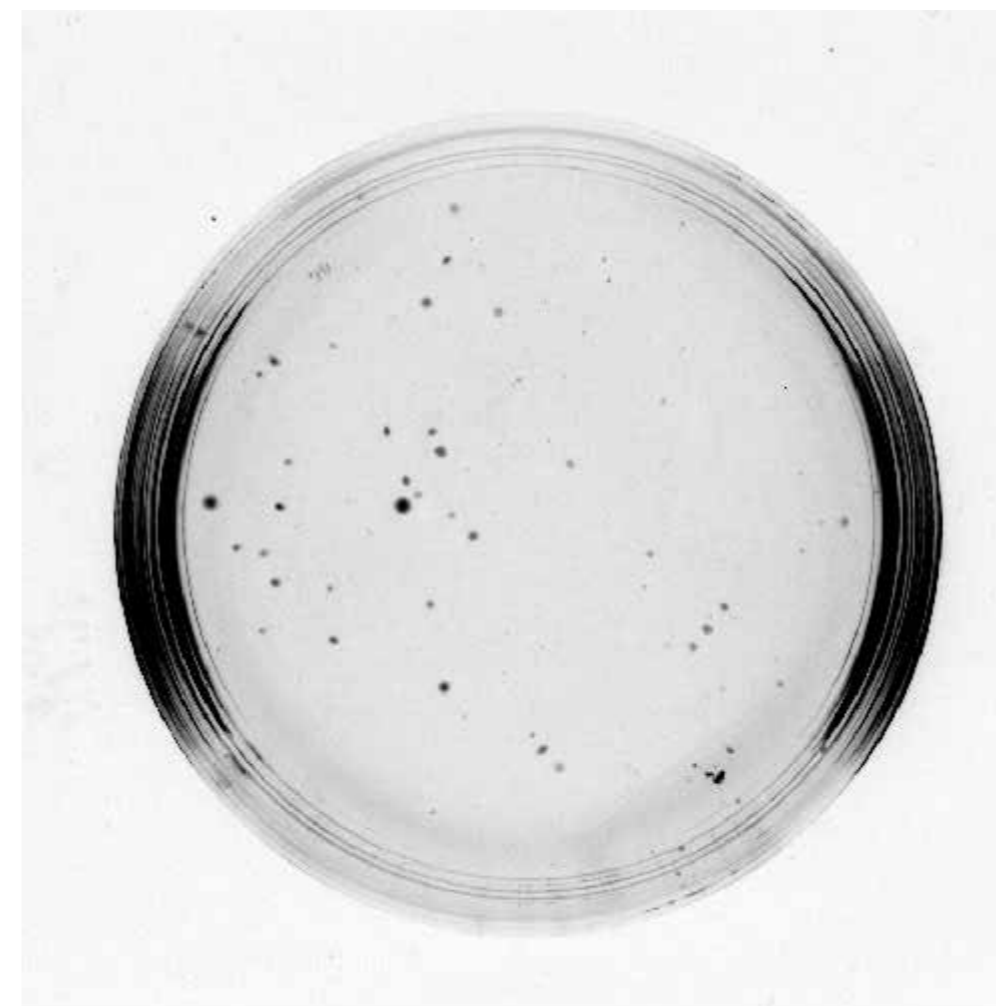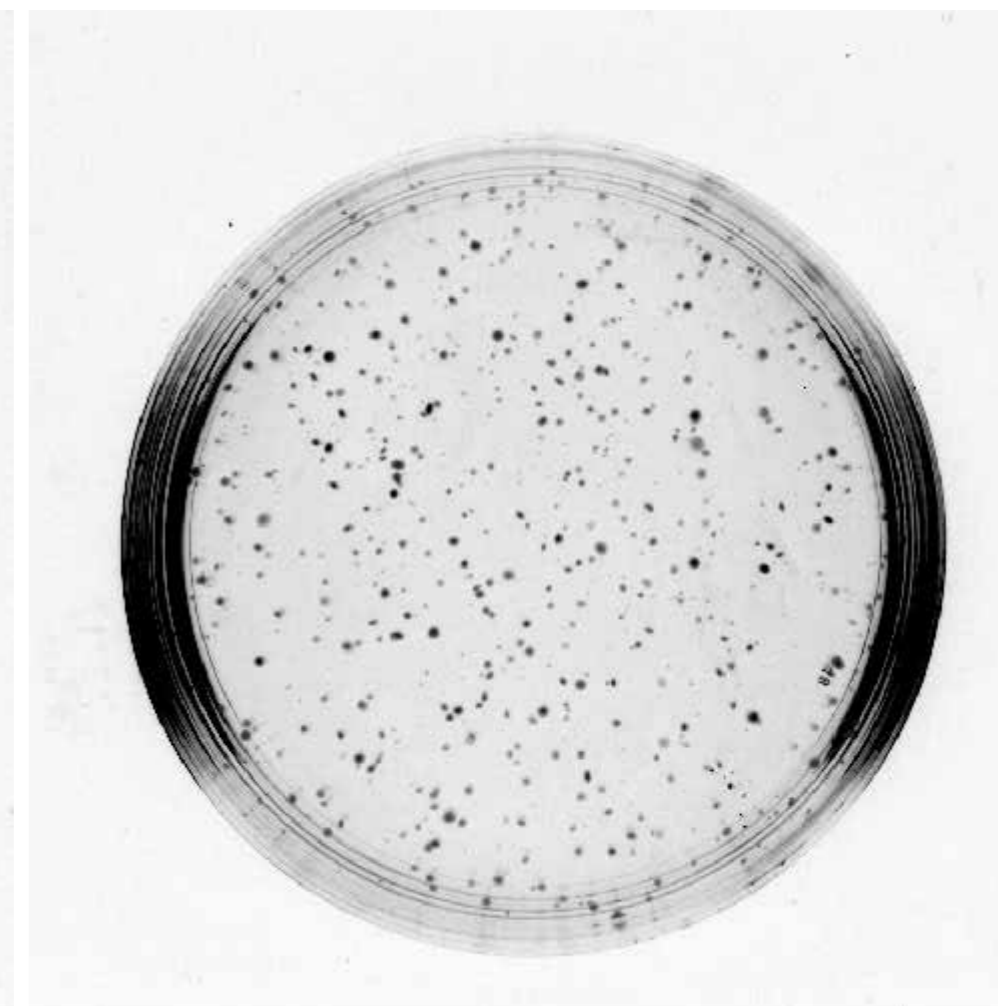

Tech rep 2

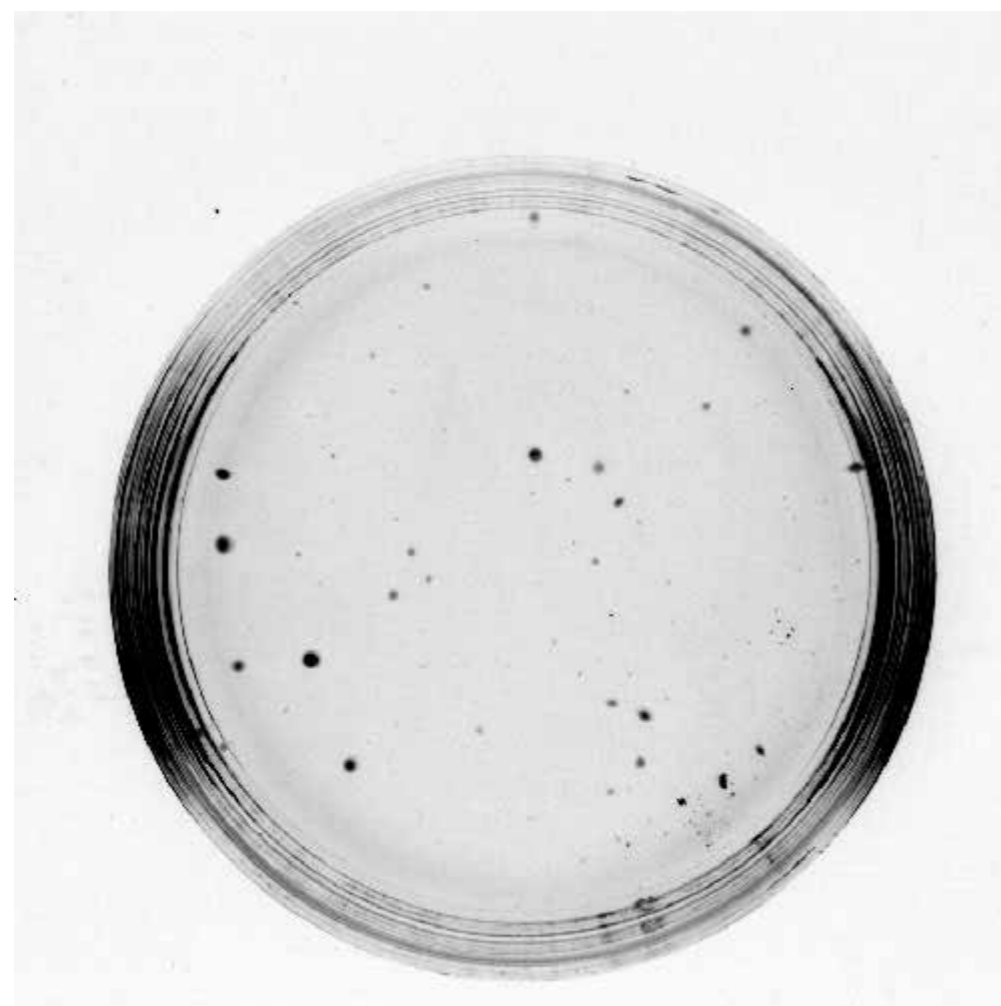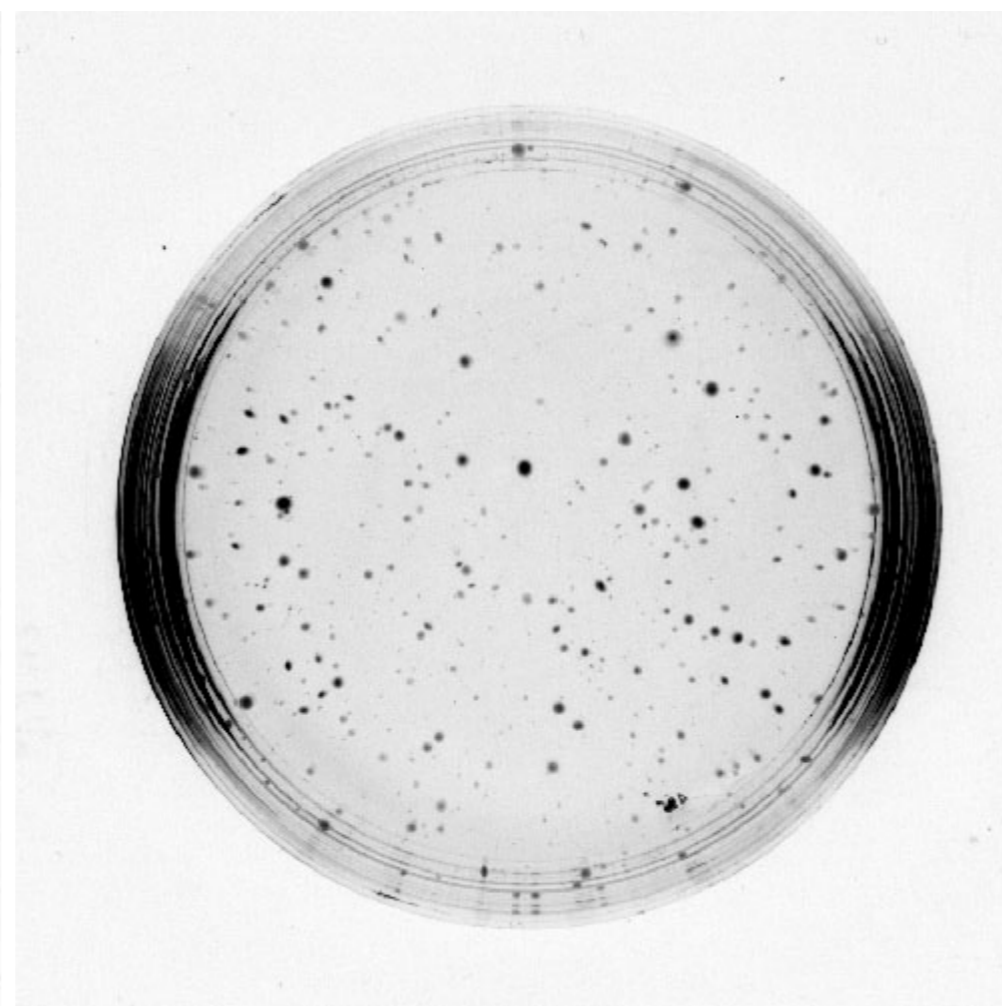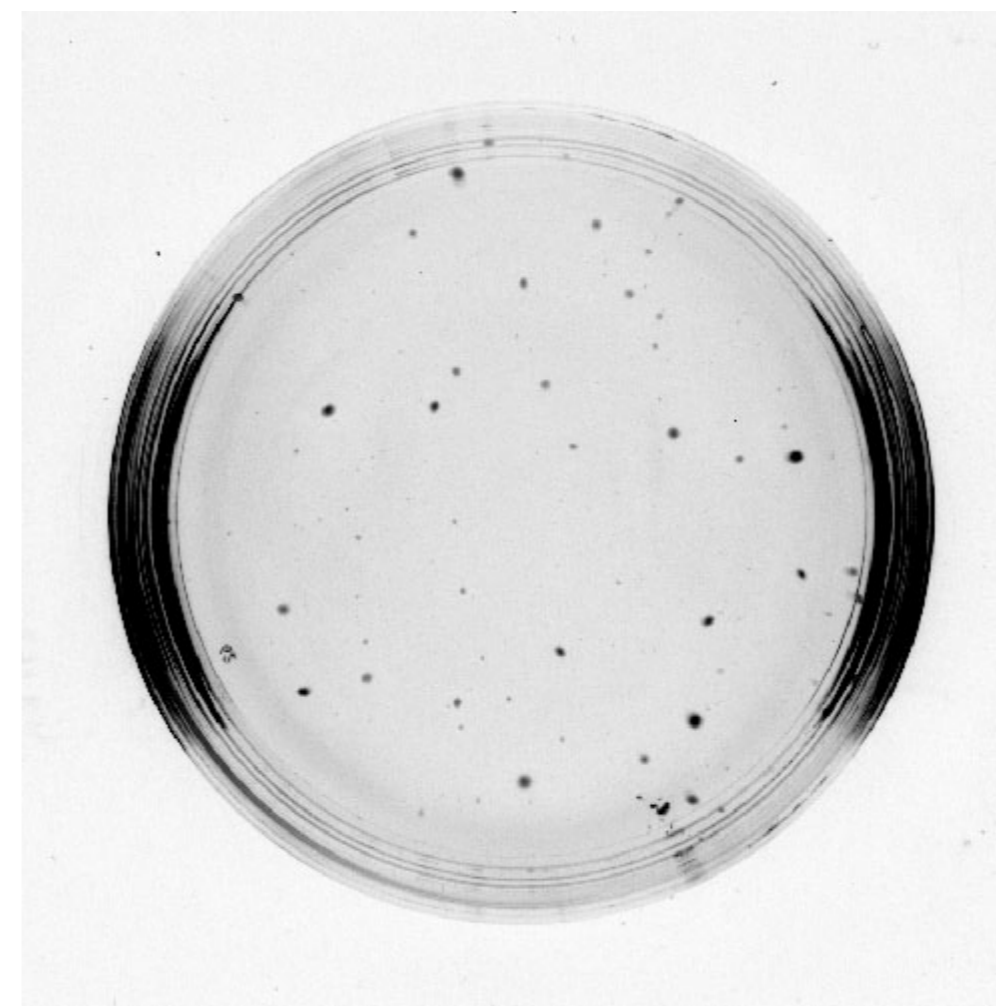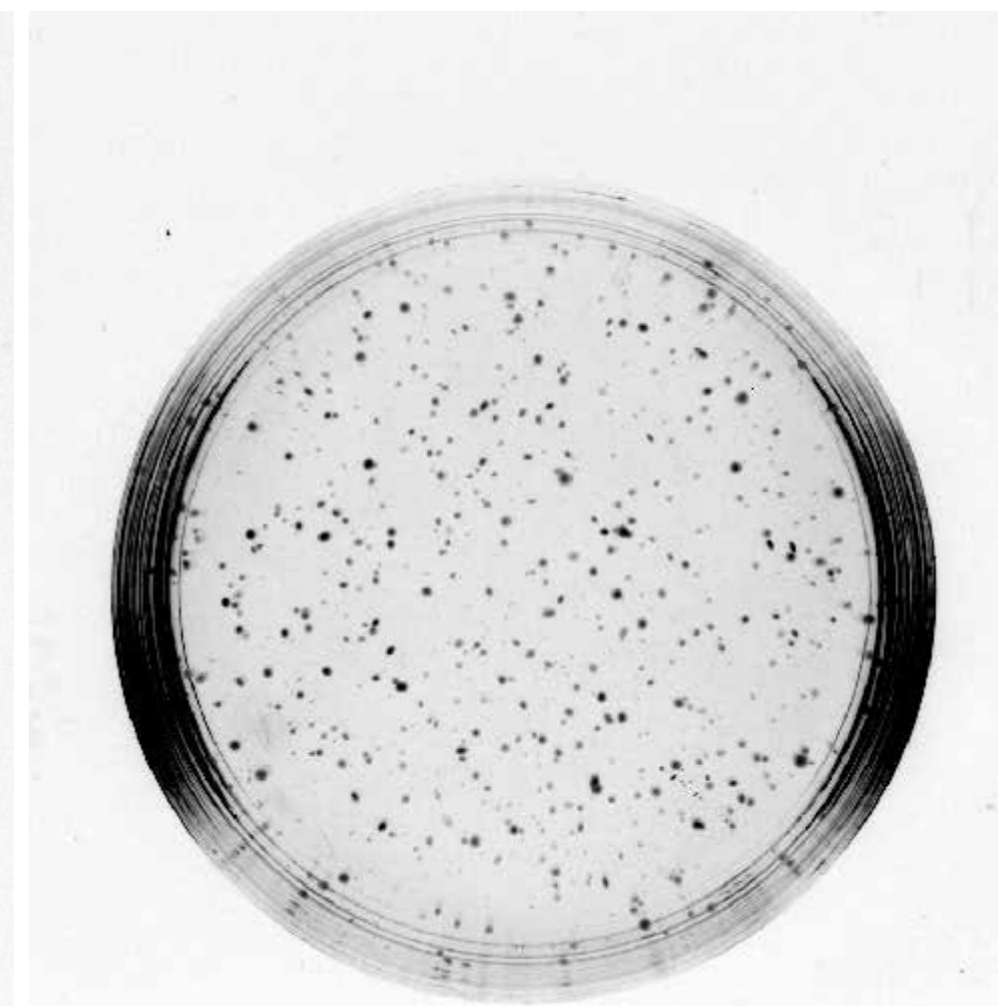

A673 Rep 2

Supplement: Figure 1—figure supplement 1—source data 3. [file elife-95626-fig1-figsupp1-data3.zip › Labelled images/R2_submission.pdf]

iEF/197

iEF/wtEF

iEF/DBD

iEF/DBD+

Tech rep 1

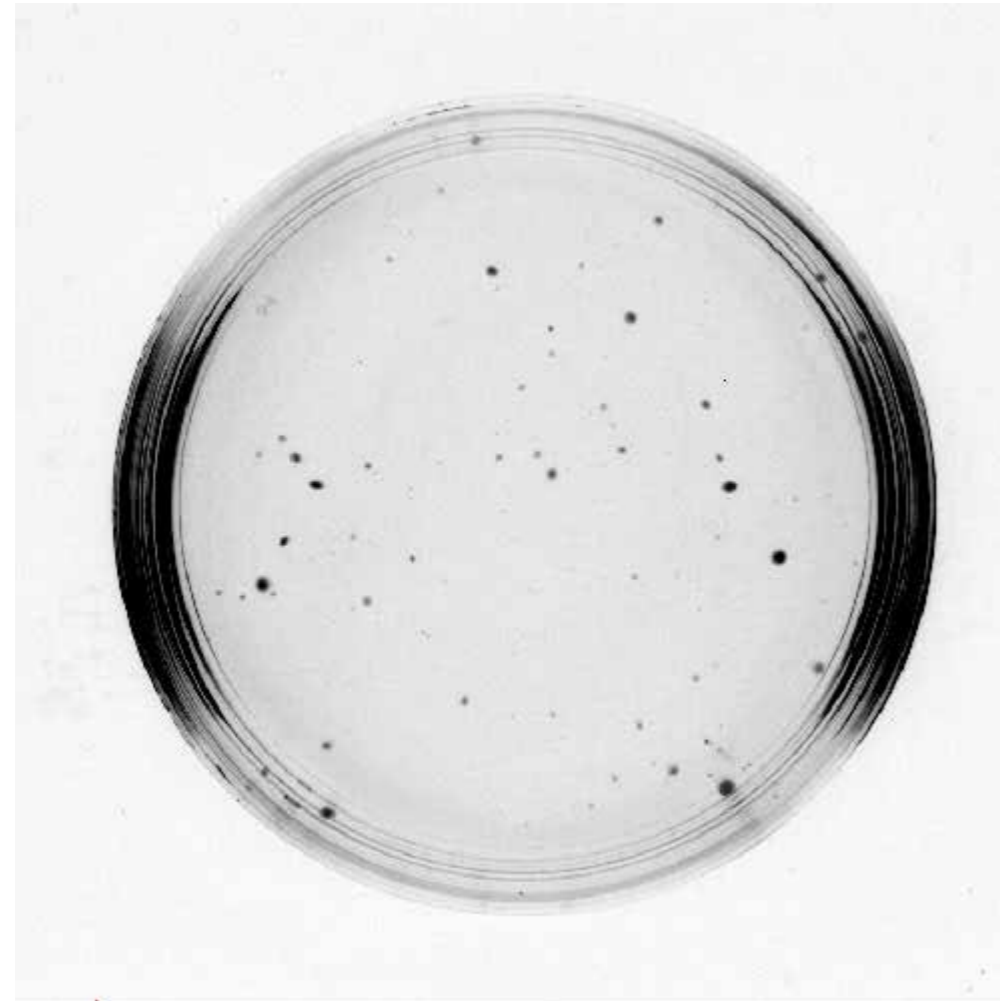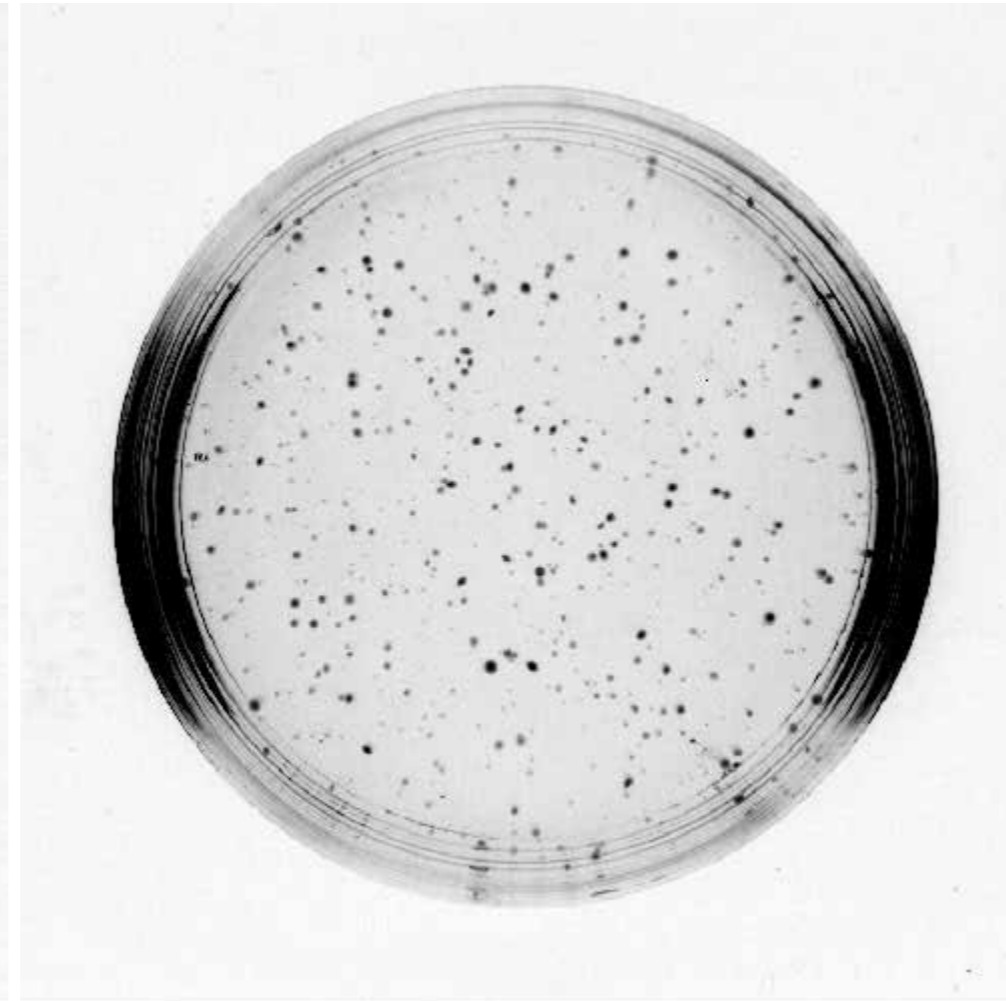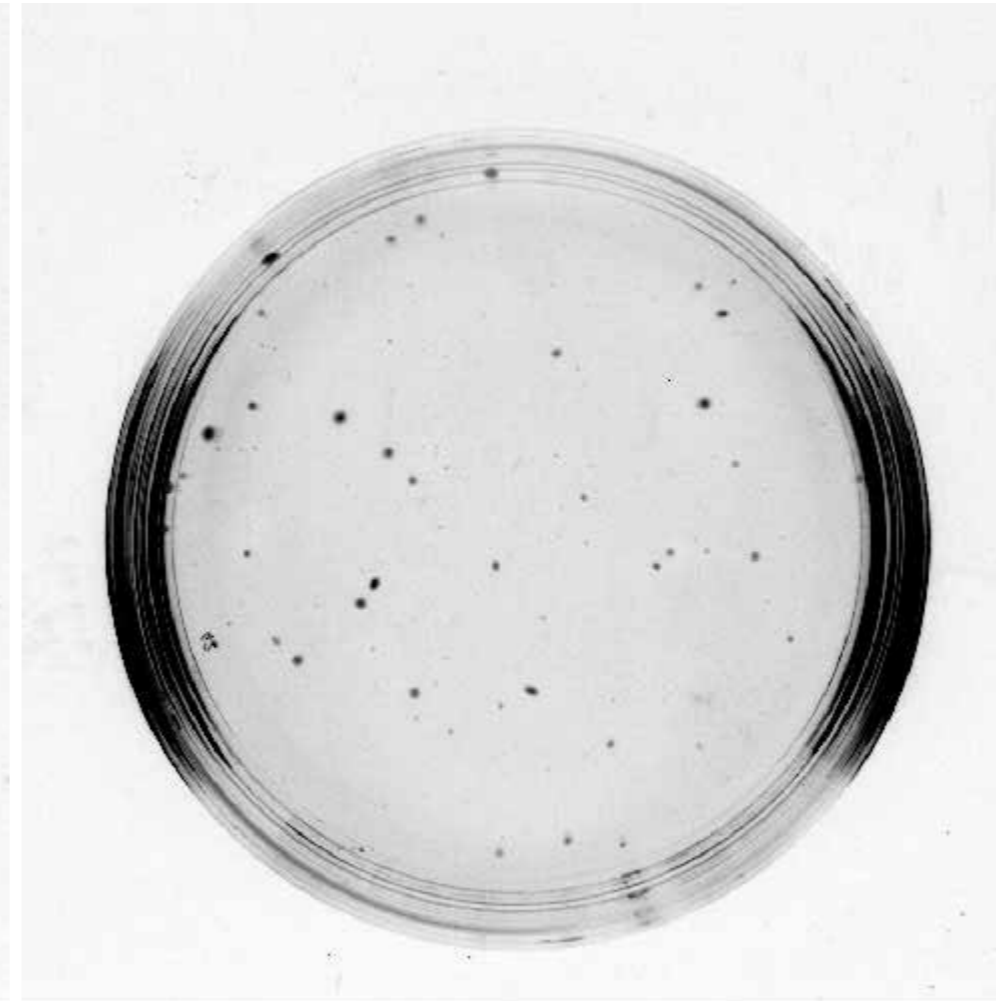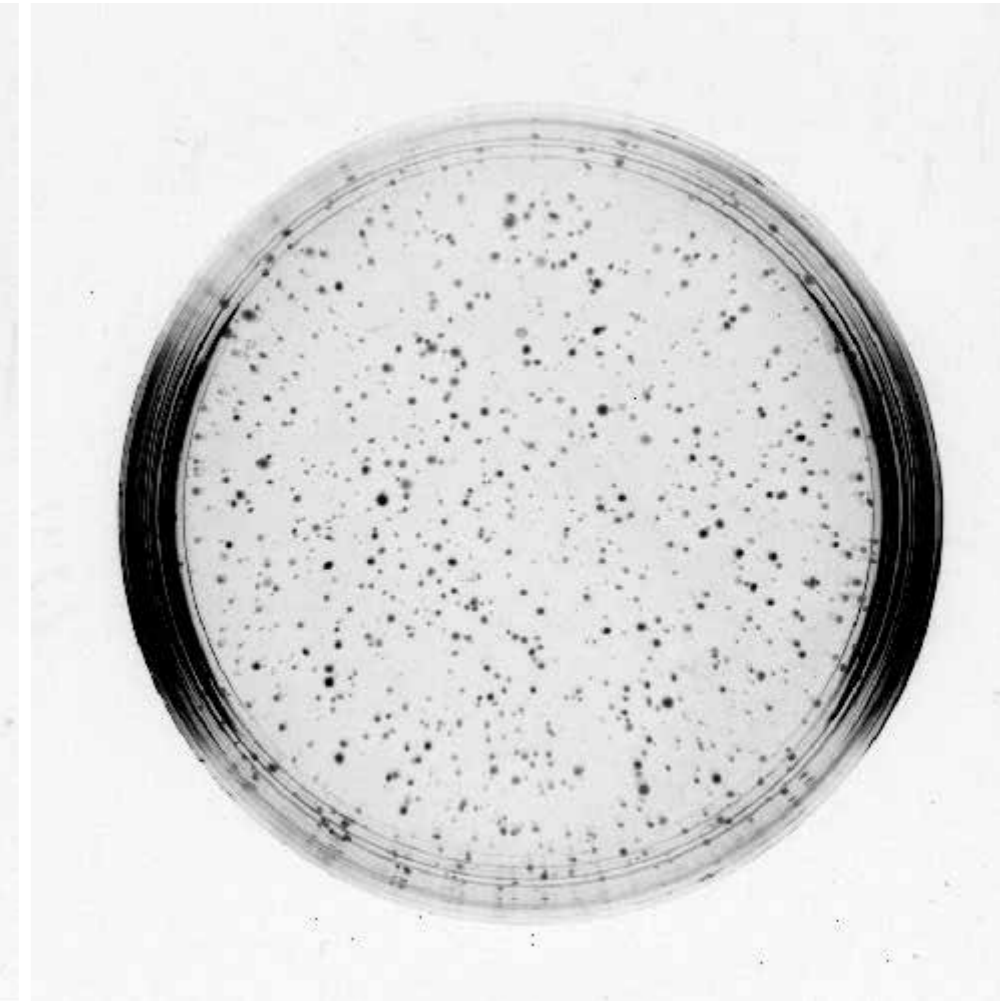

Tech rep 2

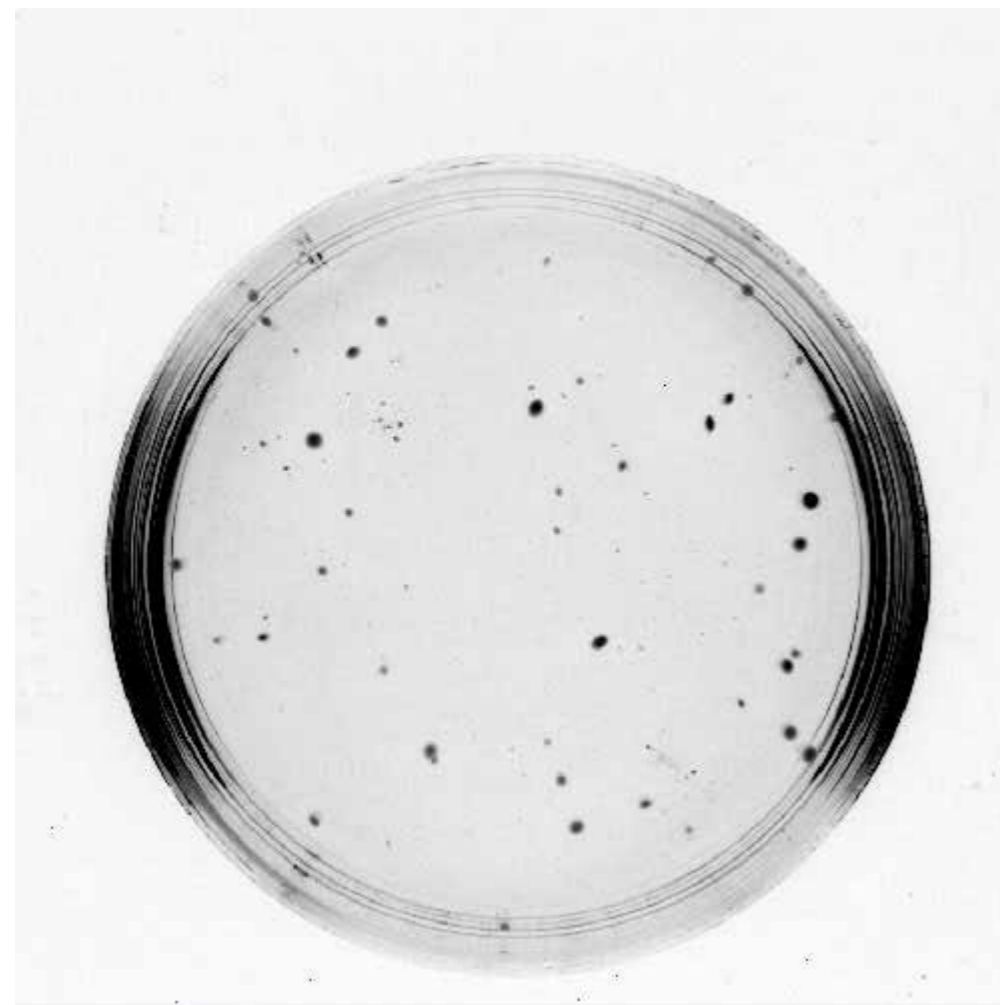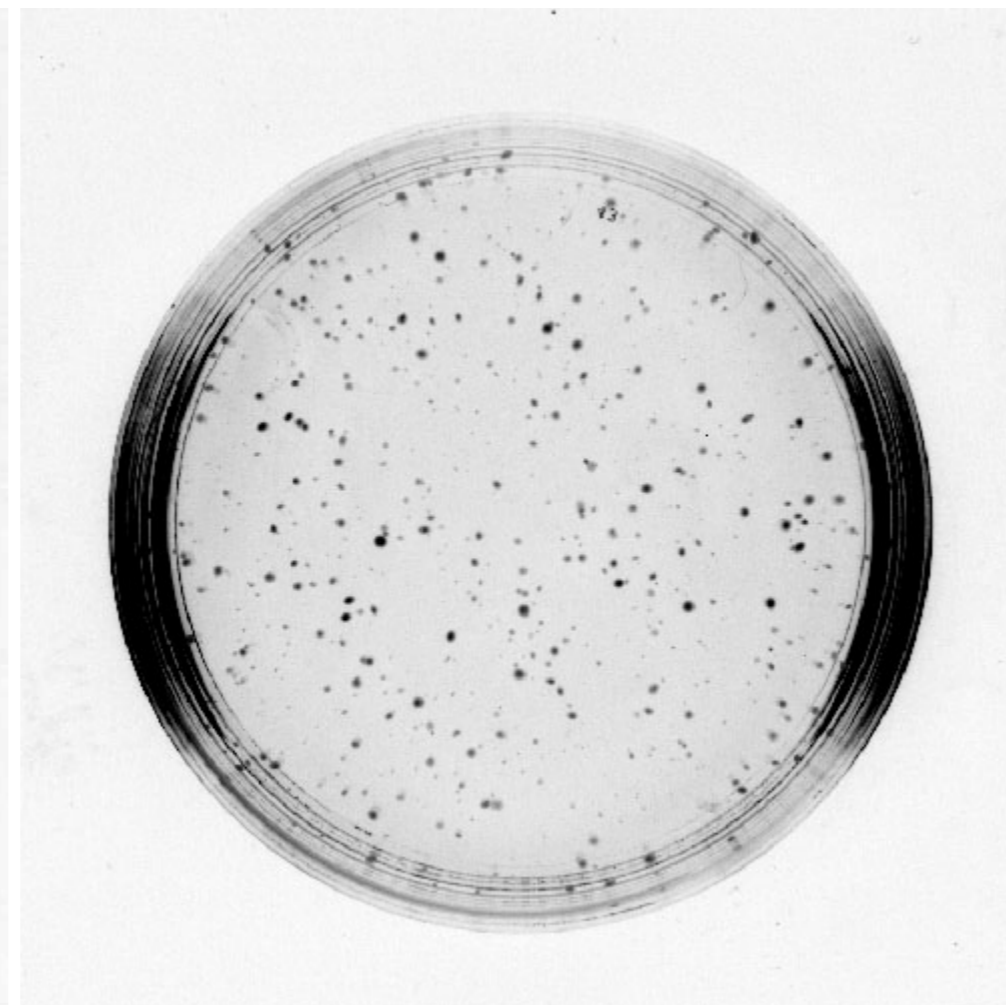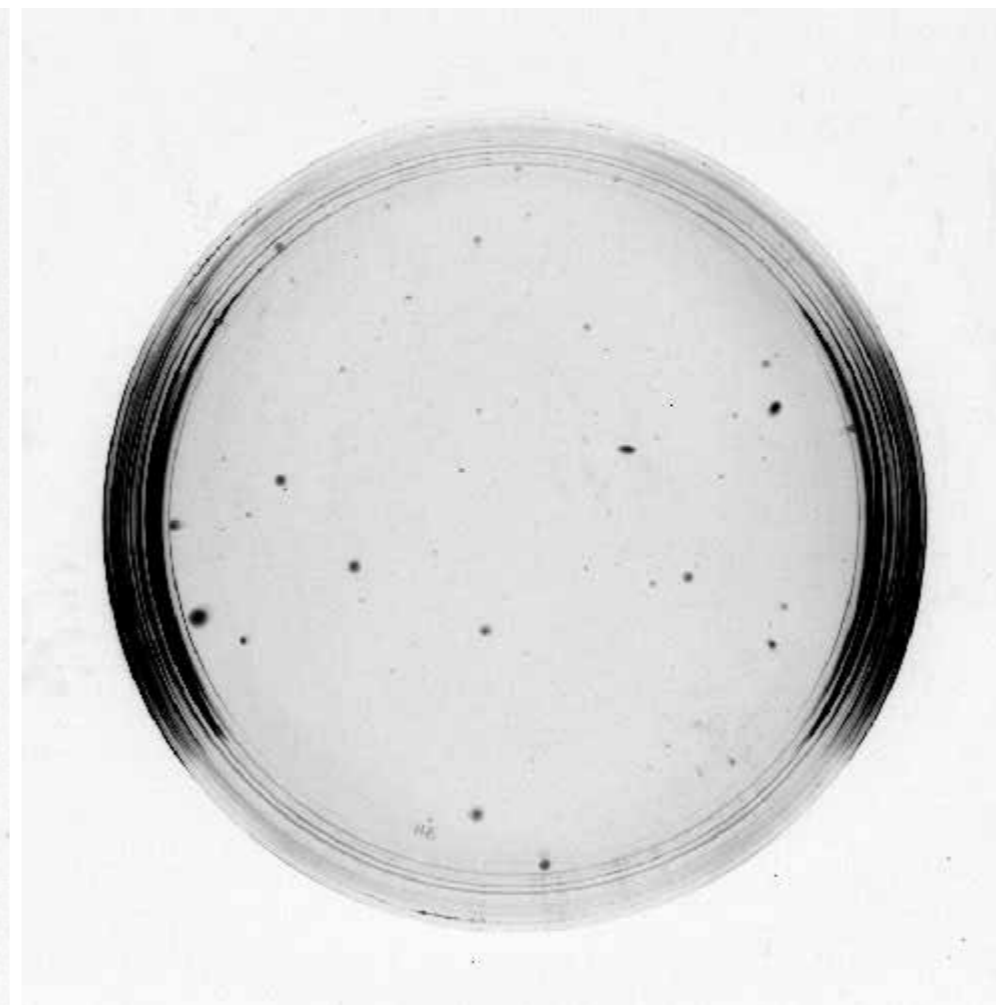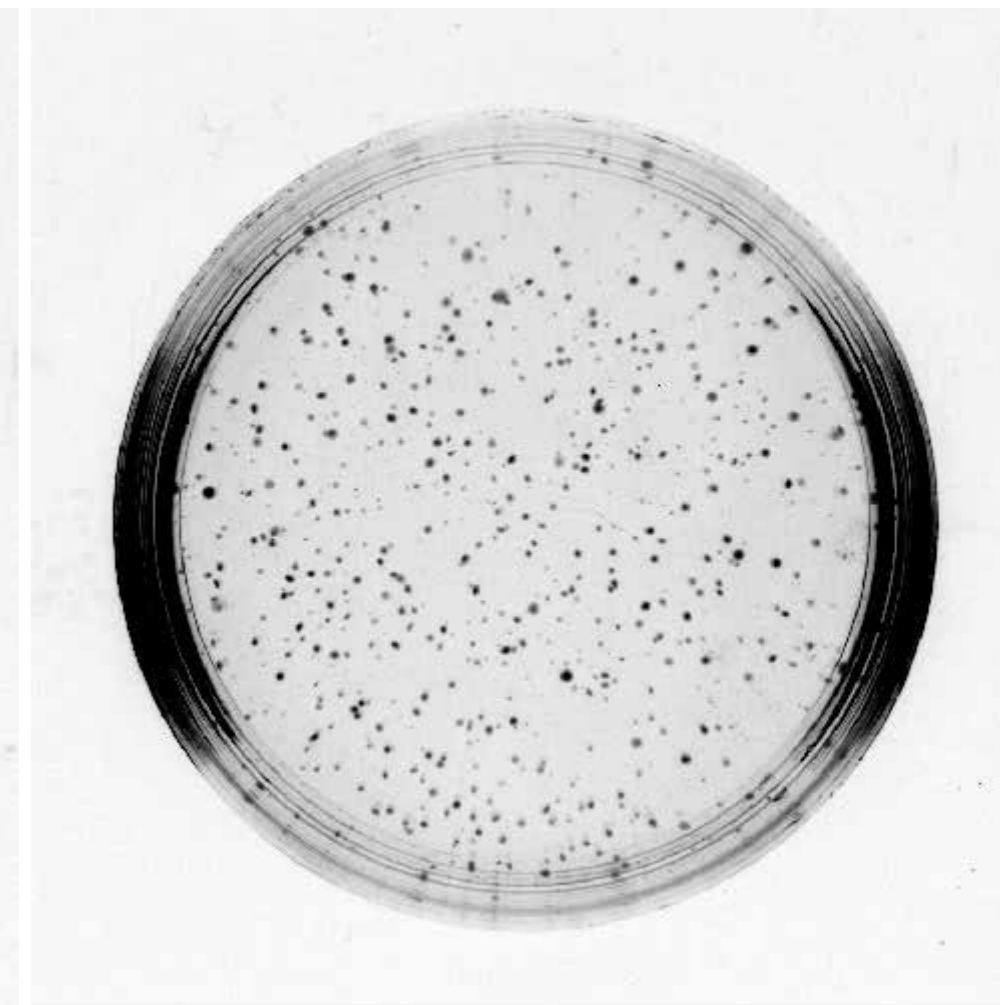

A673 Rep 3

Supplement: Figure 1—figure supplement 1—source data 3. [file elife-95626-fig1-figsupp1-data3.zip › Labelled images/R3_submission.pdf]

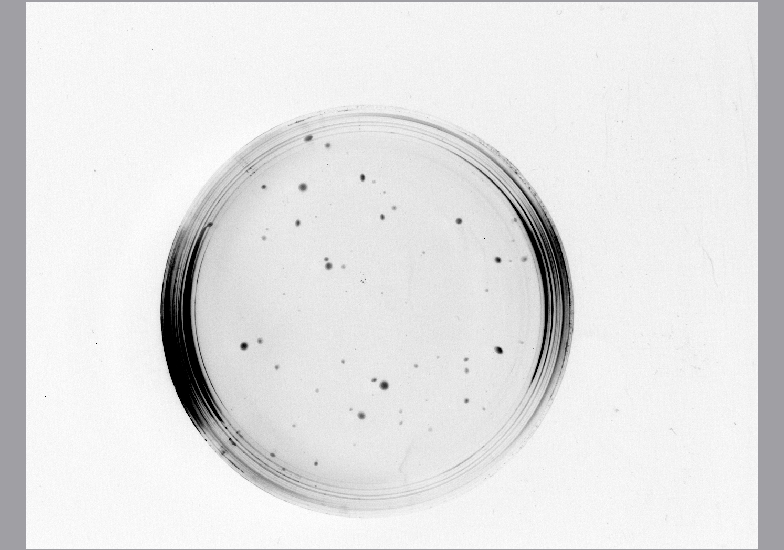

Supplement: Figure 1—figure supplement 1—source data 4. [file elife-95626-fig1-figsupp1-data4.zip › Raw images/Rep 1/A673_ief197_R1_1.jpg]

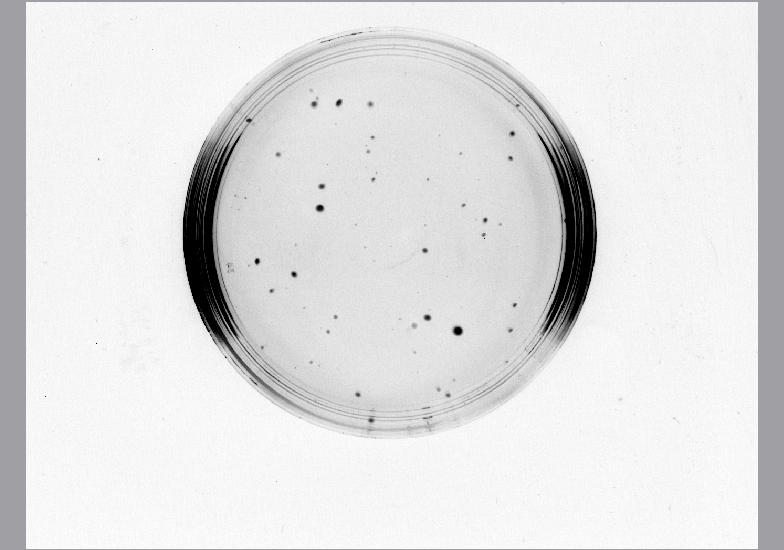

Supplement: Figure 1—figure supplement 1—source data 4. [file elife-95626-fig1-figsupp1-data4.zip › Raw images/Rep 1/A673_ief197_R1_2.jpg]

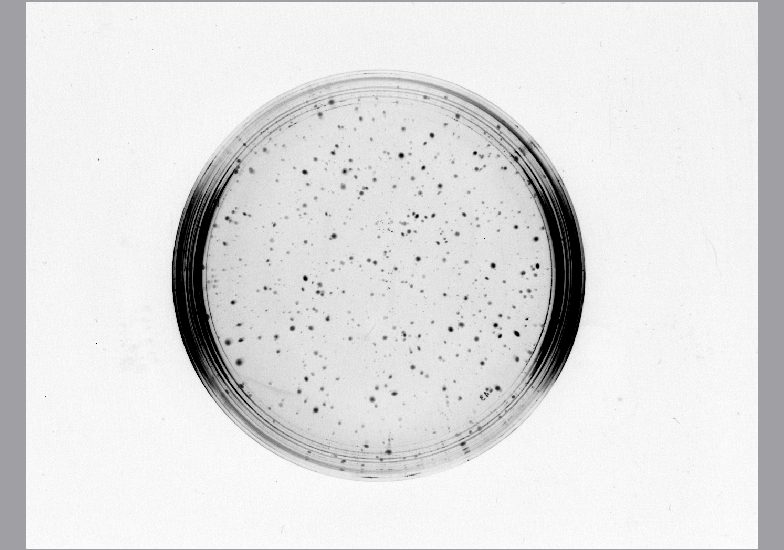

Supplement: Figure 1—figure supplement 1—source data 4. [file elife-95626-fig1-figsupp1-data4.zip › Raw images/Rep 1/A673_ief714_R1_1.jpg]

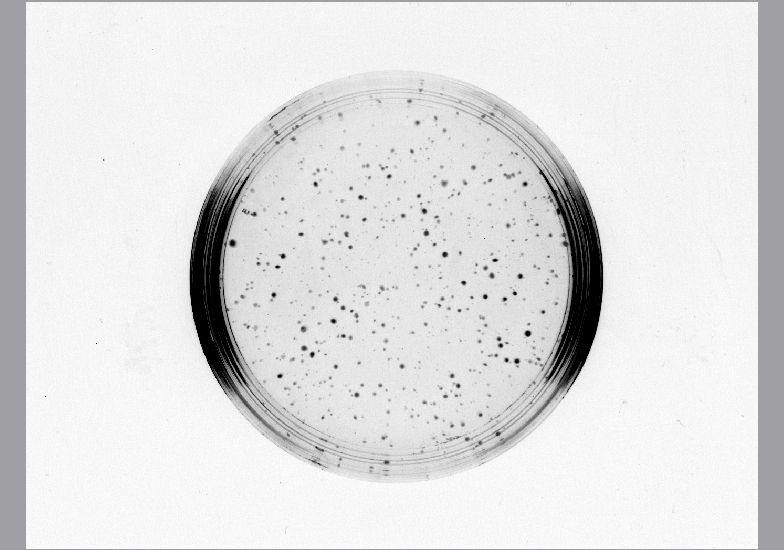

Supplement: Figure 1—figure supplement 1—source data 4. [file elife-95626-fig1-figsupp1-data4.zip › Raw images/Rep 1/A673_ief714_R1_2.jpg]

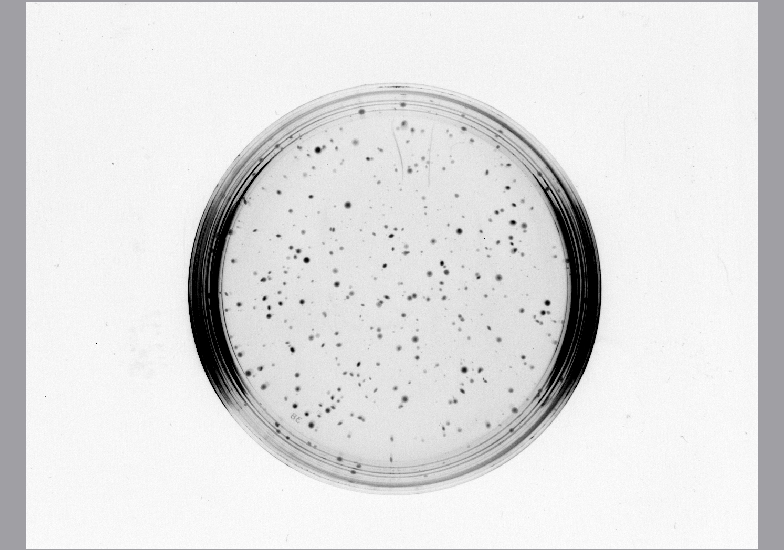

Supplement: Figure 1—figure supplement 1—source data 4. [file elife-95626-fig1-figsupp1-data4.zip › Raw images/Rep 1/A673_iefDBD+_R1_1.jpg]

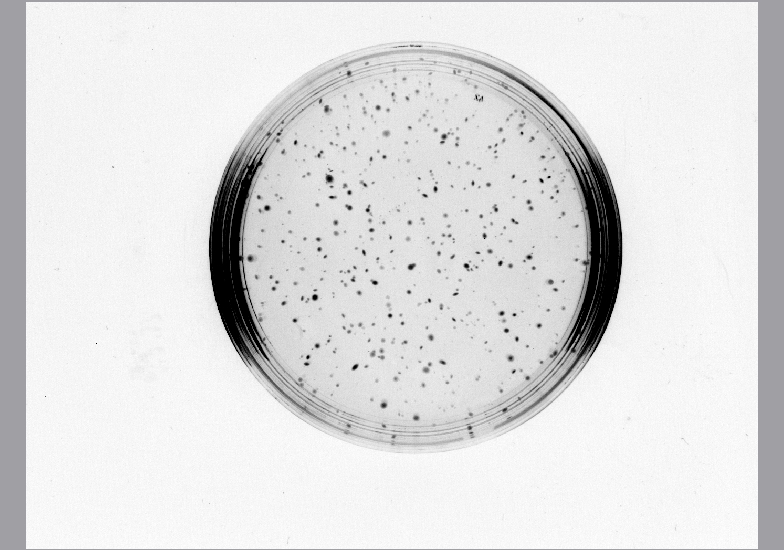

Supplement: Figure 1—figure supplement 1—source data 4. [file elife-95626-fig1-figsupp1-data4.zip › Raw images/Rep 1/A673_iefDBD+_R1_2.jpg]

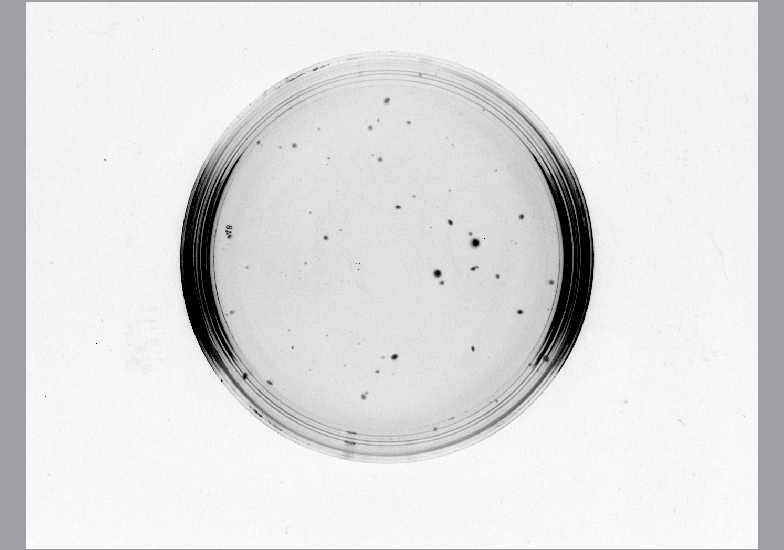

Supplement: Figure 1—figure supplement 1—source data 4. [file elife-95626-fig1-figsupp1-data4.zip › Raw images/Rep 1/A673_iefDBD_R1_1.jpg]

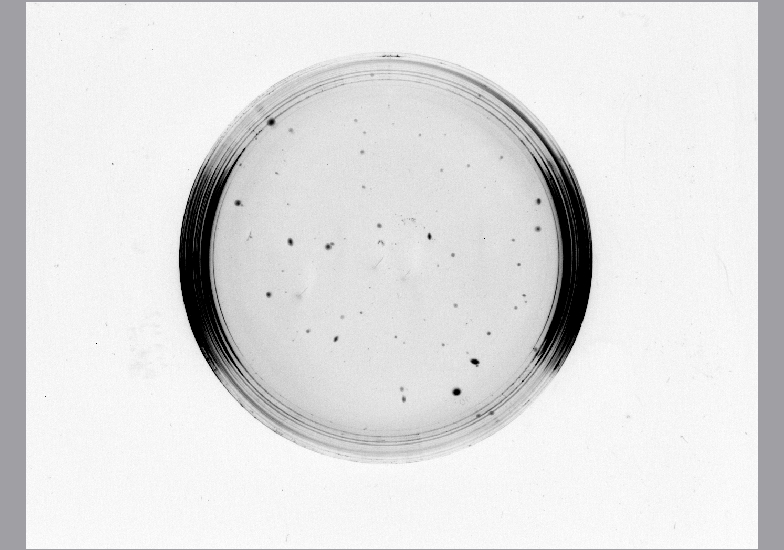

Supplement: Figure 1—figure supplement 1—source data 4. [file elife-95626-fig1-figsupp1-data4.zip › Raw images/Rep 1/A673_iefDBD_R1_2.jpg]

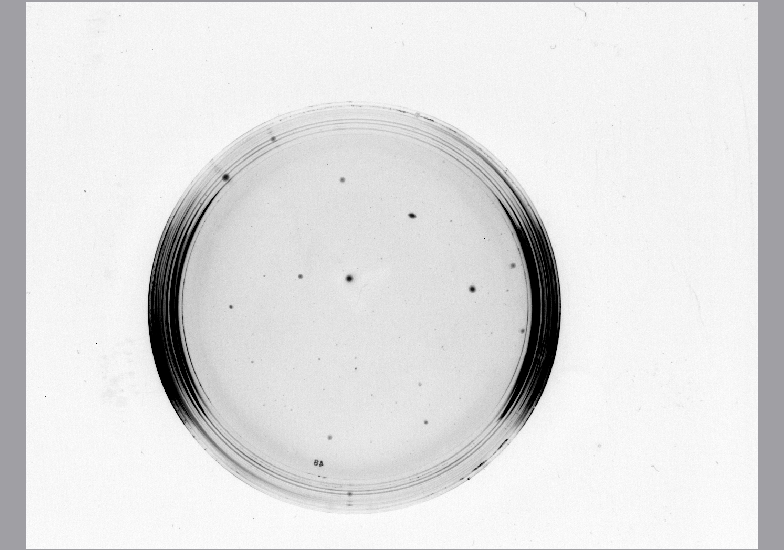

Supplement: Figure 1—figure supplement 1—source data 4. [file elife-95626-fig1-figsupp1-data4.zip › Raw images/Rep 2/A673_ief197_R2_1.jpg]

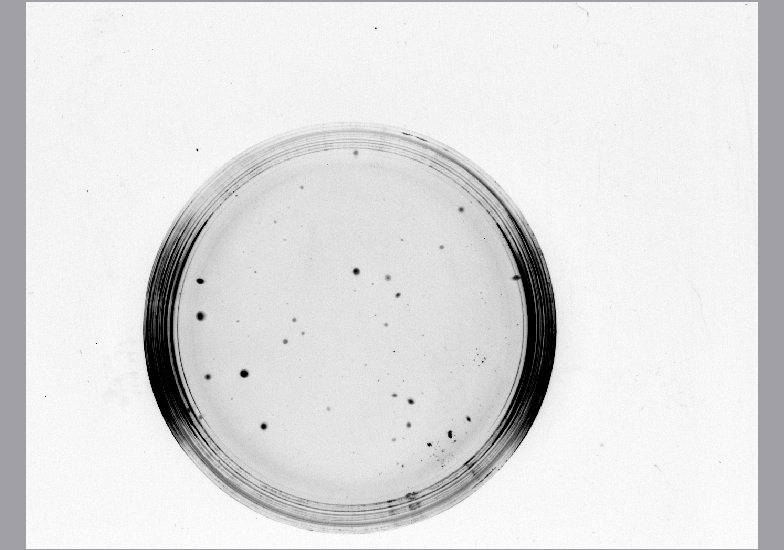

Supplement: Figure 1—figure supplement 1—source data 4. [file elife-95626-fig1-figsupp1-data4.zip › Raw images/Rep 2/A673_ief197_R2_2.jpg]

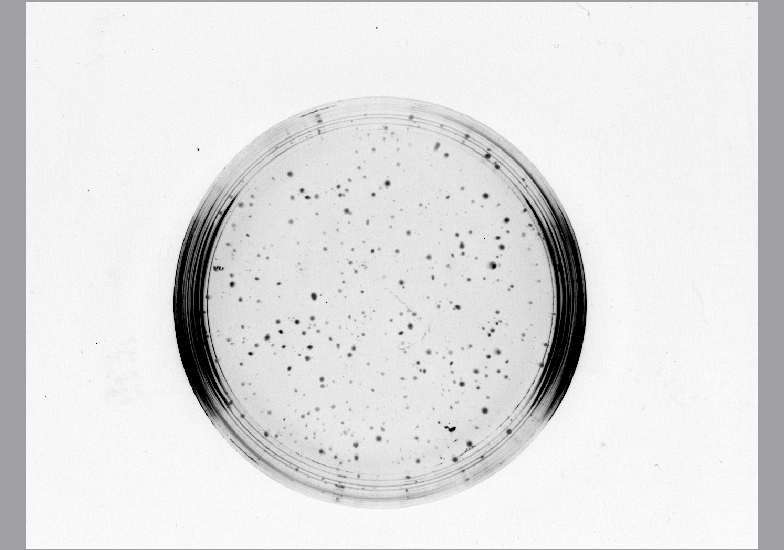

Supplement: Figure 1—figure supplement 1—source data 4. [file elife-95626-fig1-figsupp1-data4.zip › Raw images/Rep 2/A673_ief714_R2_1.jpg]

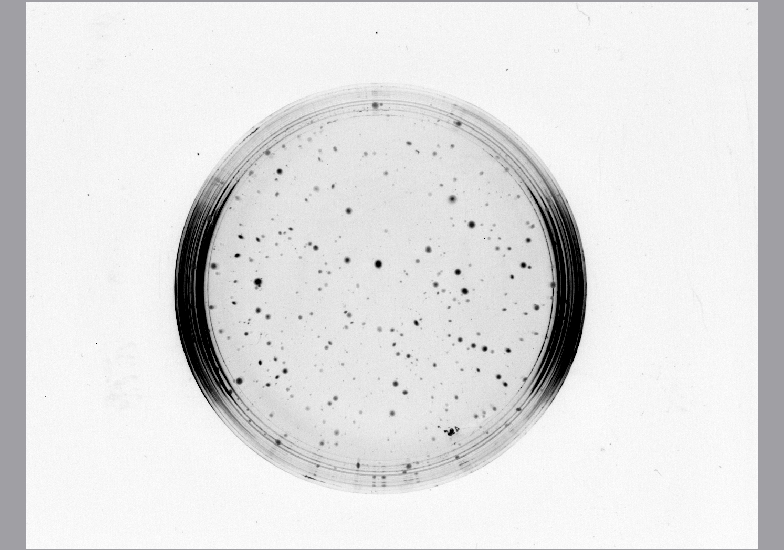

Supplement: Figure 1—figure supplement 1—source data 4. [file elife-95626-fig1-figsupp1-data4.zip › Raw images/Rep 2/A673_ief714_R2_2.jpg]

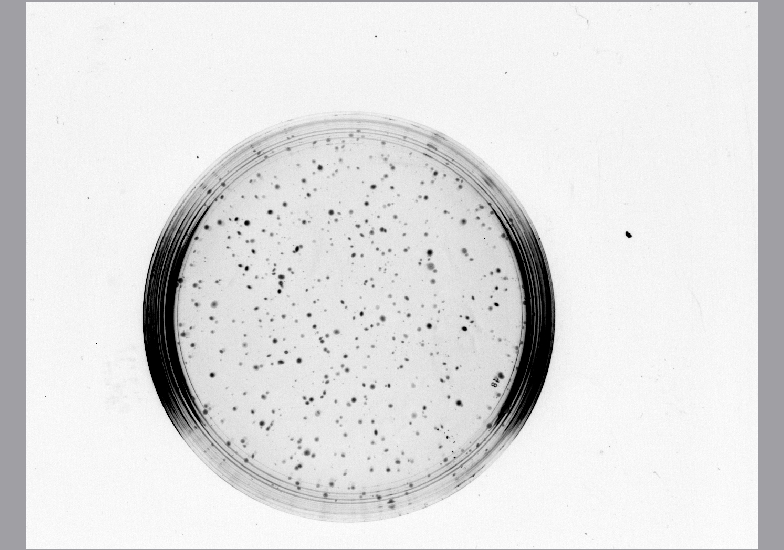

Supplement: Figure 1—figure supplement 1—source data 4. [file elife-95626-fig1-figsupp1-data4.zip › Raw images/Rep 2/A673_iefDBD+_R2_1.jpg]

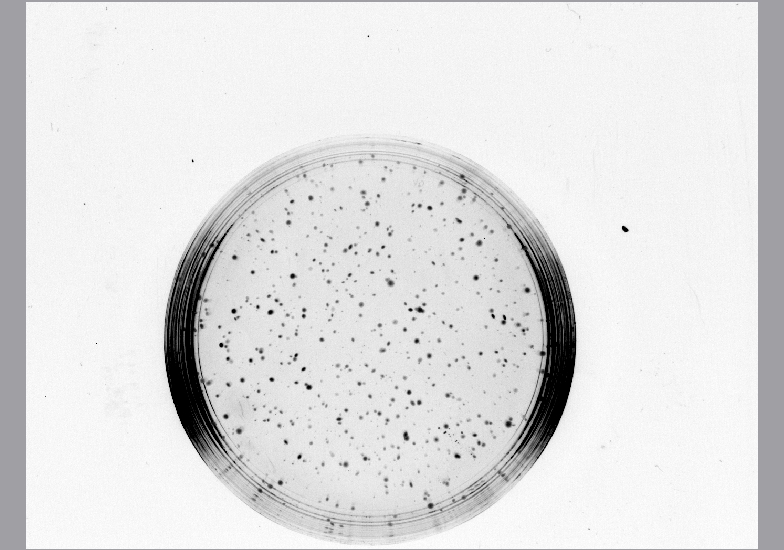

Supplement: Figure 1—figure supplement 1—source data 4. [file elife-95626-fig1-figsupp1-data4.zip › Raw images/Rep 2/A673_iefDBD+_R2_2.jpg]

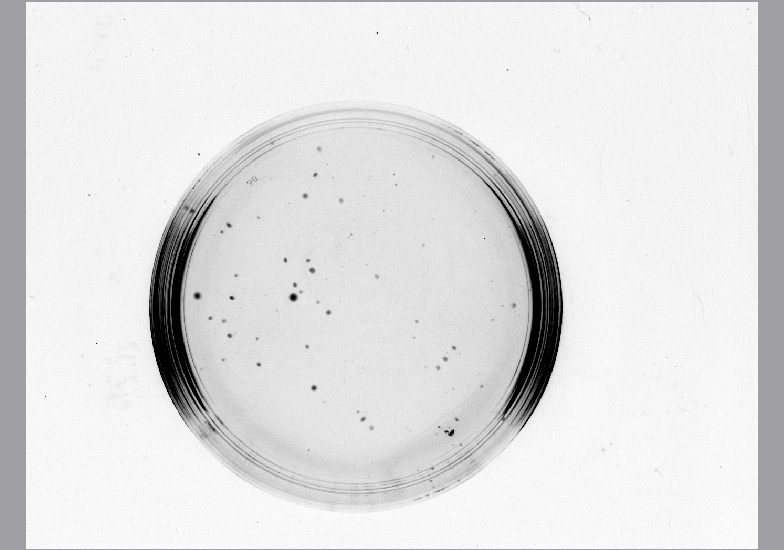

Supplement: Figure 1—figure supplement 1—source data 4. [file elife-95626-fig1-figsupp1-data4.zip › Raw images/Rep 2/A673_iefDBD_R2_1.jpg]

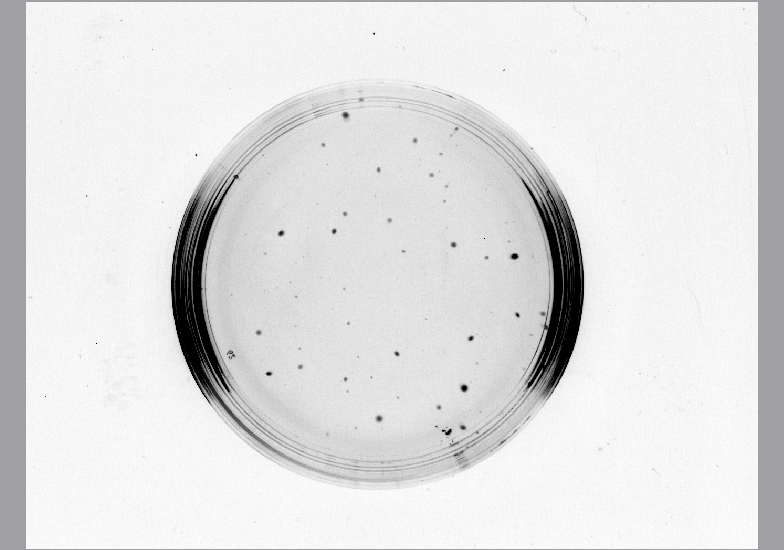

Supplement: Figure 1—figure supplement 1—source data 4. [file elife-95626-fig1-figsupp1-data4.zip › Raw images/Rep 2/A673_iefDBD_R2_2.jpg]

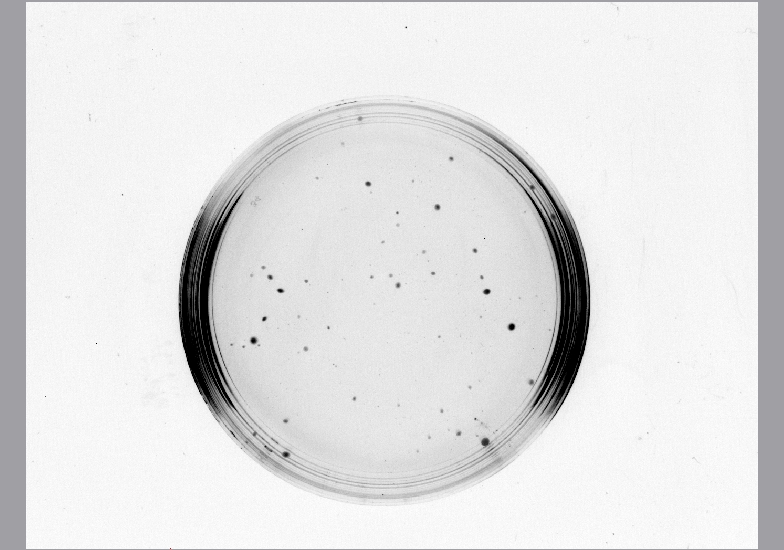

Supplement: Figure 1—figure supplement 1—source data 4. [file elife-95626-fig1-figsupp1-data4.zip › Raw images/Rep 3/A673_ief197_R3_1.jpg]

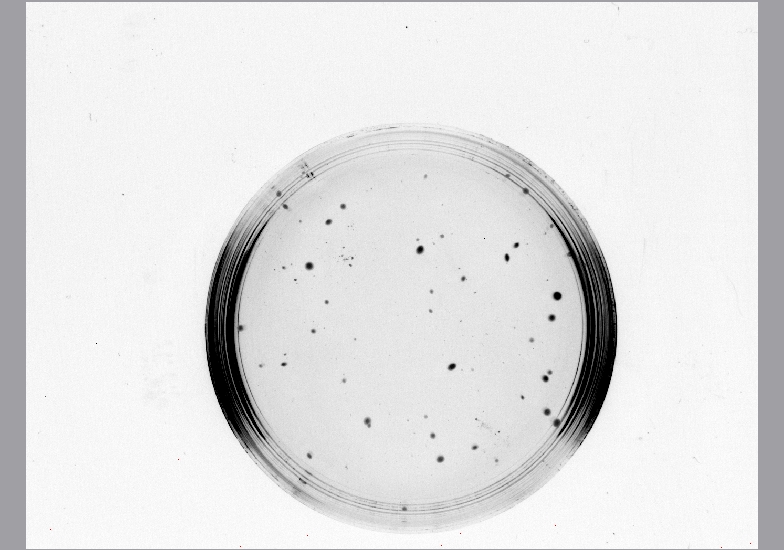

Supplement: Figure 1—figure supplement 1—source data 4. [file elife-95626-fig1-figsupp1-data4.zip › Raw images/Rep 3/A673_ief197_R3_2.jpg]

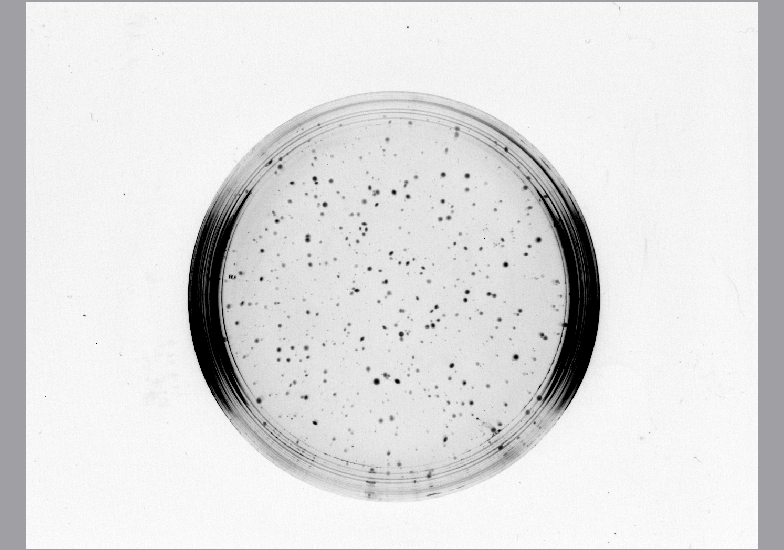

Supplement: Figure 1—figure supplement 1—source data 4. [file elife-95626-fig1-figsupp1-data4.zip › Raw images/Rep 3/A673_ief714_R3_1.jpg]

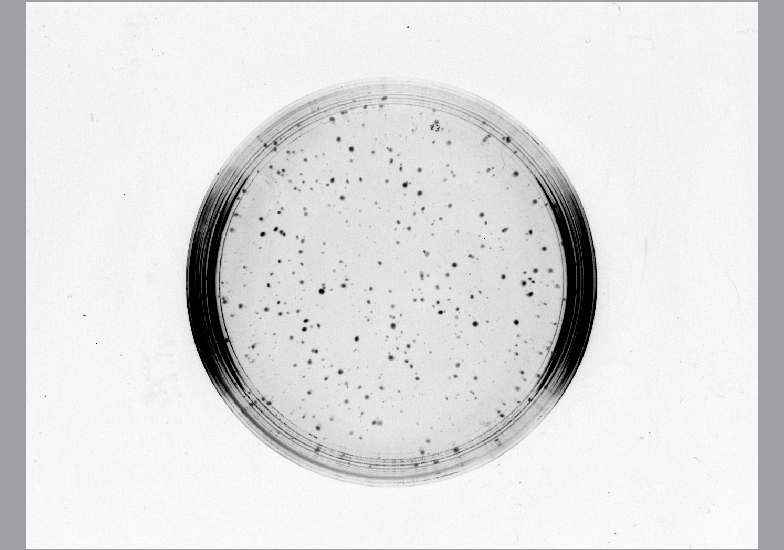

Supplement: Figure 1—figure supplement 1—source data 4. [file elife-95626-fig1-figsupp1-data4.zip › Raw images/Rep 3/A673_ief714_R3_2.jpg]

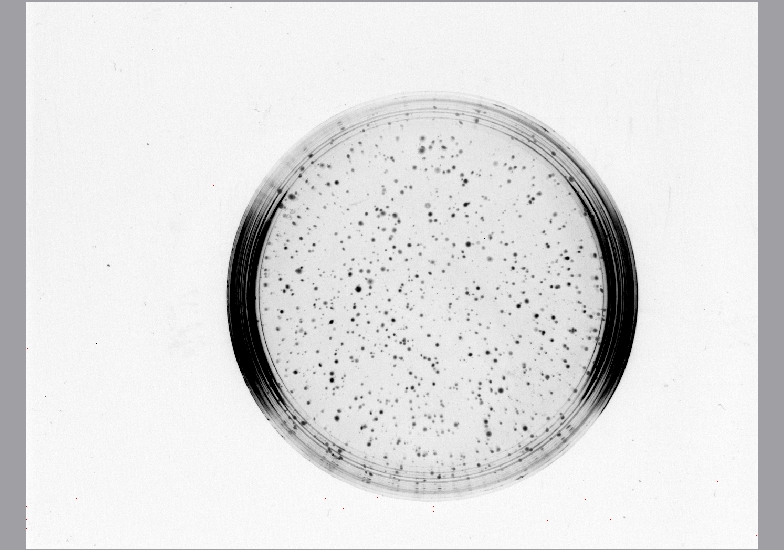

Supplement: Figure 1—figure supplement 1—source data 4. [file elife-95626-fig1-figsupp1-data4.zip › Raw images/Rep 3/A673_iefDBD+_R3_1.jpg]

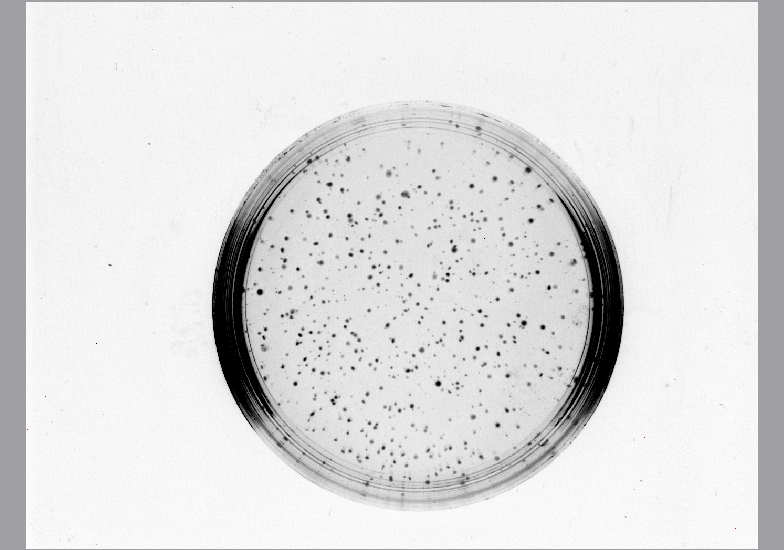

Supplement: Figure 1—figure supplement 1—source data 4. [file elife-95626-fig1-figsupp1-data4.zip › Raw images/Rep 3/A673_iefDBD+_R3_2.jpg]

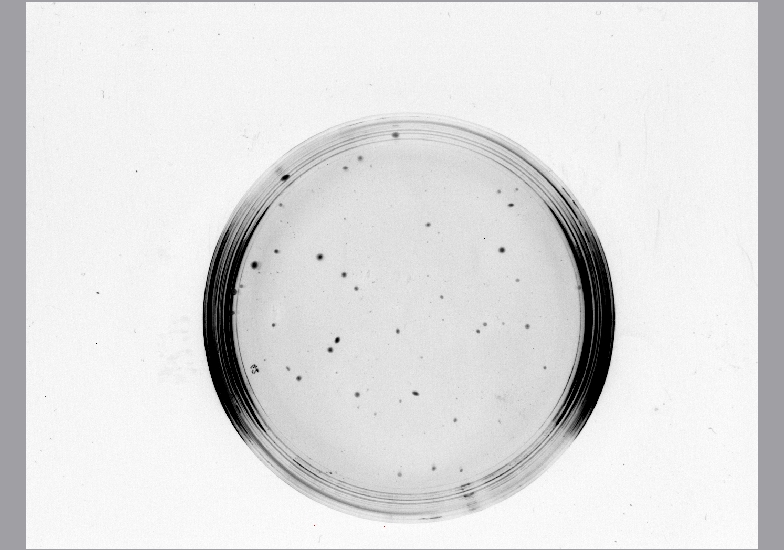

Supplement: Figure 1—figure supplement 1—source data 4. [file elife-95626-fig1-figsupp1-data4.zip › Raw images/Rep 3/A673_iefDBD_R3_1.jpg]

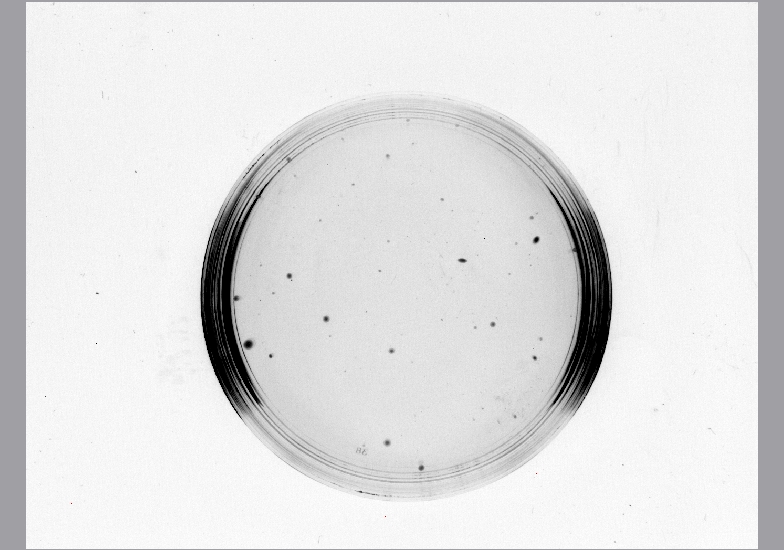

Supplement: Figure 1—figure supplement 1—source data 4. [file elife-95626-fig1-figsupp1-data4.zip › Raw images/Rep 3/A673_iefDBD_R3_2.jpg]

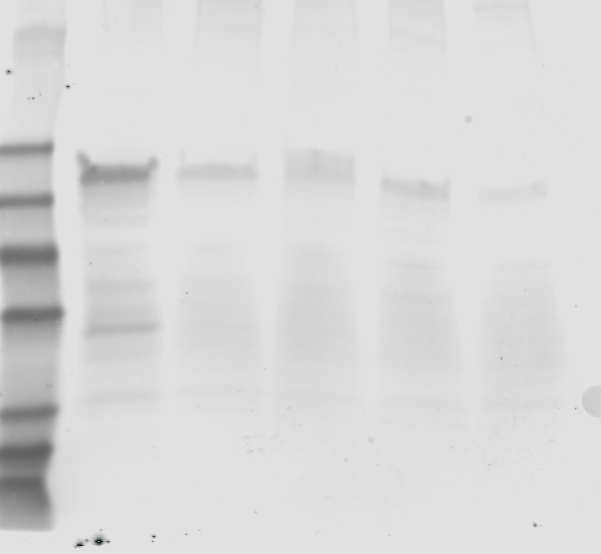

Supplement: Figure 1—figure supplement 3—source data 2. [file elife-95626-fig1-figsupp3-data2.zip › Raw images/erg_84um.jpg]

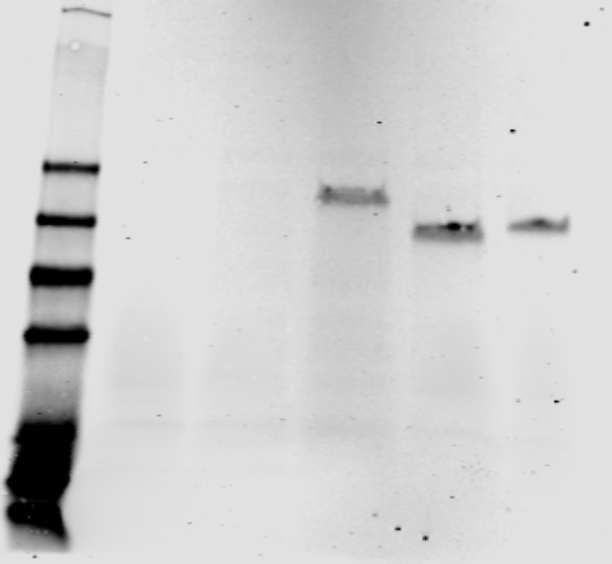

Supplement: Figure 1—figure supplement 3—source data 2. [file elife-95626-fig1-figsupp3-data2.zip › Raw images/Flag_84um.jpg]

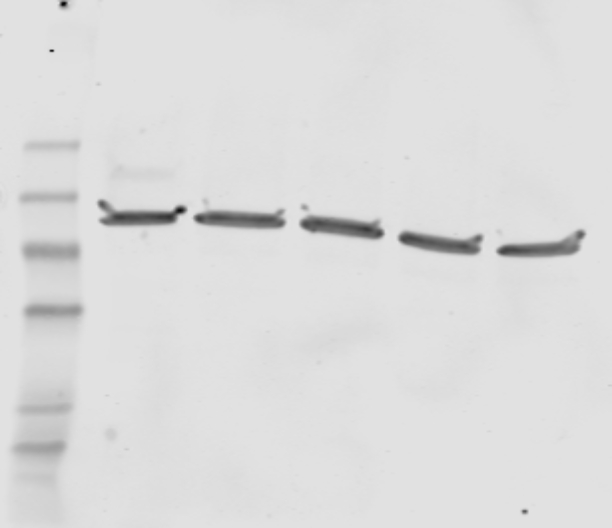

Supplement: Figure 1—figure supplement 3—source data 2. [file elife-95626-fig1-figsupp3-data2.zip › Raw images/tubulin_169um.jpg]

iERG/197

iERG/wtEF

iERG/DBD

iERG/DBD+

Tech rep 1

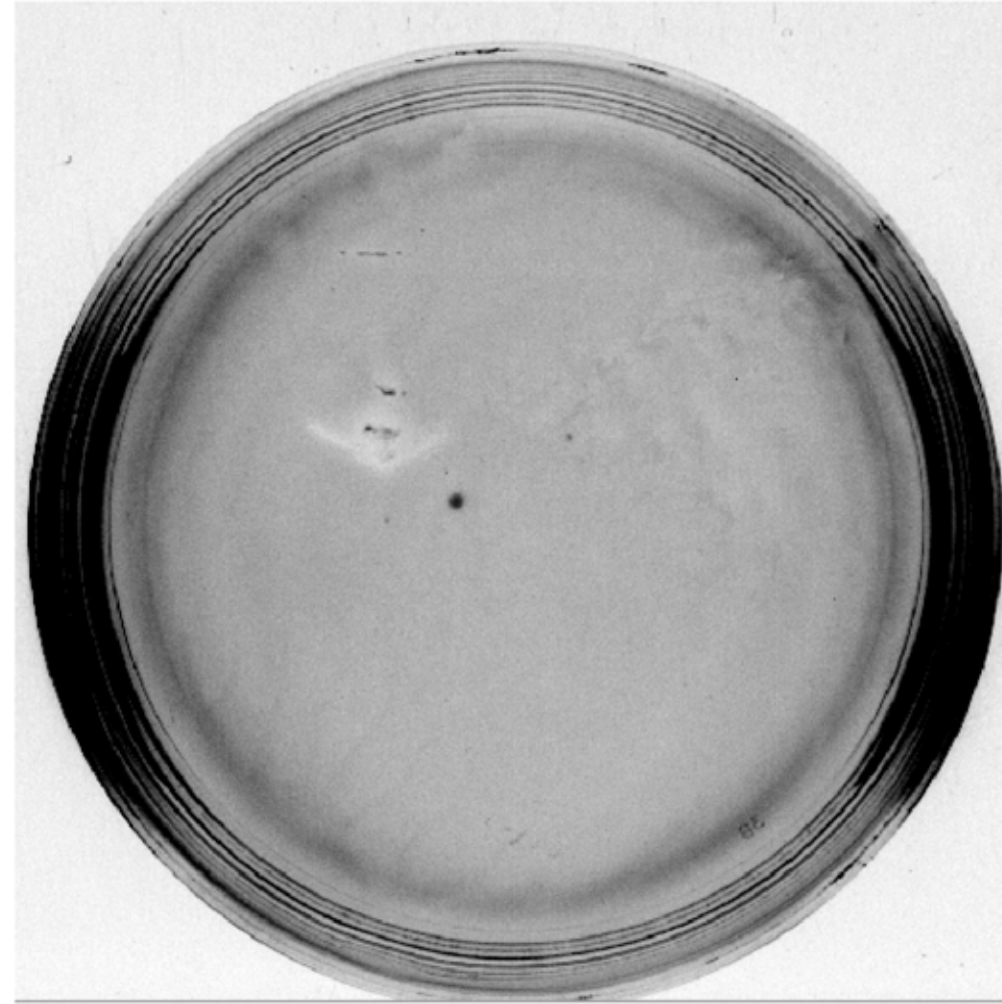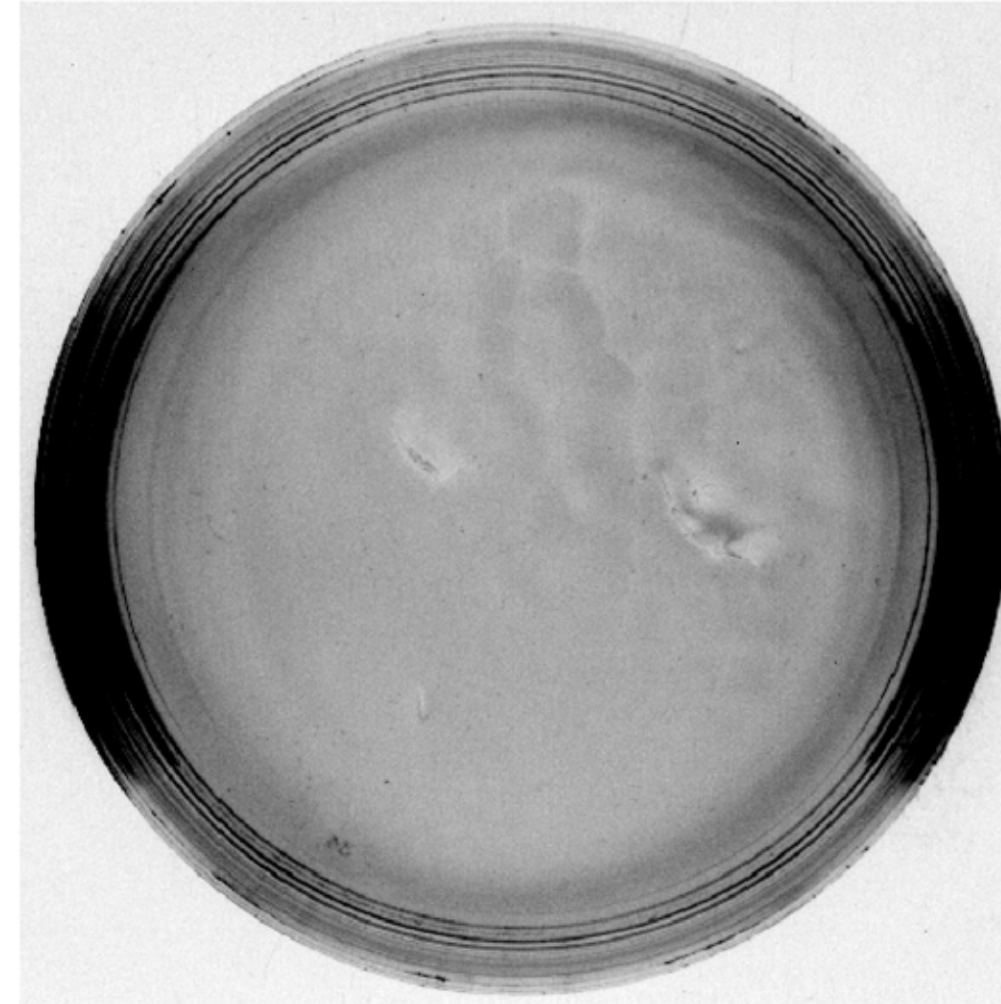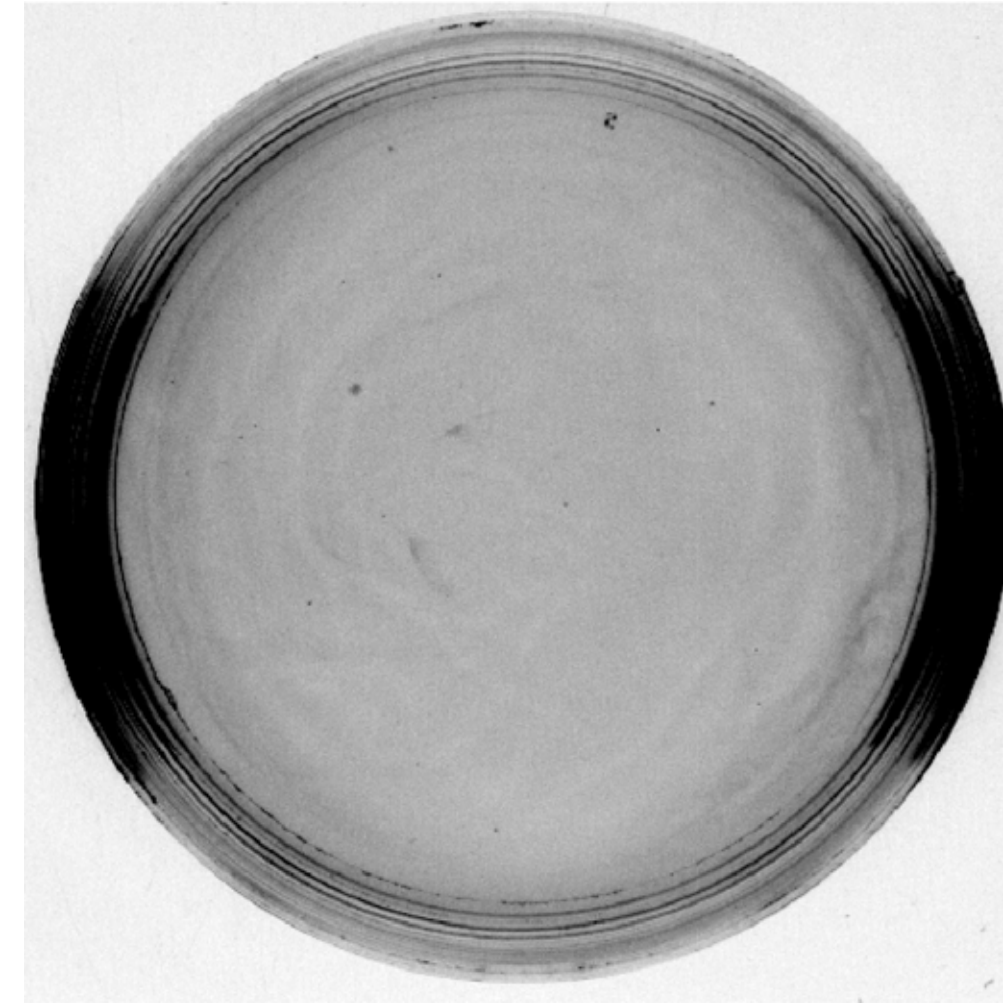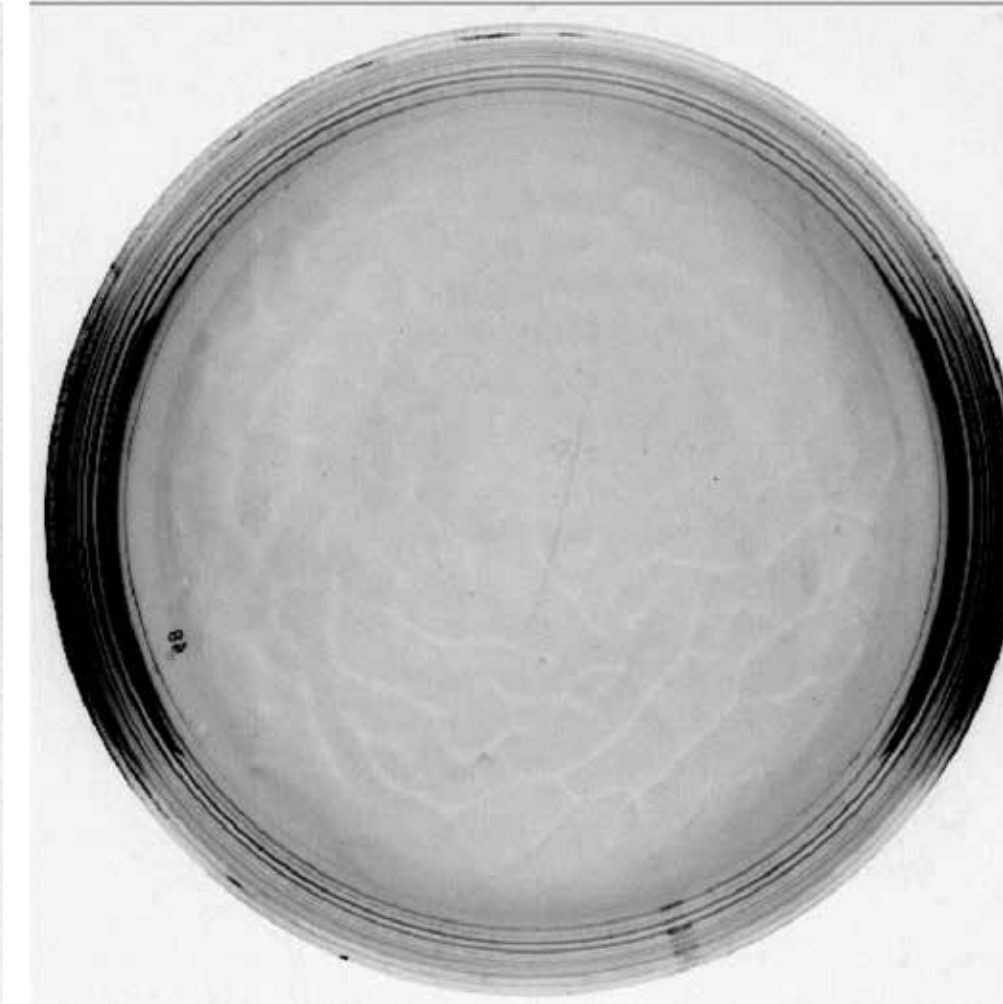

Tech rep 2

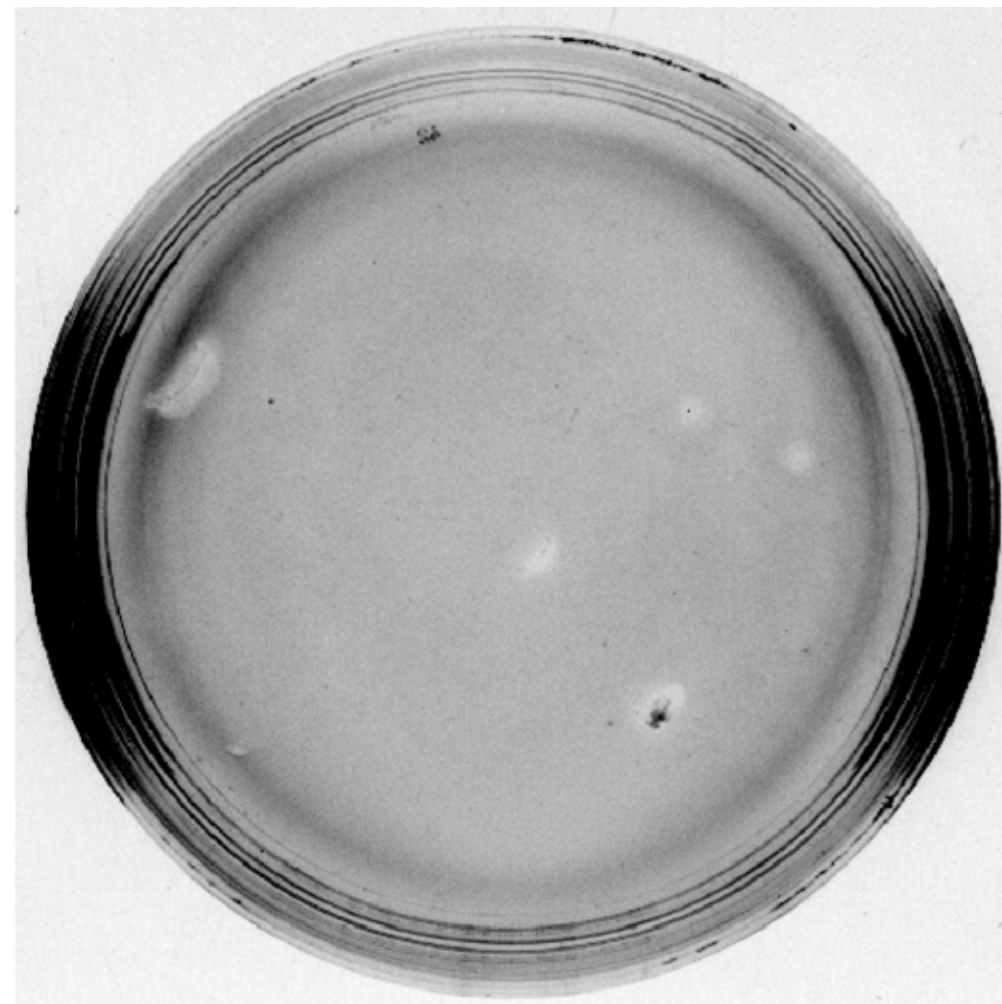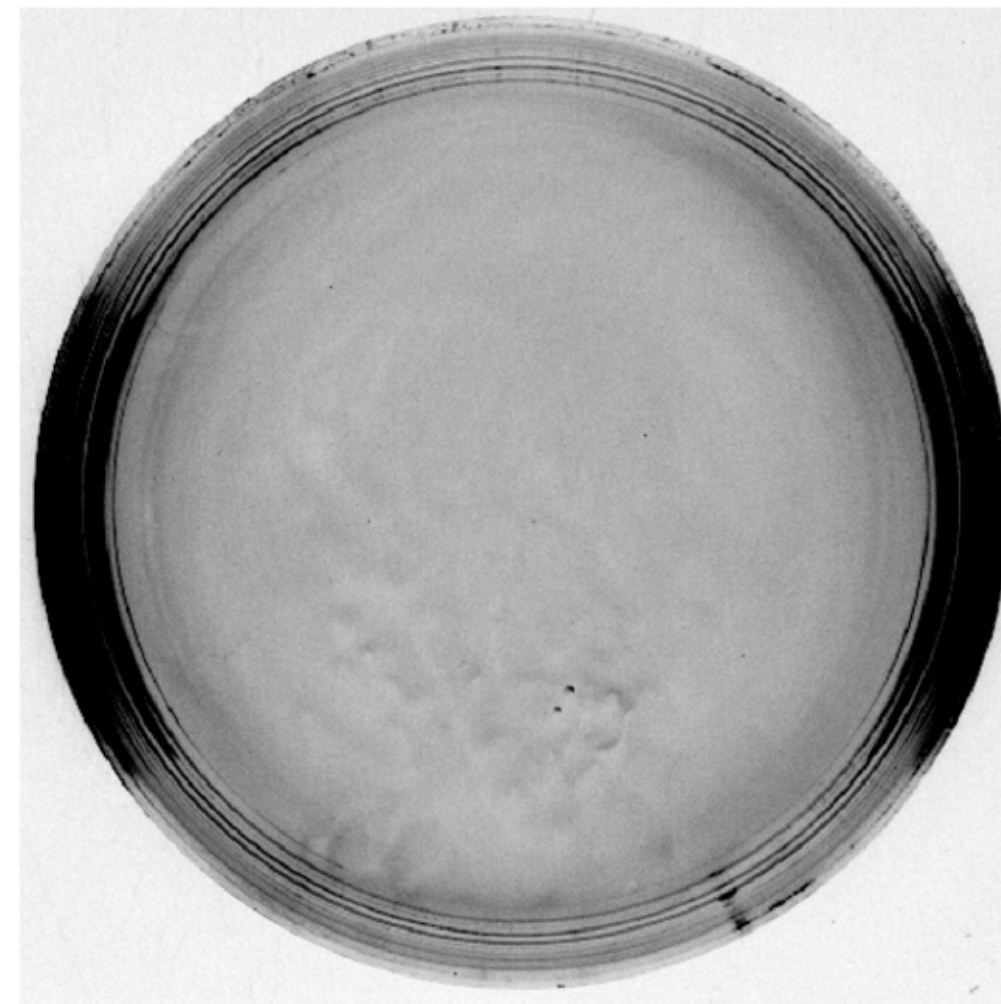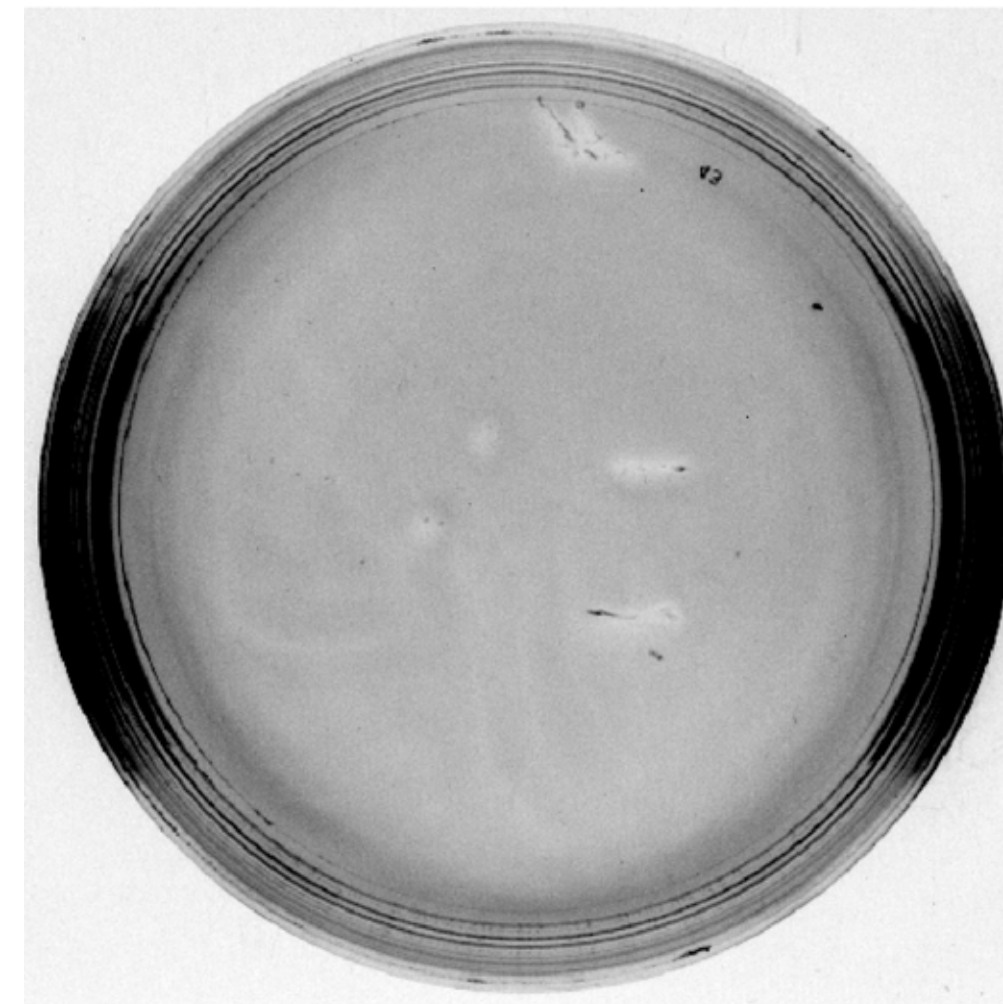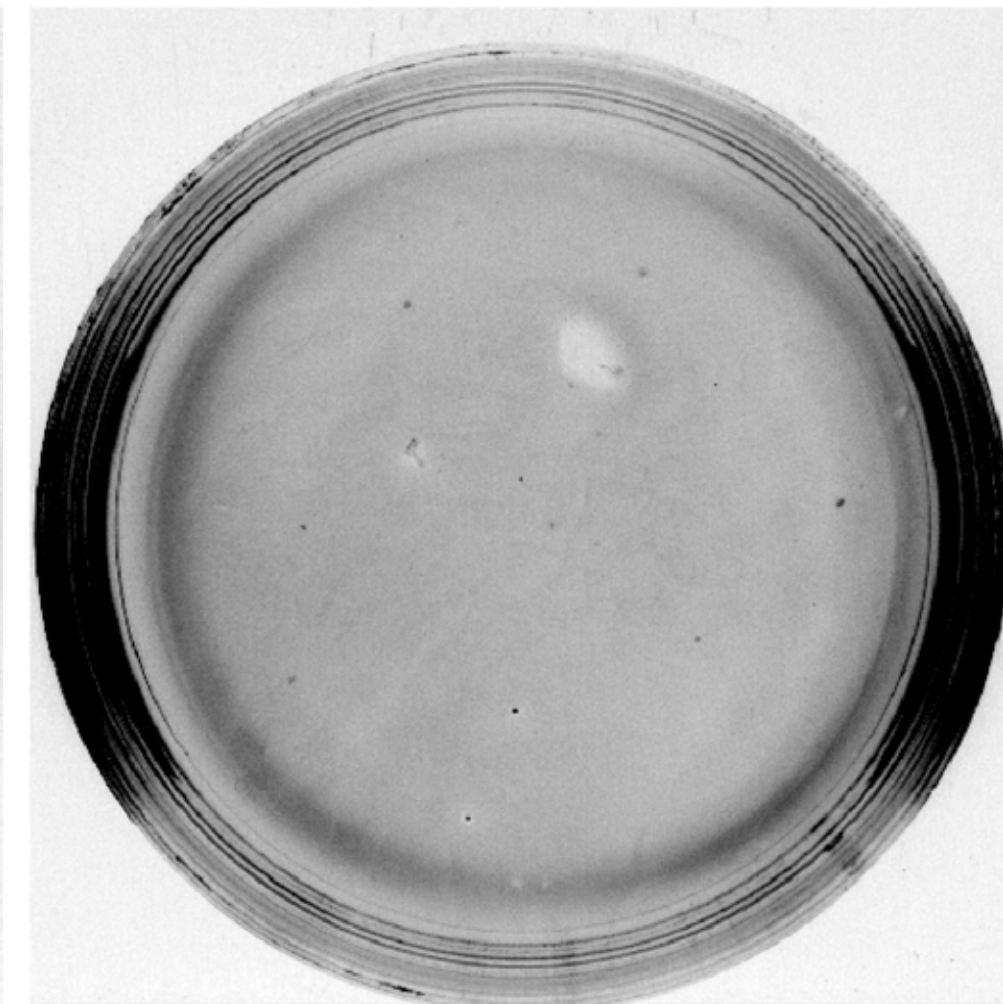

TTC466 Rep 2

Supplement: Figure 1—figure supplement 3—source data 3. [file elife-95626-fig1-figsupp3-data3.zip › Labelled images/R2_submission.pdf]

iERG/197

iERG/wtEF

iERG/DBD

iERG/DBD+

Tech rep 1

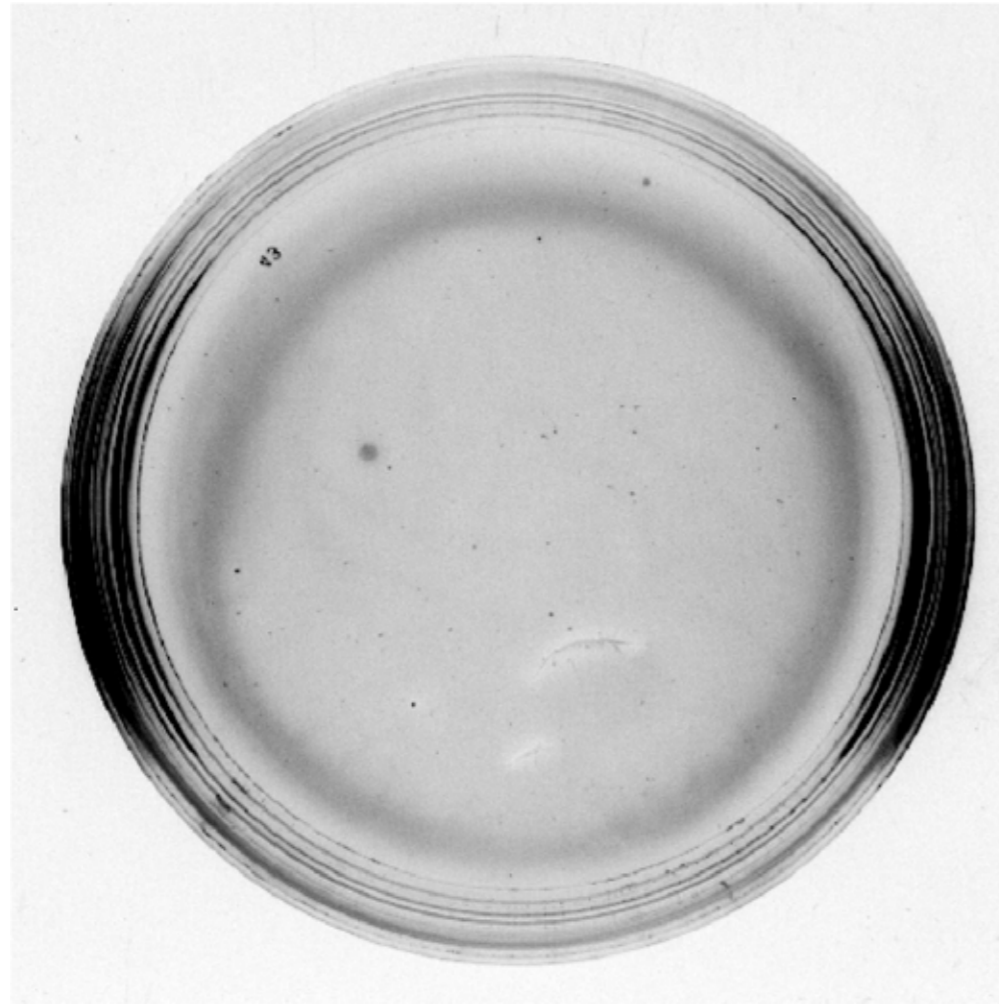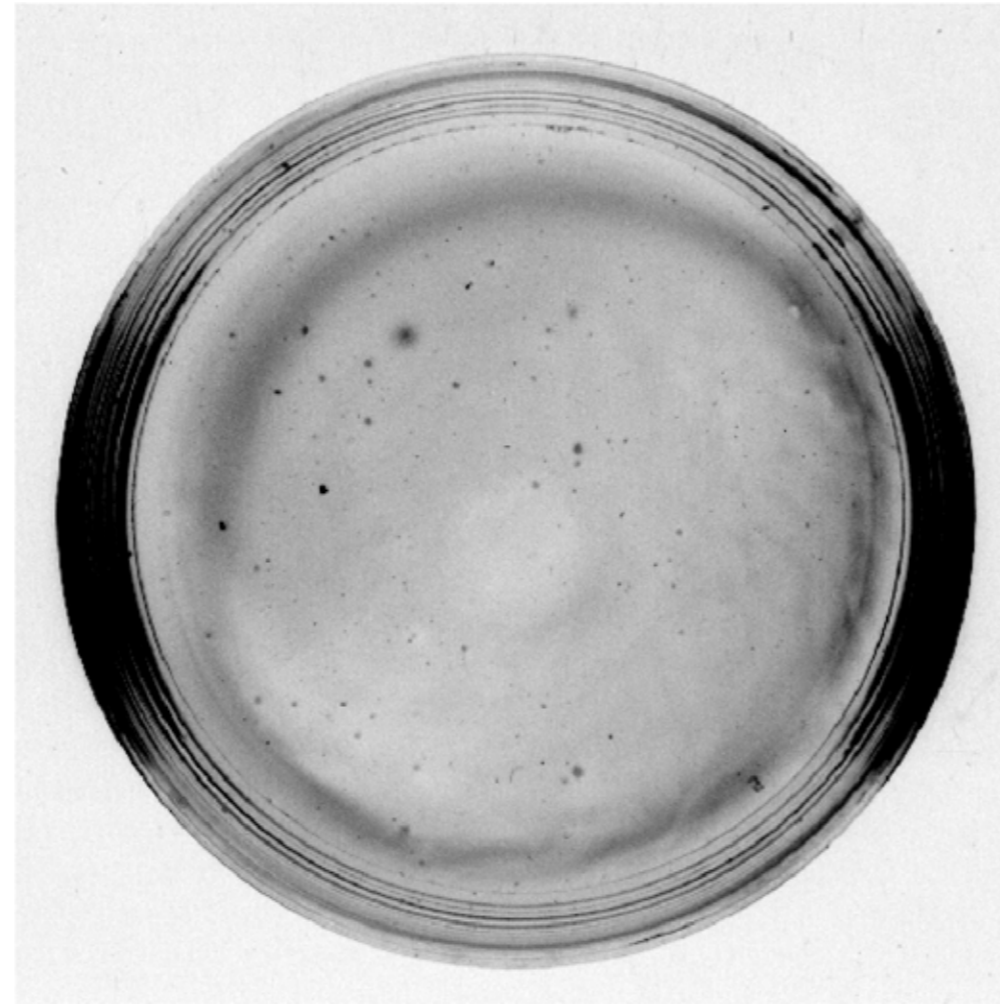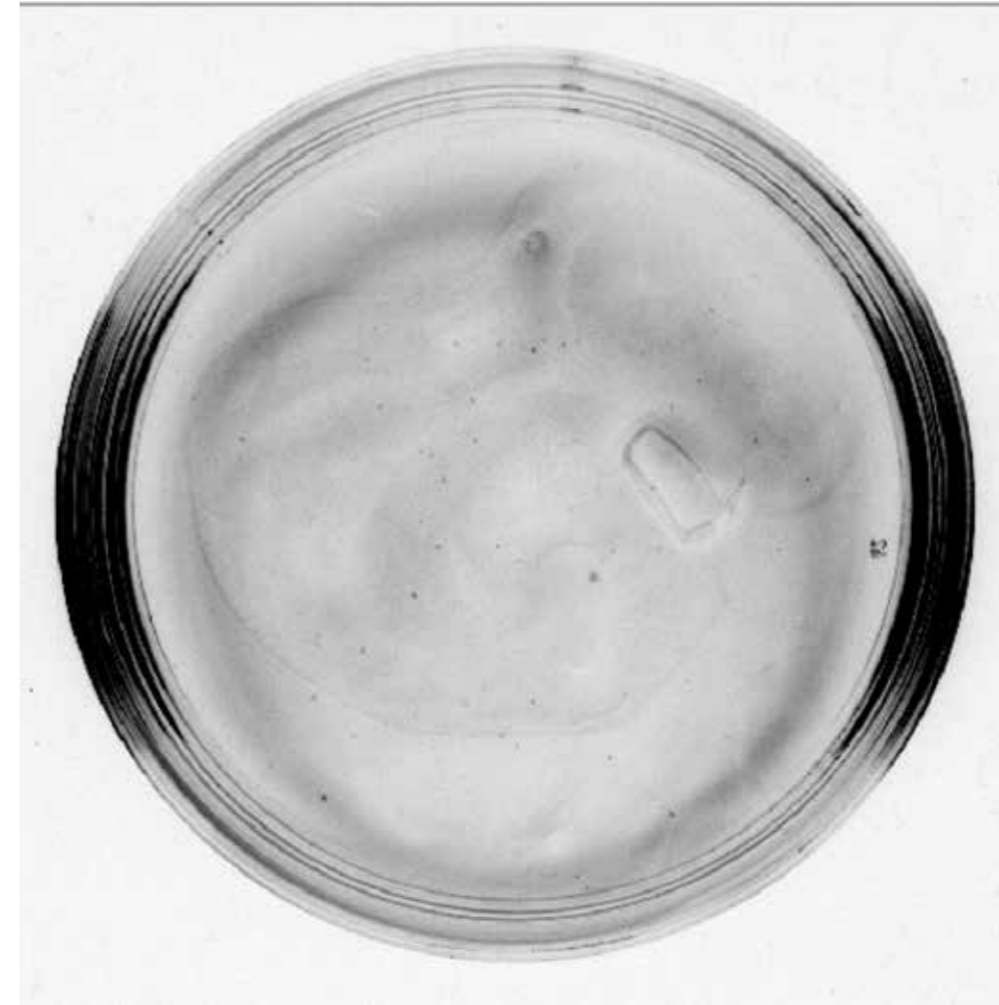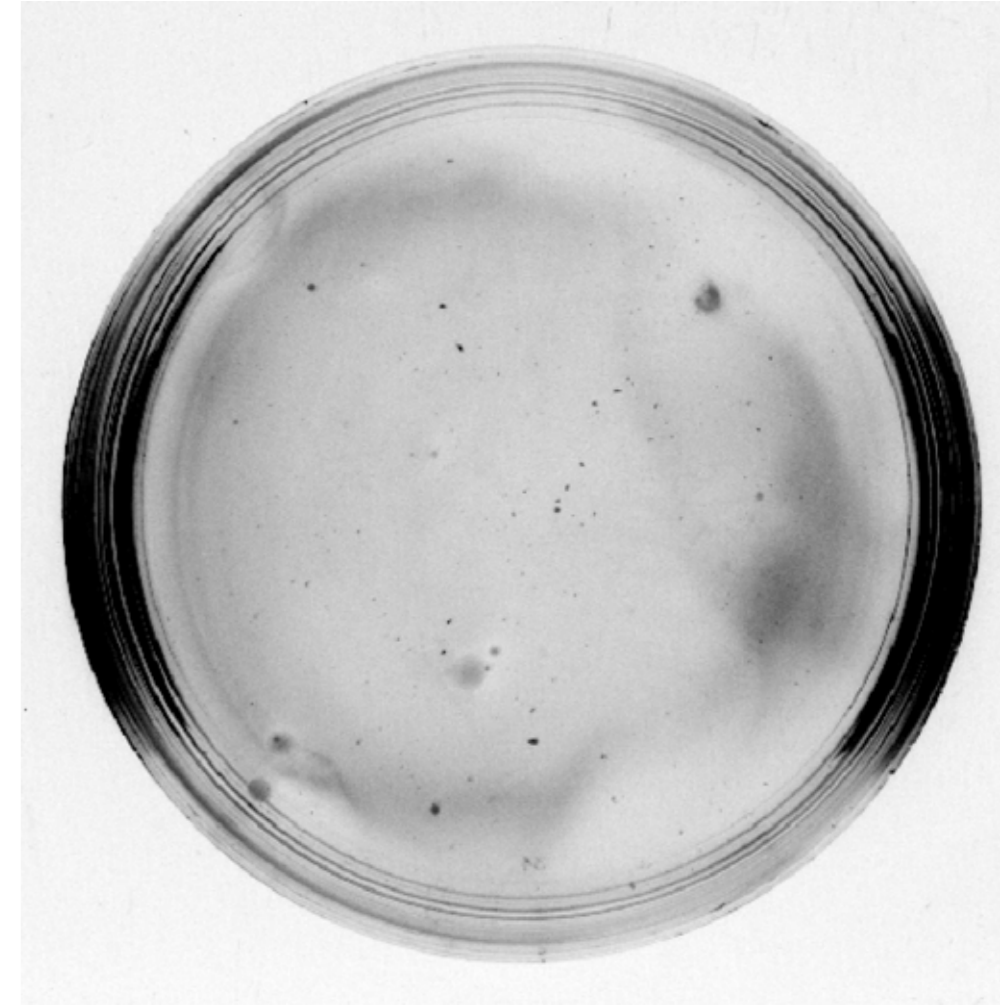

Tech rep 2

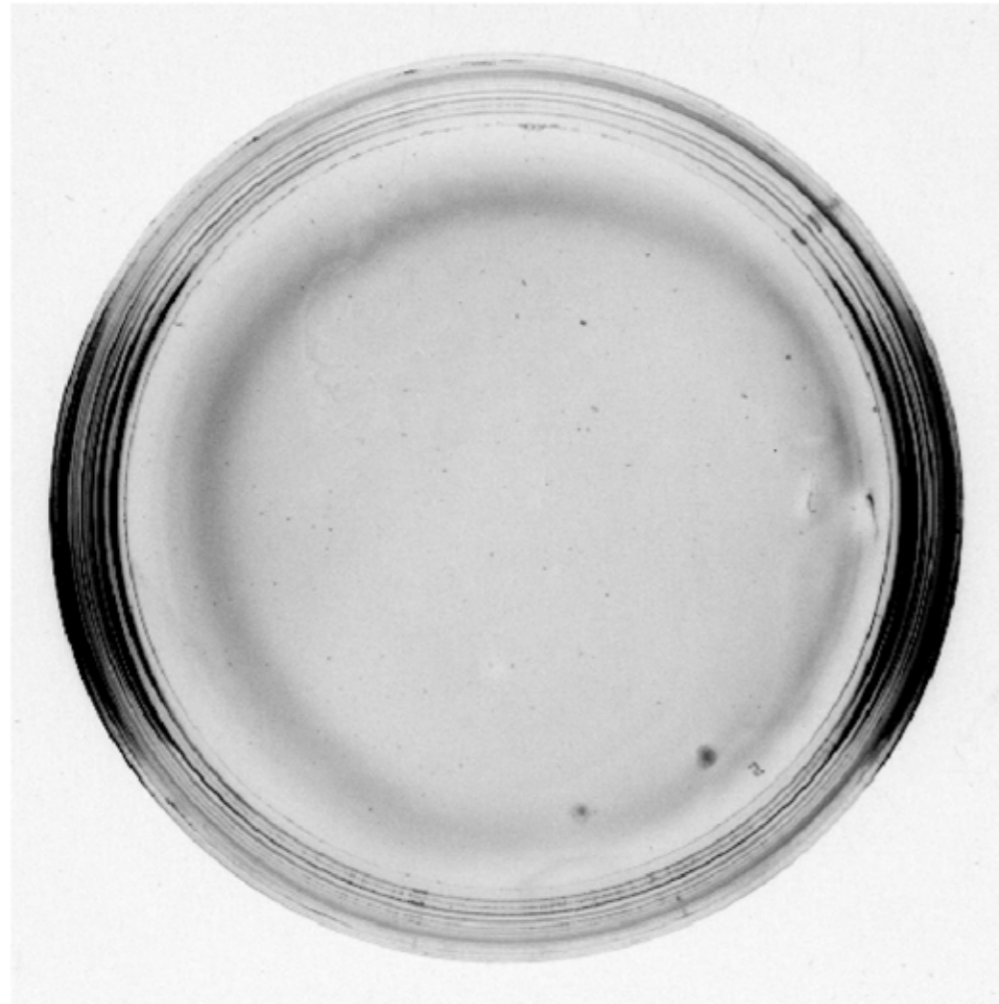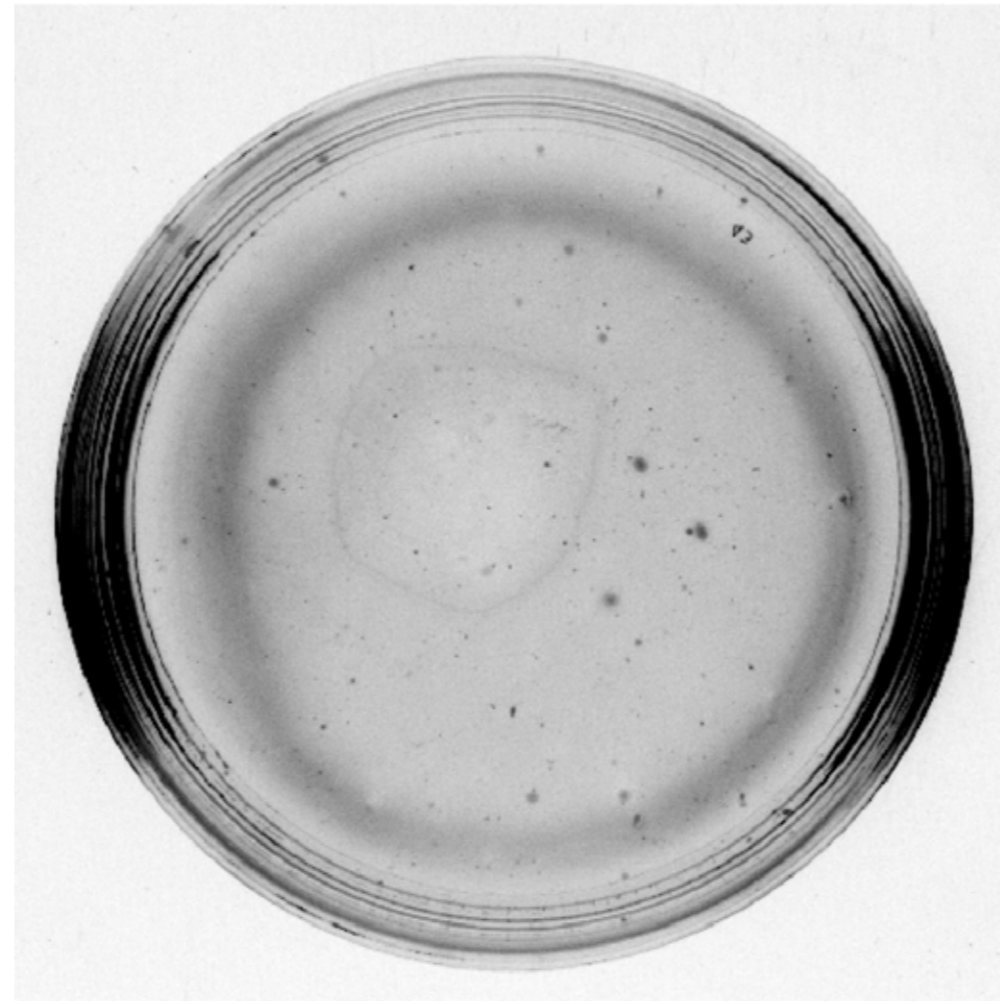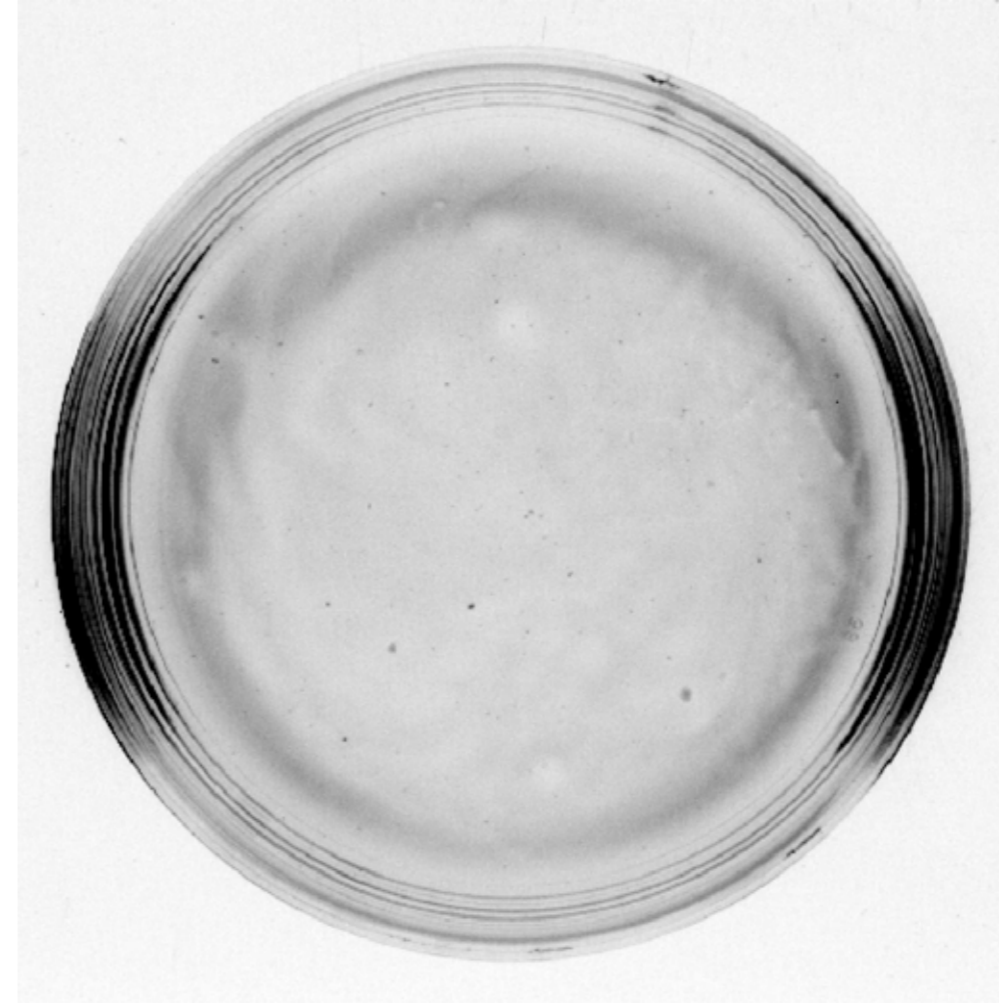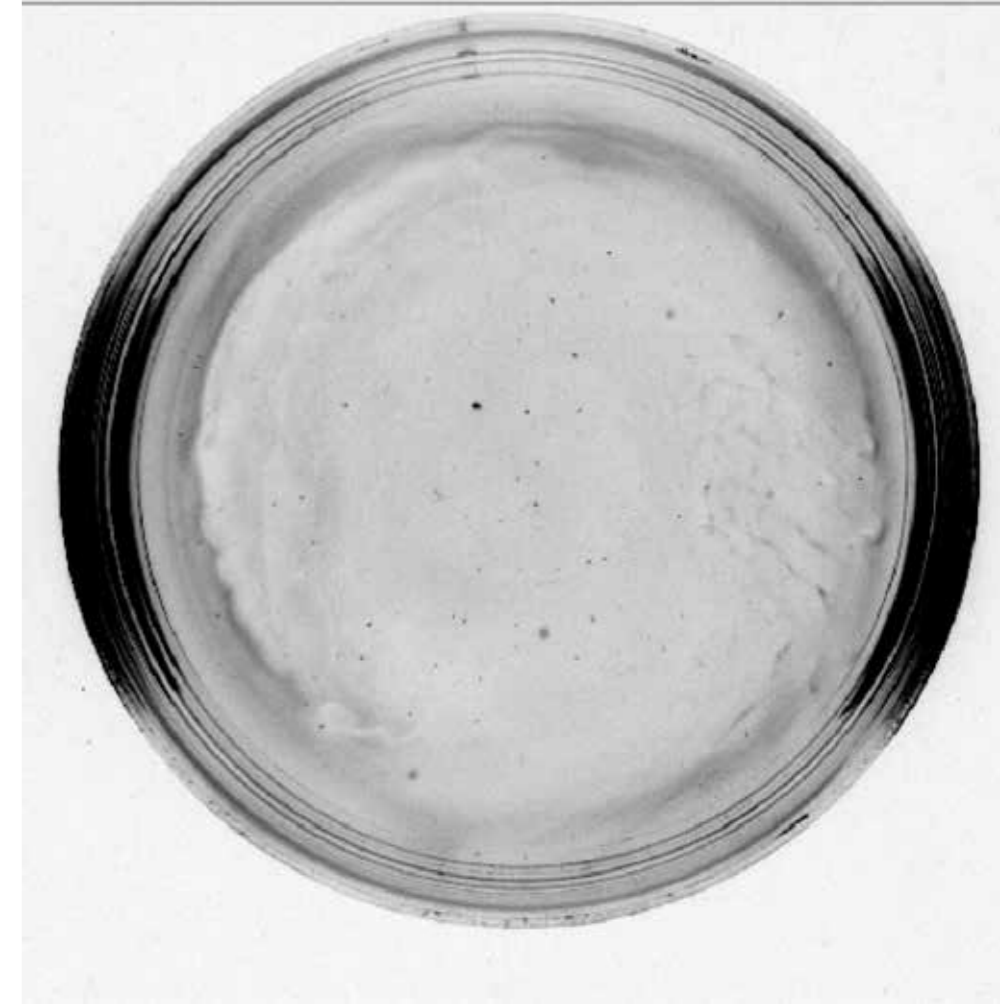

TTC466 Rep 3

Supplement: Figure 1—figure supplement 3—source data 3. [file elife-95626-fig1-figsupp3-data3.zip › Labelled images/R3_submission.pdf]

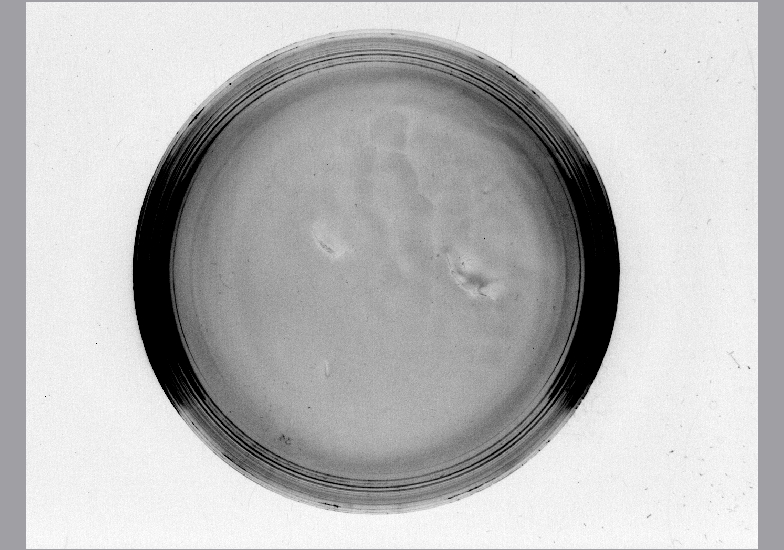

Supplement: Figure 1—figure supplement 3—source data 4. [file elife-95626-fig1-figsupp3-data4.zip › Raw images/Rep 2/TTC466_Rep2_714_1.jpg]

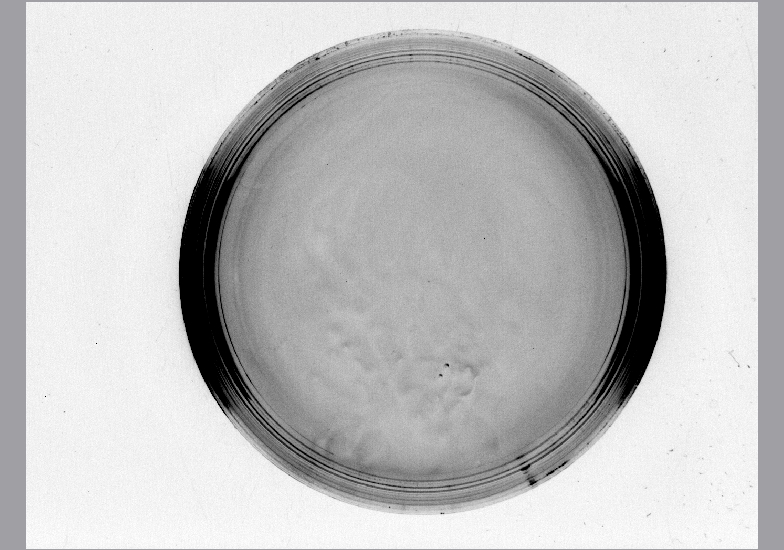

Supplement: Figure 1—figure supplement 3—source data 4. [file elife-95626-fig1-figsupp3-data4.zip › Raw images/Rep 2/TTC466_Rep2_714_2.jpg]

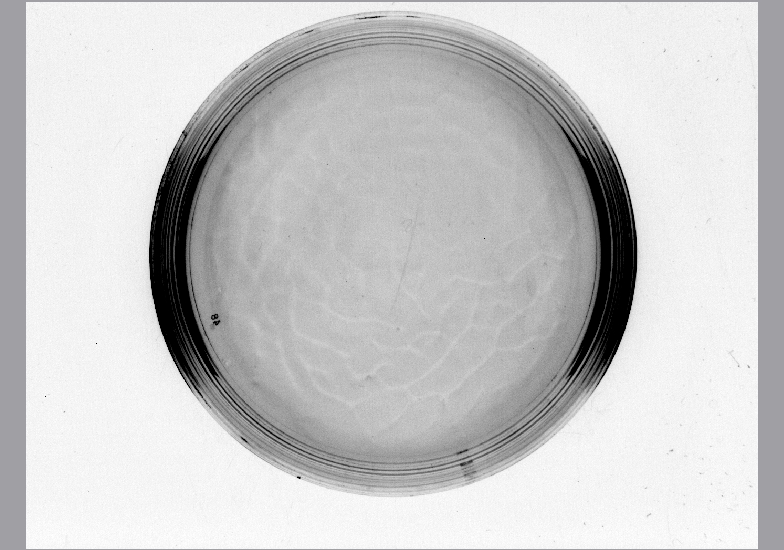

Supplement: Figure 1—figure supplement 3—source data 4. [file elife-95626-fig1-figsupp3-data4.zip › Raw images/Rep 2/TTC466_Rep2_714_DBD+_1.jpg]

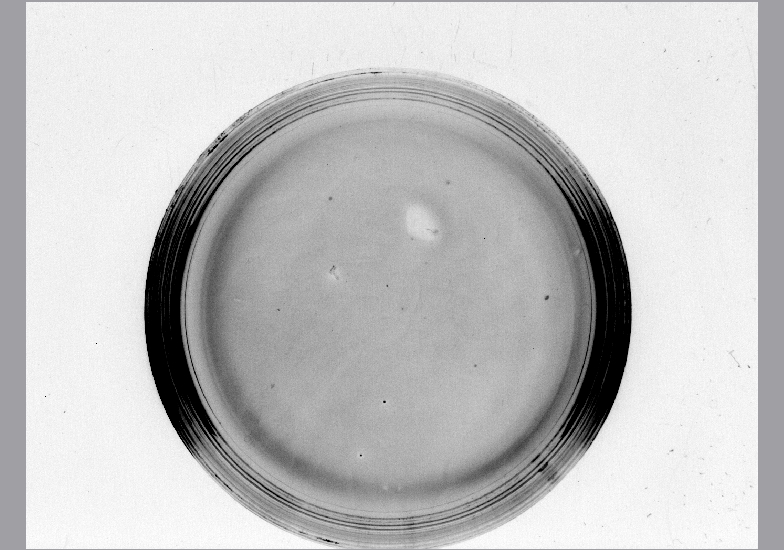

Supplement: Figure 1—figure supplement 3—source data 4. [file elife-95626-fig1-figsupp3-data4.zip › Raw images/Rep 2/TTC466_Rep2_714_DBD+_2.jpg]

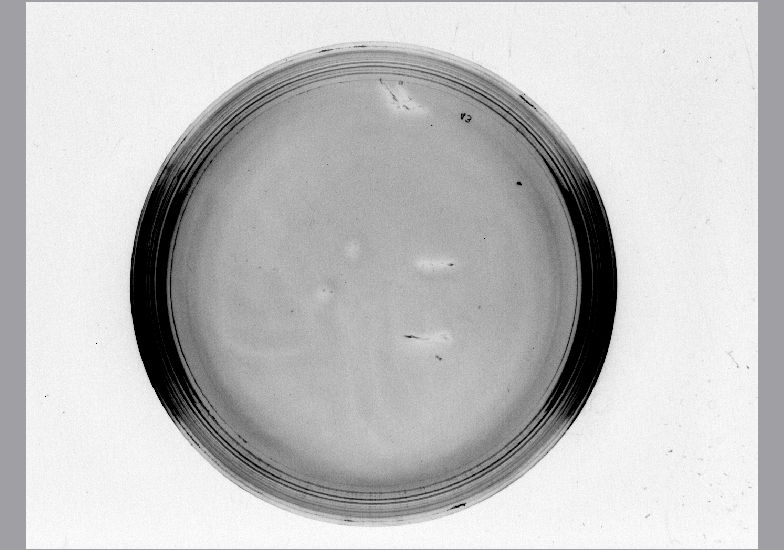

Supplement: Figure 1—figure supplement 3—source data 4. [file elife-95626-fig1-figsupp3-data4.zip › Raw images/Rep 2/TTC466_Rep2_714_DBD.jpg]

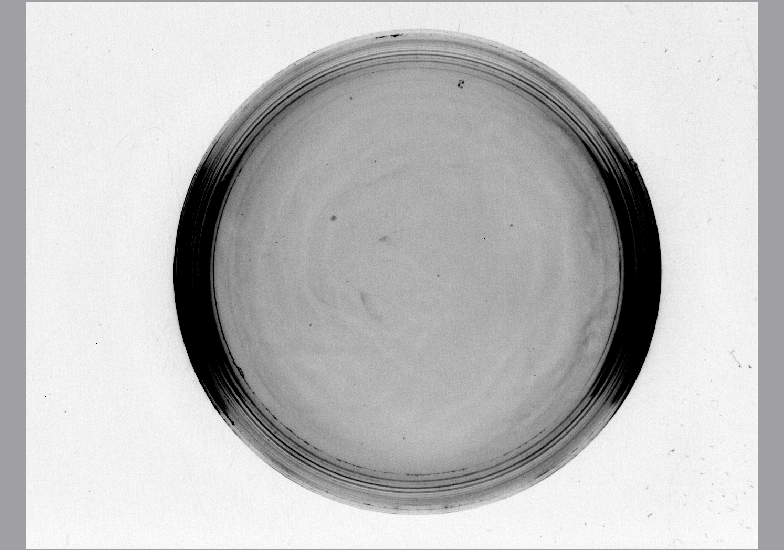

Supplement: Figure 1—figure supplement 3—source data 4. [file elife-95626-fig1-figsupp3-data4.zip › Raw images/Rep 2/TTC466_Rep2_714_DBD_2.jpg]

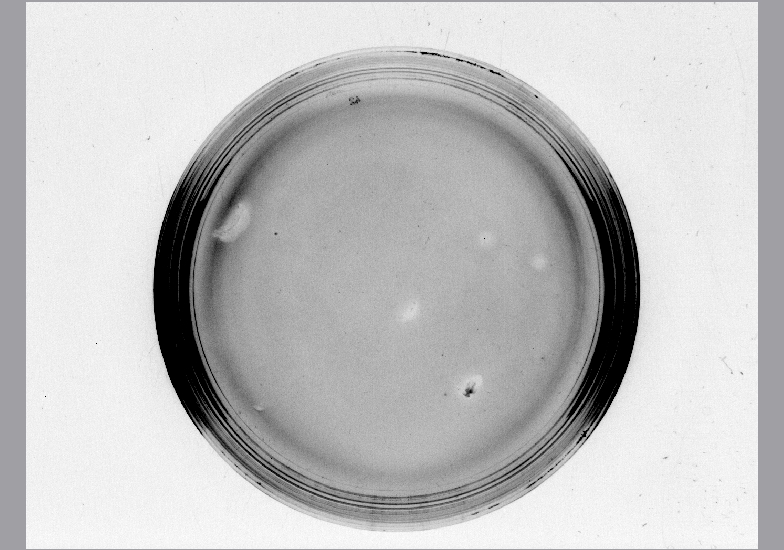

Supplement: Figure 1—figure supplement 3—source data 4. [file elife-95626-fig1-figsupp3-data4.zip › Raw images/Rep 2/TTC466_Rep2_iERG_1.jpg]

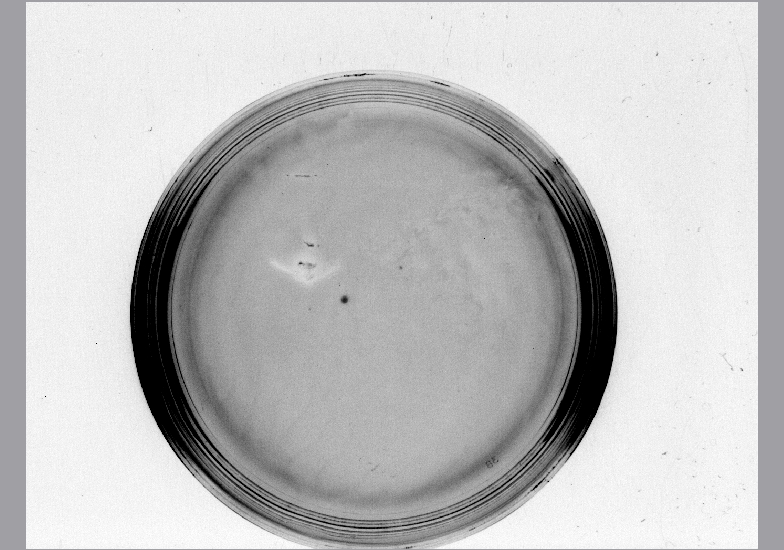

Supplement: Figure 1—figure supplement 3—source data 4. [file elife-95626-fig1-figsupp3-data4.zip › Raw images/Rep 2/TTC466_Rep2_iERG_2.jpg]

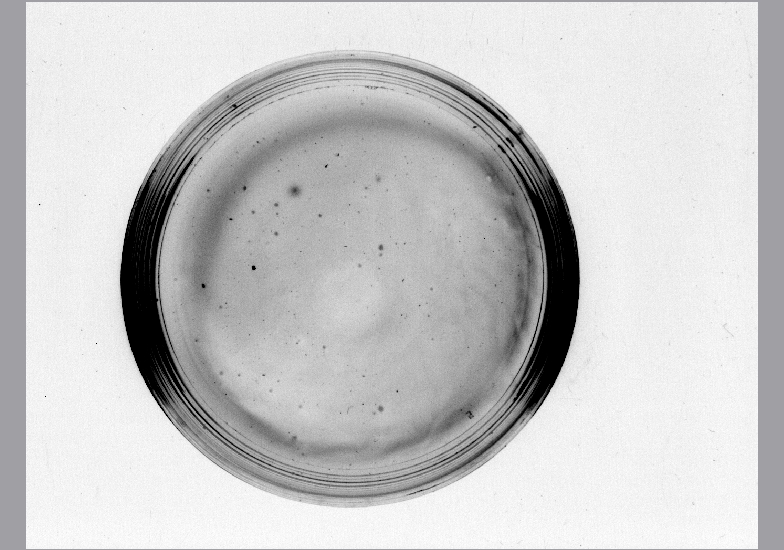

Supplement: Figure 1—figure supplement 3—source data 4. [file elife-95626-fig1-figsupp3-data4.zip › Raw images/Rep 3/TTC446_Rep3_714_1.jpg]

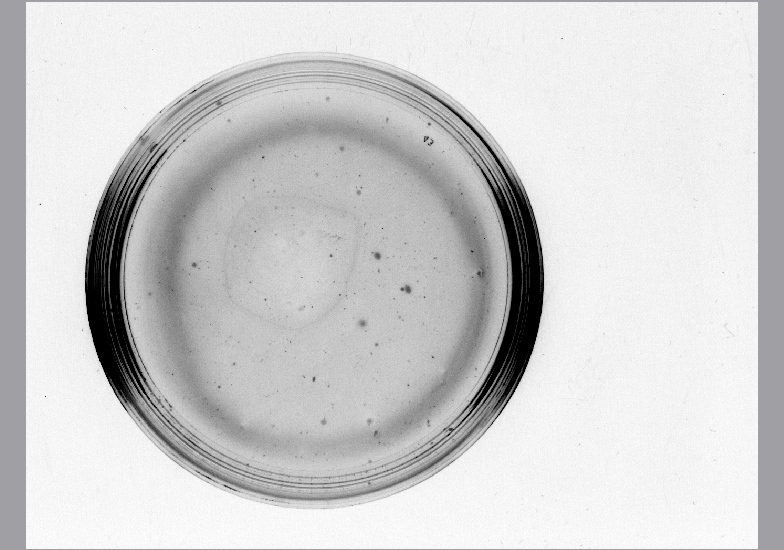

Supplement: Figure 1—figure supplement 3—source data 4. [file elife-95626-fig1-figsupp3-data4.zip › Raw images/Rep 3/TTC446_Rep3_714_2.jpg]

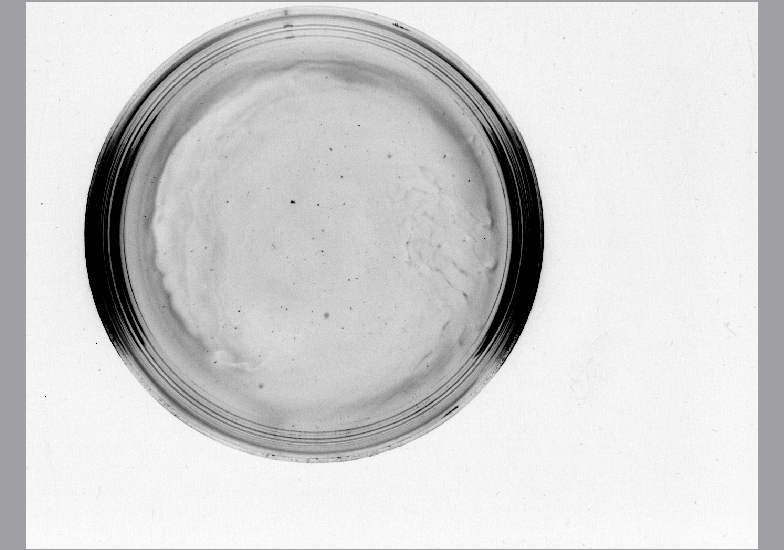

Supplement: Figure 1—figure supplement 3—source data 4. [file elife-95626-fig1-figsupp3-data4.zip › Raw images/Rep 3/TTC446_Rep3_DBD+_1.jpg]

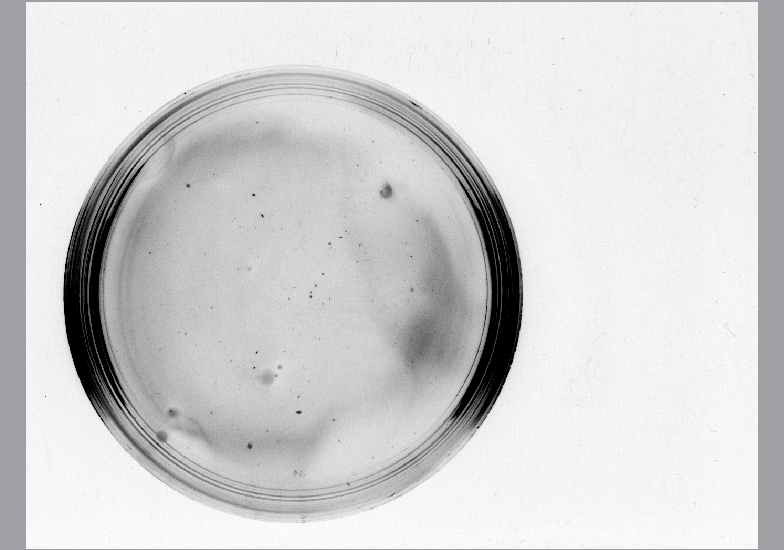

Supplement: Figure 1—figure supplement 3—source data 4. [file elife-95626-fig1-figsupp3-data4.zip › Raw images/Rep 3/TTC446_Rep3_DBD+_2.jpg]

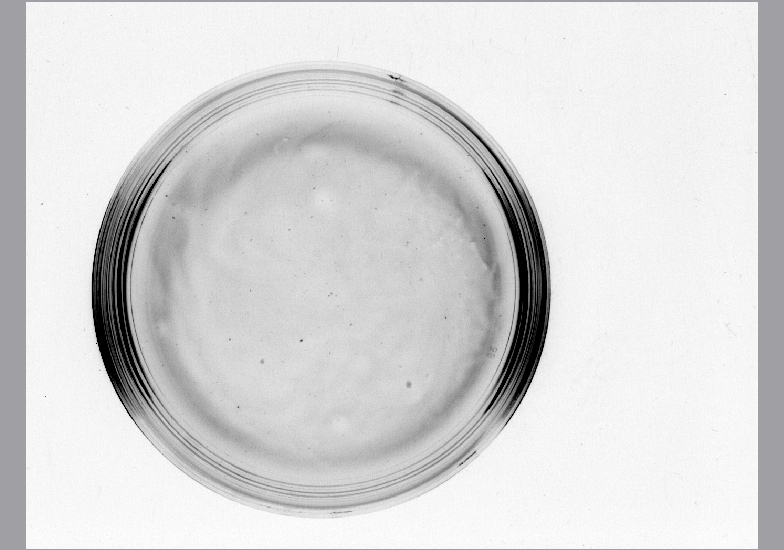

Supplement: Figure 1—figure supplement 3—source data 4. [file elife-95626-fig1-figsupp3-data4.zip › Raw images/Rep 3/TTC446_Rep3_DBD_1.jpg]

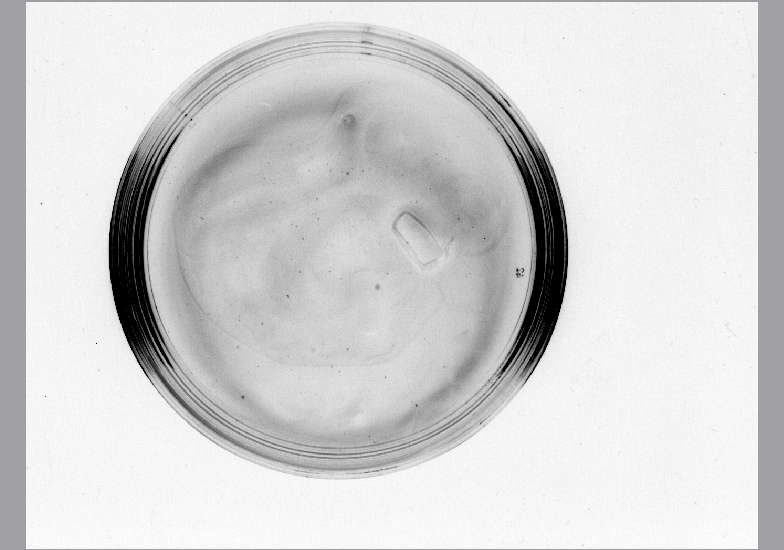

Supplement: Figure 1—figure supplement 3—source data 4. [file elife-95626-fig1-figsupp3-data4.zip › Raw images/Rep 3/TTC446_Rep3_DBD_2.jpg]

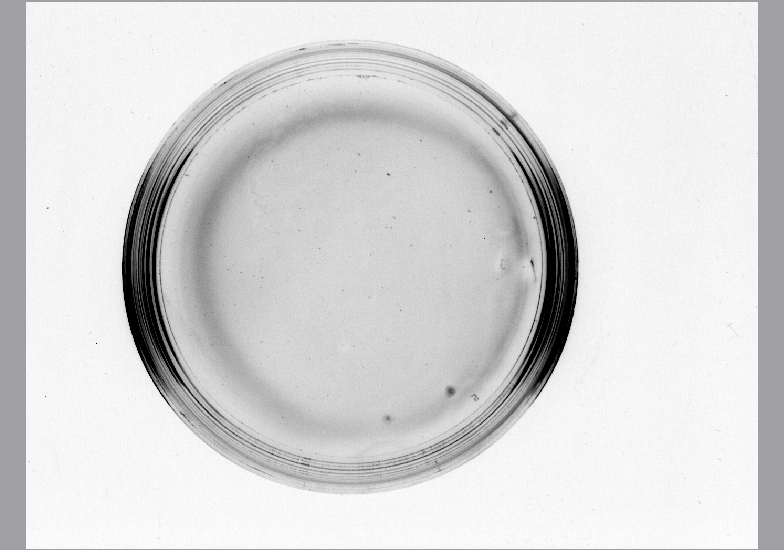

Supplement: Figure 1—figure supplement 3—source data 4. [file elife-95626-fig1-figsupp3-data4.zip › Raw images/Rep 3/TTC446_Rep3_iERG_1.jpg]

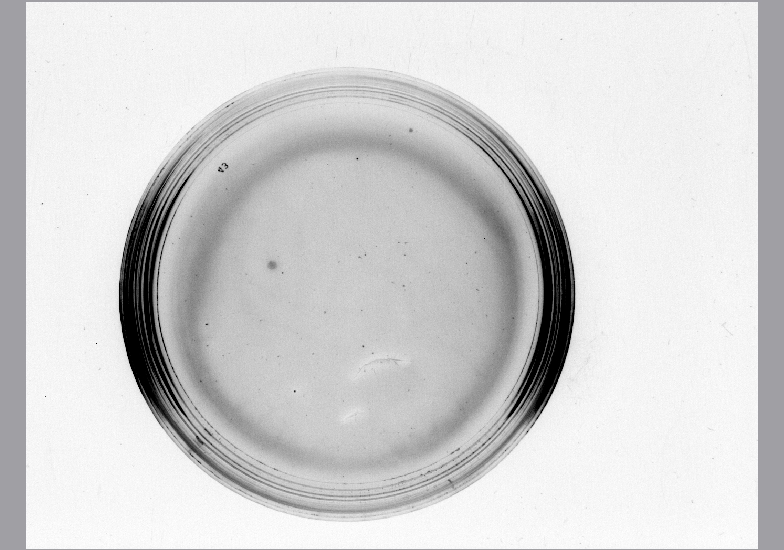

Supplement: Figure 1—figure supplement 3—source data 4. [file elife-95626-fig1-figsupp3-data4.zip › Raw images/Rep 3/TTC446_Rep3_iERG_2.jpg]
