# Supplementary material for: The DBD-α4 helix of EWSR1::FLI1 is required for GGAA microsatellite binding that underlies genome regulation in Ewing sarcoma
Source: eLife. 2026 Jun 15;13:RP95626. doi: 10.7554/eLife.95626 (PMC13268648; doi:10.7554/eLife.95626)
Supplement: Figure 1—figure supplement 3—source data 1. [file elife-95626-fig1-figsupp3-data1.zip › Labelled images/TTC446_uncropped.pdf]

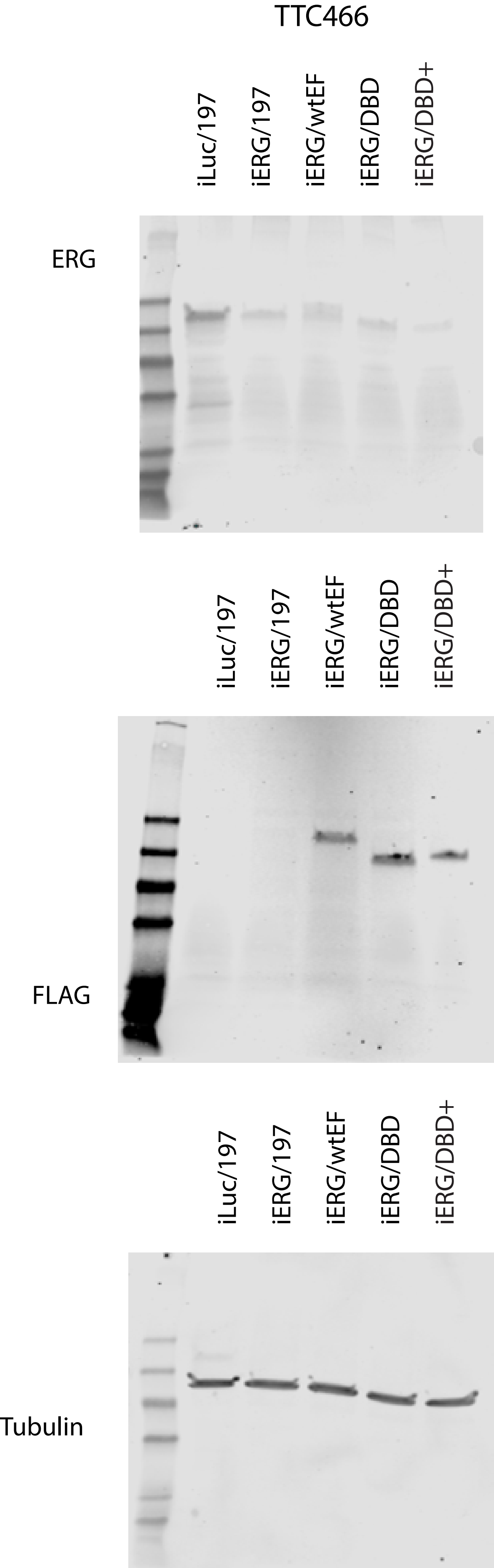
TTC466
iLuc/197
iERG/197
iERG/wtEF
iERG/DBD
iERG/DBD+
ERG
FLAG
Tubulin
