# Supplementary material for: The DBD-α4 helix of EWSR1::FLI1 is required for GGAA microsatellite binding that underlies genome regulation in Ewing sarcoma
Source: eLife. 2026 Jun 15;13:RP95626. doi: 10.7554/eLife.95626 (PMC13268648; doi:10.7554/eLife.95626)
Supplement: Peer review history

# Editor report: eLife Assessment

**DOI:** 10.7554/eLife.95626.4.sa0

**Authors:** Irwin Davidson

**Affiliations:** Institut de Génétique et de Biologie Moléculaire et Cellulaire France

**Keywords:** Convincing, Valuable

This paper investigates the Achilles' heel of an aggressive pediatric bone cancer known as Ewing sarcoma. The authors aimed to better understand how its previously undruggable drivers mediate oncogenic mechanisms using several omics approaches. Transcriptomic changes aligned with their findings provide **convincing** evidence for the role of a short alpha helix in the DNA binding domain of FLI1 in modulating binding to GGAA microsatellites and promoting enhancer activity. The study provides **valuable** new insights into the underlying oncogenic mechanisms in Ewing sarcoma.

# Referee report: Reviewer #1 (Public review)

**DOI:** 10.7554/eLife.95626.4.sa1

Summary:

Ewing sarcoma is an aggressive pediatric cancer driven by the EWS-FLI oncogene. Ewing sarcoma cells are addicted to this chimeric transcription factor, which represents a strong therapeutic vulnerability. Unfortunately, targeting EWS-FLI has proven to be very difficult and better understanding how this chimeric transcription factor works is critical to achieving this goal. Towards this perspective, the group had previously identified a DBD-𝛼4 helix (DBD) in FLI that appears to be necessary to mediate EWS-FLI transcriptomic activity. Here, the authors used multi-omic approaches, including CUT&tag, RNAseq, and MicroC to investigate the impact of this DBD domain. Importantly, these experiments were performed in the A673 Ewing sarcoma model where endogenous EWS-FLI was silenced, and EWS-FLI-DBD proficient or deficient isoforms were re-expressed (isogenic context). They found that the DBD domain is key to mediate EWS-FLI cis activity (at msat) and to generate the formation of specific TADs. Furthermore, cells expressing DBD deficient EWS-FLI display very poor colony forming capacity, highlighting that targeting this domain may lead to therapeutic perspectives.

Strengths:

The group has strong expertise in Ewing sarcoma genetics and epigenetics and also in using and analyzing this model (Theisen et al., 2019; Boone et al., 2021; Showpnil et al., 2022).

They aim at better understanding how EWS-FLI mediated its oncogenic activity, which is critical to eventually identifying novel therapies against this aggressive cancer.

They use the most recent state-of-the-art omics methods to investigate transcriptome, epigenetics, and genome conformation methods. In particular, Micro-C enables achieving up to 1kb resolved 3D chromatin structures, making it possible to investigate a large number of TADs and sub-TADs structures where EWS-FLI1 mediates its oncogenic activity.

They performed all their experiments in an Ewing sarcoma genetic background (A673 cells) which circumvents bias from previously reported approaches when working in non-orthologous cell models using similar approaches.

Weaknesses:

The main weakness stems from the poor reproducibility of the Micro-C data. Indeed, the distances and clustering observed between replicates appear to be similar to, or even greater than, those observed between biological conditions. For instance, in Figure 1B, we do not observe any clear clustering among DBD1, DBD2, DBD+1, and DBD+2. Although no further experiments were performed, the authors tempered their claims by rephrasing aspects related to this issue and the reviewer also acknowledged that the transcriptomic data are convincing and support their findings.

Regarding DBD stability and the cycloheximide experiments requested to rule out any half-life bias of DBD (as higher stability of the re-expressed DBD+ could also partially explain the results independently of a 3D conformational change), the reviewer acknowledged that the WB, RNA-seq data and agar assays presented by the authors appear reproducible across experiments.

# Author comment: Author response

**DOI:** 10.7554/eLife.95626.4.sa2

**Authors:** Ariunaa Bayanjargal, Cenny Taslim, Iftekhar A Showpnil, Julia Selich-Anderson, Jesse C Crow, Runwei Zhou, Stephen L Lessnick, Emily Rose Theisen

**Affiliations:** Nationwide Children's Hospital Columbus United States; Nationwide Children's Hospital Columbus United States; Nationwide Children's Hospital Columbus United States; Nationwide Children's Hospital Columbus United States; Nationwide Children's Hospital Columbus United States; Nationwide Children's Hospital Columbus United States; Huntsman Cancer Institute, University of Utah Salt Lake City United States; Nationwide Children's Hospital Columbus United States

The following is the authors’ response to the previous reviews

**Public Review:**

**Reviewer #1 (Public review):**

Ewing sarcoma is an aggressive pediatric cancer driven by the EWS-FLI oncogene. Ewing sarcoma cells are addicted to this chimeric transcription factor, which represents a strong therapeutic vulnerability. Unfortunately, targeting EWS-FLI has proven to be very difficult and better understanding how this chimeric transcription factor works is critical to achieving this goal. Towards this perspective, the group had previously identified a DBD-𝛼4 helix (DBD) in FLI that appears to be necessary to mediate EWS-FLI transcriptomic activity. Here, the authors used multi-omic approaches, including CUT&tag, RNAseq, and MicroC to investigate the impact of this DBD domain. Importantly, these experiments were performed in the A673 Ewing sarcoma model where endogenous EWS-FLI was silenced, and EWS-FLI-DBD proficient or deficient isoforms were re-expressed (isogenic context). They found that the DBD domain is key to mediate EWS-FLI cis activity (at msat) and to generate the formation of specific TADs. Furthermore, cells expressing DBD deficient EWS-FLI display very poor colony forming capacity, highlighting that targeting this domain may lead to therapeutic perspectives.

This new version of the study comprises as requested new data from an additional cell line. The new data has strengthened the manuscript. Nevertheless, some of the arguments of the authors pertaining to the limitations of immunoblots to assess stability of the DBD constructs or the poor reproducibility of the Micro C data remain problematic. While the effort to repeat MicroC in a different cell line is appreciated, the data are as heterogeneous as those in A673 and no real conclusion can be drawn. The authors should tone down their conclusions. If DBD has a strong effect on chromatin organization, it should be reproducible and detectable. The transcriptomic and cut and tag data are more consistent and provide robust evidence for their findings at these levels.

We agree that the Micro-C data have more apparent heterogeneity within and across cell lines as compared to other analyses such as our included CUT&Tag and RNA-seq. We addressed the possible limitations of the technique as well as inherent biology that might be driving these findings in our previous responses. Despite the poor clustering on the PCA plots, our analysis on differential interacting regions, TADs and loops remain consistent across both cell lines. We are confident that these findings reflect the context of transcriptional regulation by the constructs, therefore the role of the alpha-helix in modulating chromatin organization. To address the concerns raised by the editors and reviewers for the strength of the conclusions we drew from the Micro-C findings we have made changes to the language used to describe them throughout the manuscript. Find these changes outlined below.

• On lines 70-71, "is required to restructure" was changed to "is implicated in restructuring of"

• On line 91, "is required for" was changed to "participates in"

• On line 98, "is required for" changed to "is potentially required for"

• On line 360-361, "is required for restructuring" changed to "participates in restructuring"

Concerning the issue of stability of the DBD and DBD+ constructs, a simple protein half-life assay (e.g. cycloheximide chase assay) could rule out any bias here and satisfactorily address the issue.

While we generally agree that a cycloheximide assay is a relatively simple approach to look at protein half-life, as we discussed last me the assays included in this paper are performed at equilibrium and rely on the concentration of protein at the me of the assay. This is particularly true for assays involving crosslinking, like Micro-C. As discussed in our prior response, western blots are semi quantitative at best, even when normalized to a housekeeping protein. In analyzing the relative protein concentration of DBD vs. DBD+ with relative protein intensities first normalized to tubulin and using the wildtype EWSR1::FLI1 rescue as a reference point, we find that there is no statistical difference in the samples used for micro-C here (Author responseimage 1A) or across all of the samples that we have used for publication (Author response image 1B). This does show that DBD generally has more variable expression levels relative to wildtype EWSR1::FLI1, and this is consistent with our experience in the lab.

Nonetheless, we did attempt to perform the requested cycloheximide chase experiment to determine protein stability. Unfortunately, despite an extensive number of troubleshooting attempts, we have not been able to get good expression of DBD for these experiments. The first author who performed this work has left the lab and we have moved to a new lab space since the benchwork was performed. We continue to try to troubleshoot to get this experimental system for DBD and DBD+ to work again. When we tried to look at stability of DBD+ following cycloheximide treatment, there did appear to be some difference in protein stability (Author response image 2). However, these conditions are not the same conditions as those we published, they do not meet our quality control standards for publication, and we are concerned about being close to the limit of detection for DBD throughout the later timepoints. Additional studies will be needed with more comparable expression levels between DBD and DBD+ to satisfactorily address the reviewer concerns.

**Author response image 1.** Expression Levels of DBD and DBD+ Across Experiments. Expression levels of DBD and DBD+ protein based on western blot band intensity normalized by tubulin band intensity. Expression levels are relative to wildtype EWSR1::FLI1 rescue levels and are calculated for (A) A673 samples used for micro-C and (B) all published studies of DBD and DBD+. Data show mean and standard deviation for samples. P-values were calculated with an unpaired t-test.

**Author response image 2.** CHX chase assay to determine the stability of DBD and DBD+. (A) Knock-down of endogenous EWSR1::FLI1 detected with FLI1 ab and rescue with DBD and DBD+ detected with FLAG ab. (B) CHX chase assay to determine the stability of DBD and DBD+ in A-673 cells with quantification of the protein levels (n=3). Error bars represent standard deviation. The half-lives (t1/2) of DBD and DBD+ were listed in the table.

Suggestions:

The Reviewing Editor and a referee have considered the revised version and the responses of the referees. While the additional data included in the new version has consolidated many conclusions of the study, the MicroC data in the new cell line are also heterogeneous and as the authors argue, this may be an inherent limitation of the technique. In this situation, the best would be for the authors to avoid drawing robust conclusions from this data and to acknowledge its current limitations.

As discussed above, we have changed the language regarding our conclusions from micro-C data to soften the conclusions we draw per the Editor’s suggestion.

The referee and Reviewing Editor also felt that the arguments of the authors concerning a lack of firm conclusions on the stability of EWS-FLI1 under +/-DBD conditions could be better addressed. We would urge the authors to perform a cycloheximide chase type assay to assess protein half-life. These types of experiments are relatively simple to perform and should address this issue in a satisfactory manner.

As discussed above, we do not feel that differences in protein stability would affect the results here because the assays performed required similar levels of protein at equilibrium. Our additional analyses in this response shows that there are not significant differences between DBD and DBD+ levels in samples that pass quality control and are used in published studies. However, we attempted to address the reviewer and editor comments with a cycloheximide chase assay and were unable to get samples that would have passed our internal quality control standards. These data may suggest differences in protein stability, but it is unclear that these conditions accurately reflect the conditions of the published experiments, or that this would matter with equal protein levels at equilibrium.
